# Mechanism insights and therapeutic intervention of tumor metastasis: latest developments and perspectives

**DOI:** 10.1038/s41392-024-01885-2

**Published:** 2024-08-02

**Authors:** Xiaoli Shi, Xinyi Wang, Wentao Yao, Dongmin Shi, Xihuan Shao, Zhengqing Lu, Yue Chai, Jinhua Song, Weiwei Tang, Xuehao Wang

**Affiliations:** 1 Hepatobiliary Center, The First Affiliated Hospital of Nanjing Medical University, Key Laboratory of Liver Transplantation, Chinese Academy of Medical Sciences; NHC Key Laboratory of Hepatobiliary Cancers, Nanjing, Jiangsu China; 2 School of Medicine, Southeast University, Nanjing, Jiangsu China; 3 The First Clinical Medical College, Nanjing Medical University, Nanjing, Jiangsu China; 4 Department of Urology, Suzhou TCM Hospital Affiliated to Nanjing University of Chinese Medicine, Suzhou, Jiangsu China; 5 Department of Medical Oncology, Shanghai Changzheng Hospital, Shanghai, China; 6 The Fourth Clinical Medical College, Nanjing Medical University, Nanjing, Jiangsu China

**Keywords:** Metastasis, Metastasis

## Abstract

Metastasis remains a pivotal characteristic of cancer and is the primary contributor to cancer-associated mortality. Despite its significance, the mechanisms governing metastasis are not fully elucidated. Contemporary findings in the domain of cancer biology have shed light on the molecular aspects of this intricate process. Tumor cells undergoing invasion engage with other cellular entities and proteins en route to their destination. Insights into these engagements have enhanced our comprehension of the principles directing the movement and adaptability of metastatic cells. The tumor microenvironment plays a pivotal role in facilitating the invasion and proliferation of cancer cells by enabling tumor cells to navigate through stromal barriers. Such attributes are influenced by genetic and epigenetic changes occurring in the tumor cells and their surrounding milieu. A profound understanding of the metastatic process’s biological mechanisms is indispensable for devising efficacious therapeutic strategies. This review delves into recent developments concerning metastasis-associated genes, important signaling pathways, tumor microenvironment, metabolic processes, peripheral immunity, and mechanical forces and cancer metastasis. In addition, we combine recent advances with a particular emphasis on the prospect of developing effective interventions including the most popular cancer immunotherapies and nanotechnology to combat metastasis. We have also identified the limitations of current research on tumor metastasis, encompassing drug resistance, restricted animal models, inadequate biomarkers and early detection methods, as well as heterogeneity among others. It is anticipated that this comprehensive review will significantly contribute to the advancement of cancer metastasis research.

## Introduction

Globally, cancer stands as the predominant cause of mortality, with the World Health Organization attributing over 90% of cancer-related deaths to metastasis. Annually, ~18 million individuals receive a cancer diagnosis, and of these, an estimated 10–15% will manifest metastases. Prevalent cancers encompass those of the breast, lung, colon, rectum, and prostate. From 1991 to 2019, there was a notable 32% decline in cancer mortality in the United States.^1^ This decline is predominantly ascribed to reductions in lung cancer-related deaths. Concurrently, death rates from melanoma have witnessed a significant decrease, indicative of enhanced survival rates in metastatic melanoma cases, a consequence of varied factors including cancer preventive measures, the inception of targeted drug therapies, and the application of immune checkpoint inhibitors (ICIs). Additionally, strides in early cancer detection and innovative treatment modalities have been contributory. As a result of this declining death rate, the cancer survivor population has expanded. While these trends underscore advancements in oncological interventions, considerable challenges persist. Certain cancers, including pancreatic, liver, uterine, and sarcoma, have demonstrated stagnant or even escalating death rates. Alarmingly, despite seven decades of dedicated cancer therapeutic research, the five-year survival indices for metastatic disease sufferers remain dismal, oscillating between 5 and 30% for solid neoplasms.^1^ This stagnation in survival rates stems from the recurrent observation that preclinical therapeutic successes do not invariably herald clinical benefits for metastatic cancer patients.^2^ Evident metastasis, barring a few exceptions, remains refractory to prevailing therapeutic regimens, accentuating the enormity of the challenge.

While primary tumors might be amenable to curative interventions like local surgery or radiation therapy, metastasis exhibits a systemic disposition. Its management necessitates an amalgamation of chemotherapy, targeted therapies, and immunotherapy, among others. Prolonged cancer research endeavors have conferred invaluable insights into tumorigenesis molecular paradigms. Processes encompassing tumorigenesis, cellular migration, and metastasis are integral to the oncological spectrum. Over recent decades, synergistic efforts between basic and clinical oncology have engendered significant advancements in metastatic cancer therapeutics. The amalgamation of next-generation sequencing techniques with sophisticated computational data interpretation has redefined our perception of the genomic foundation of cancer onset and progression, offering valuable insights into disease trajectory and drug responsiveness. This heightened understanding, complemented by the advent of therapies targeting the cancer genome, has propelled the clinical incorporation of genomic diagnostic methodologies in cancer management. Innovative clinical trial frameworks like umbrella, basket, and platform trials have streamlined drug approvals. But a deep understanding of the biological mechanisms of the metastasis process remains essential for developing effective treatment strategies. This review delves into recent developments concerning metastasis-associated genes, the tumor microenvironment (TME), metabolic processes, peripheral immunity, and mechanical forces and cancer metastasis. In addition, we combine recent advances to highlight the barriers and prospects for developing effective interventions to combat metastasis.

## History of metastatic cancer

**Fig. 1:**
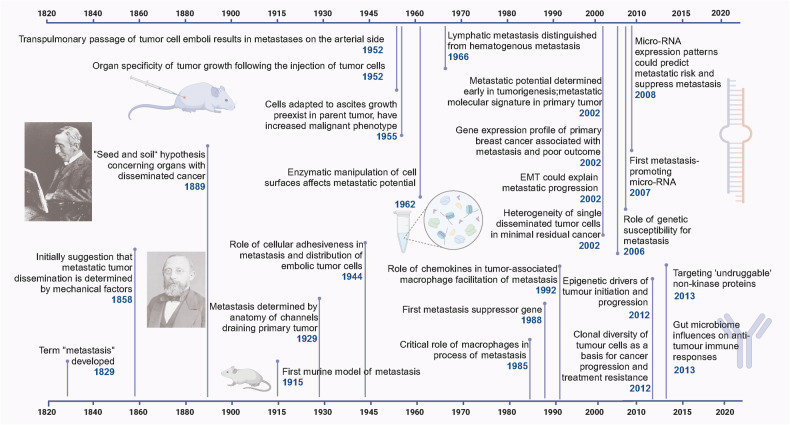
The significant history and progress of cancer metastasis. Timeline of pivotal discoveries in cancer metastasis research from 1820 to 2020, showcasing milestones such as the “Seed and soil” hypothesis, micro-RNA’s role, organ specificity, genetic and epigenetic influences, and the impact of cellular interactions and the gut microbiome on metastatic progression. Created with BioRender.com

The term metastasis was coined in 1829 by Jean Claude Recamier to describe the spread of cancer from one organ to another. In 1889, Stephen Paget proposed the seed and soil hypothesis, which suggested that metastasis depends on the interaction between tumor cells (seed) and organ microenvironments (soil). In the late 1970s and early 1980s, experimental studies by Fidler and others confirmed the biological heterogeneity and clonal selection of metastatic cells, as well as the role of host factors in organ-specific metastasis. Since the 1980s, molecular and cellular mechanisms of metastasis have been elucidated, such as the role of angiogenesis, adhesion molecules, proteolytic enzymes, chemokines, and immune cells. In the late 1990s, the isolation of tumor suppressor genes accelerated studies on genetic alterations in human cancer cells. The management of cancer metastases evolved rapidly during the last 30 years due to the development of technology and emergence of novel therapy. With the advancing understanding of molecular pathway and biological behavior of oncogenesis and tumor metastasis, novel targeted therapy including tyrosine-kinase inhibitors and immunotherapy are applied to cancer metastases. The process of metastasis is a multifaceted and inefficient phenomenon, encompassing various sequential steps and stochastic components.^3,4^ It stands as the primary cause of cancer-related mortality and poses a significant challenge in terms of diagnosis and treatment. Advancements in this domain have the potential to revolutionize the treatment paradigm. Gaining insights into the historical and current landscape of metastatic cancer can greatly aid in accurate diagnoses and effective therapeutic interventions (Fig. 1).

## Procedure of tumor metastasis

There are two widely recognized models of tumor metastasis in terms of time line. One is a linear model, in which metastatic tumor cells colonize different organs at what is widely believed to be an advanced stage, and the other is a parallel model, in which cancer cells spread at an early stage, which is distinguished by determining how much gene the metastatic cells share with the primary tumor and whether they are independent of the evolutionary pattern of the primary tumor cells. The biological process of tumor metastasis is called the invasion and metastasis cascade. These include preparation, local invasion, intravasation, and survival in the circulation, extravasation, colonization, and reactivation.^5^ Regardless of the mode of tumor metastasis, the premise is the selection of metastatic tumor cells and the occurrence of their cloning, which is called the “preparation” process of tumor metastasis. Epithelial mesenchymal transition (EMT) is a cell spectrum with varying degrees of epithelial and interstitial characteristics. In aggressive tumors, the mixed epithelial/mesenchymal state, E/M state, dominates tumor cells, and the progression of EMT is indispensable for the acquisition of stem cell properties and the increase of aggressiveness of tumor cells. The formation of tumor-related microvessels provides a prerequisite for intravasation, because the blood vessels generated by cancer cells are highly curved, easily leaky and in a period of constant remodeling. The weak interaction between endothelial cells adjacent to blood vessels and the lack of pericytes provide opportunities for invasive tumor cells. Platelet plays an extremely important role in intravasation and circulation stage. On the one hand, it releases growth factors and TGF-β to drive EMT process and entrusts circulating tumor cells (CTC) with powerful invasion force. On the other hand, platelets and CTC interact and bind into large emboli under the mediation of cancer cell tissue factors and selectin, which not only makes CTC free from the pressure of blood flow shear force, but also makes the tumor surface major histocompatibility complex (MHC) transfer into the cell, interfering with immune recognition and escaping natural killer (NK) cell-mediated cytotoxicity. Extravasation is to penetrate the blood vessel wall of the distant organ, so that the tumor cells are in direct contact with the tissue parenchyma, and prepare for the distant colonization. Most cells that successfully exosmose enter a dormant state in the form of micrometastases, where there is insufficient growth factor signaling and activated vascular signaling, while the immunosuppressive microenvironment is still functioning. However, diffuse tumor cells (DTC) in the dormant state can inhibit the activation ligands of various NK cells and evade the clearance of NK cells. The ability of DTC to get rid of dormancy and start active proliferation may be more dependent on the non-autonomous mechanism of cells. After the accumulation of cytokine signals released by primary cancer cells and the completion of the remodeling of microenvironment by myeloid-derived suppressor cells (MDSC), DTC with CSC characteristics enters the reactivation stage under the stimulation of inflammation or trauma.^5–7^

## Regulation of genes in metastatic cancer

### Gene mutation on tumor metastasis

In the realm of metastasis-associated gene research, there is a pronounced focus on genes that promote metastasis. Driver genes are thought to be highly species-specific with respect to tumorigenesis, with mutations triggering only a few tumor types.^8^ For instance, the association between key driver gene mutations and colorectal cancer (CRC) metastasis has been a subject of extensive investigation. Mutations in genes such as *KRAS*, *p53*, *SMAD4*, and *BRAF* have been identified as significant contributors to CRC metastasis.^9^ The gain-of-function mutant P53 protein, in particular, has been linked to enhanced invasion, metastasis, and migration of cancer cells.^10,11^ There is a prevailing hypothesis that cells responsible for metastasis within primary tumors display genetic homogeneity concerning functional driver gene mutations, more so when metastases emerge from a singular primary source.^12,13^ In contrast, Hu et al.^14^ analyzed data from nearly 200 tumors and discerned that ectopic metastasis was more intricately tied to tumor resistance as opposed to metastasis-driving mutations when compared to primary tumor evolution. Numerous other genes implicated in promoting primary tumor metastasis have been documented in the literature. Of course, tumors are highly heterogeneous, and cells also contain a variety of other mutations called passenger mutations,^15^ they arise from random mutations in sequences that do not directly lead to disease, and are prone to occur because of exposure to mutagenic processes and lack of repair, thus providing a more favorable environment of fitness for metastasis of malignant cells.^16^

**Fig. 2:**
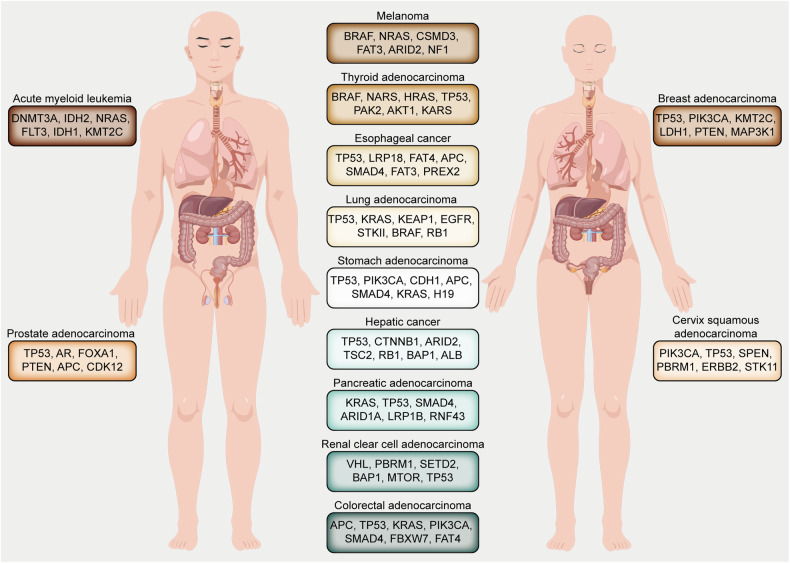
The chart categorizes a range of cancers, including melanoma, thyroid adenocarcinoma, and others, and identifies the most frequently mutated driver genes associated with each. These mutations are crucial for the development and progression of the respective cancers and may serve as potential therapeutic targets. It underscores the importance of understanding these mutations for the development of personalized medicine and targeted cancer therapies. Created with Figuredraw.com

Oncogenic mutations endow malignant cells with capabilities such as rapid proliferation, immune system evasion, tissue invasion, and environmental modulation for their benefit.^8^ Notably, *Rat sarcoma (Ras)* genes, which are proto-oncogenes, conventionally participate in cell signaling to govern cell growth and differentiation. However, mutations in *RAS* genes result in persistent activation, thereby promoting unchecked cellular growth. Research indicates that around 30% of human malignancies are attributed to *RAS* gene mutations, with their products consistently in an active state. *K-Ras* mutations are prevalent in leukemia, lung, rectal, and pancreatic cancers, with rectal cancer patients exhibiting mutations in 30–35% of cases. Additionally, *ras* can synergize with other oncogenes or tumor suppressors to augment cancer progression. In hepatocellular carcinoma (HCC), for instance, *FoxM1* overexpression, a transcription factor for cell cycle genes, intensifies *Ras*-driven HCC progression and metastasis.^17^ Conversely, in HCC, suppressor genes such as *RASAL1*, *DAB2IP*, and *NF1 RAS GAP* curtail the Ras pathway’s limitless activation, subsequently inhibiting tumor metastasis.^18^ In esophageal cancer, both *Rho kinase (ROCK)* and *Ras* have been linked to metastatic cell behavior, fostering tumor metastasis and invasion.^19^ In CRC, approximately half of the cases exhibit activating point mutations in the *Ki-ras* proto-oncogene. Shirasawa et al.^20^ observed morphological alterations in cells with activated *Ki-ras* gene mutations, with these cells exhibiting reduced adhesive growth and diminished *c-myc* expression. In pancreatic ductal adenocarcinoma (PDAC), *p53* mutations upregulate *hnRNPK* expression, a splicing regulator, amplifying *KRAS* activity and hence promoting invasion and PDAC metastasis.^21^ Additionally, in PDAC, the *K-Ras* mutant collaboratively functions with *CDK5* and its activator, prompting the malignant progression of pancreatic cancer cells.^22^ Moreover, the *BRD2* gene advances drug resistance and metastasis in adult T-cell lymphoblastic lymphoma (T-LBL) via the RasGRP1/Ras/ERK signaling pathway.^23^ In metastatic prostate cancer, several genetic alterations, including *CDK12* mutations, *TP53* inactivation, and *BRCA2* inactivation, have been associated with disease promotion.^24^ In cervical cancer, the *CIP2A* gene’s interaction with *H-Ras* activates the MEK/ERK signaling pathway, promoting epithelial–mesenchymal transition and cancer progression.^25^ Additionally, the *R-Ras* oncogene, through the activation of the PI3-K/Akt/mTOR signaling pathway, is instrumental in advancing cervical cancer to metastatic stages.^26^ Even though *BRAF/KRAS* mutations are detected in peripheral ovarian cancer tissues, NRAS mutations are exclusive to tumor tissues, signifying a crucial association of *Ras* oncogenes with invasive serous carcinoma.^27^ In breast cancer brain metastasis, genes such as *CXCL12*, *MMP2*, *MMP11*, *VCAM1*, and *MME* are associated with tumor progression, distinguishing primary brain cancer from metastatic breast cancer in the brain.^28^ Fasching et al.^29^ identified a high frequency of germ line mutations in *BRCA1/2* and other breast cancer predisposition genes in metastatic breast cancer. These genetic variances in tumor characteristics provide insights for ongoing and future targeted therapeutic research, even if the prognostic outcomes remain consistent. Moreover, Metastases exhibit more gene copy number amplification and chromosomal abnormalities in certain cancers compared to primary tumors, e.g., colon cancer metastasis is accompanied by progressive copy number deletion of the oncogene PTEN and progressive copy number amplification of the oncogene *MYC*.^30^ Fig. 2 lists important mutation-driving genes for some common types of cancer. Mutations in driving genes maintain proliferation signals in malignant cells and trigger invasion and metastasis.

### The effect of non-coding RNAs on tumor metastasis

Non-coding RNAs are pivotal in numerous cellular functions, eliciting considerable interest in the fields of molecular biology and genetics. They encompass rRNA, tRNA, snRNA, snoRNA, microRNA (miRNA), long non-coding RNA (lncRNA), circular RNA (circRNA), and other functionally defined RNAs, as well as those whose roles are yet to be elucidated. The pertinence of non-coding RNA in various phases of cancer progression has made it a focal point in contemporary oncological research.^31^ For instance, 5-methylcytosine, an integral component of the epigenetic regulation network, has been associated with several cellular activities and broader pathologies, including cell migration and cancer metastasis.^32^ Furthermore, Park et al.^33^ determined that the lncRNA *Neat1* directly interacts to assemble the PGK1/PGAM1/ENO1 complex, subsequently facilitating the glycolysis pathway and promoting tumor metastasis. The subsequent sections will elucidate the modulatory effects of non-coding RNA on metastasis across diverse cancer types.

Exosomal miRNA modulation is pivotal in HCC progression. The inhibition of *miR-374a-5p* via exosomes restricts the proliferation, migration, and invasion of HCC cells.^34^ Additionally, *miR-346* has been observed to advance liver cancer proliferation, migration, and invasion through its interaction with *FBXL2*.^35^ There is potential for future HCC research to consider *miR-346* as a diagnostic marker and therapeutic target. In mesenchymal cells, elevated levels of miRNAs *miR-99a*, *miR-100*, and *miR-125b* promote migration and invasion.^36,37^ In the context of prostate cancer, miRNAs have a pronounced effect on tumor metastasis. Specifically, the silencing of the *miRNA-338-5p/-421* axis in *Spink1*-positive prostate cancer patients, as well as *miR-34b*, facilitates tumor metastasis.^38,39^ Sun et al.^40^ discerned that *miR-G-10* expression in cervical cancer cells markedly enhances cell migration, invasion, anti-anoikis capabilities in vitro, and lung metastasis in vivo. In ovarian cancer, miR-101 regulation amplifies EMT and metastasis by modulating pro-transition associated RNA synthesis.^41^ LncRNA also plays a determinative role in various cancers. In non-small cell lung carcinoma (NSCLC), lncRNA *PKMYT1AR* counteracts β-catenin protein ubiquitination degradation facilitated by the PKMYT1AR/miR-485-5p/PKMYT1 axis, maintaining CSC and propelling NSCLC metastasis.^42^ LncRNA *IGFBP4-1*, identified to have elevated expression in lung cancer tissues, may foster cell proliferation and metastasis by modifying cellular energy metabolism.^43^ In CRC, lncRNA *CCAT2* represses *miR-145* augmentation and inversely modulates *miR-21*, facilitating CRC cell proliferation and differentiation. Moreover, *Bcl-2* specific siRNA inhibition can specifically curtail target gene expression, promoting pancreatic cancer metastasis.^44^ LncRNA *PCAT19’s* interaction with *HNRNPAB* in prostate cancer and the androgen insensitivity or AR antagonist activation of lncRNA *NEAT1* in prostate cancer cells stimulate prostate cancer growth and metastasis by influencing associated cell cycle genes.^45,46^ Recent studies have highlighted the role of circRNAs in human cancers. Zheng et al.^47^ identified a novel circRNA, *circPPP1R12A*, derived from the *PPP1R12A* gene.^43^ *CircPPP1R12A* encompasses a 216 nt open reading frame encoding a 73 amino acid peptide (circPPP1R12A-73aa). This peptide has been shown to enhance invasion and metastasis capabilities in colon cancer through the Hippo-YAP signaling pathway. Another circRNA, *circFBXW7*, encodes a 185 amino acid peptide (*FBXW7-185aa*), which has been demonstrated to restrict the metastasis of triple-negative breast cancer (TNBC) cells by augmenting *FBXW7* abundance and inducing *c-Myc* degradation.^48^ These revelations regarding circRNA translation and function provide promising avenues for metastatic cancer treatment.

### The effects of modifications on tumor metastasis

RNA modifications can impact the stability, splicing, translation, and modification of RNA molecules, thereby playing a crucial role in tumor metastasis. M6A is concurrently upregulated during tumor metastasis and not only regulates the expression of target genes but also influences immune cells like reprogrammed macrophages to facilitate tumor metastasis.^49^ M5C modification of mitochondrial RNAs promotes tumor metastasis by modulating metabolic plasticity.^50^ Th*e MYC* downstream effector, 7-Methylguanosine methyltransferase *WDR4*, facilitates HCC metastasis.^51^ *MFAP2* regulates N1-methyladenosine to drive CRC progression.^48^ DNA methylation entails a covalent modification that exclusively occurs at the C5 position of cytosine within the cytosine guanine dinucleotide in higher eukaryotes. In this process, cytosine is conjugated with a methyl group, resulting in 5-methylcytosine (5-mC). This modification is facilitated by DNA methyltransferase.^52^ Notably, NOP2/Sun RNA methyltransferase 3 dependent RNA modifications, specifically -5-methylcytosine (m5C) and its derivative 5-formylcytosine, have been implicated in promoting mitochondrial mRNA translation, thereby enhancing tumor metastasis.^53^ In breast cancer, the progression and metastatic capability are heightened by BAF155/SMARCC1 methylation, a core component of the *SWI/SNF* chromatin remodeling/tumor suppressor complex. This methylation targets BAF155 to genes in the c-Myc pathway.^54^ Moreover, the histone methyltransferase and EMT inducer, Zeste enhancer homolog 2 (*EZH2*), undergoes asymmetric dimethylation at R342 (meR342-EZH2) by *PRMT1*. This modification results in diminished expression of *EZH2* target genes, augmenting EMT, invasion, and metastasis in breast cancer cells.^55^ Apart from these, the strong correlation between reader YTHDF1 and tumor metastasis has been extensively demonstrated.^56,57^ Although the role of methylated eraser in tumor metastasis has received limited attention, elevated levels of eraser in cervical squamous cell carcinoma indicate a higher propensity to metastasis.^58^ Additionally, post-translational modification of proteins plays a pivotal role in tumor metastasis by regulating protein structure, stability, and processes such as invasion, migration, and angiogenesis. In lung cancer, the acetylation level of histone H3K27ac is closely associated with the invasiveness and metastatic potential of tumor cells.^59^ Similarly, ubiquitination, which plays a crucial role in protein degradation, impacts the metastatic ability of CRC by modulating p53 protein function.^60^ Moreover, multiple studies have demonstrated that phosphorylation of EGFR proteins significantly influences HCC metastasis.^61^

### Navigating the signaling pathways in tumor metastasis

**Fig. 3:**
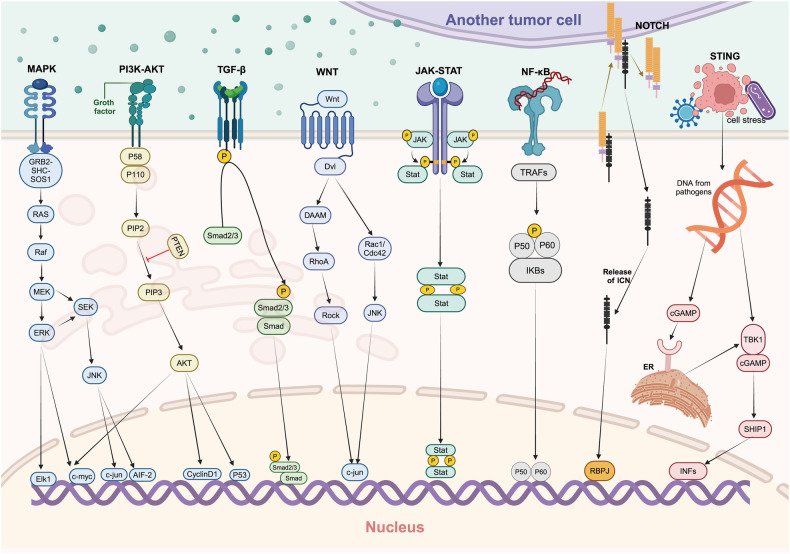
Classic signaling pathways related to tumor metastasis. Among them, MAPK, PI3K-AKT, TGF-β, JAK-STAT are phosphorylation-based signaling pathways that are cascaded and amplified by extracellular activating factors into the cell, ultimately entering downstream nuclear transcription regulation. Wnt and NF-κB signaling pathway are signal forms based on phosphorylation and protein ubiquitination degradation, which are also widely expressed. Notch is a protein shear-dependent signaling pattern. A significant feature that distinguishes the STING pathway from several other immune signaling mechanisms is that its activation is triggered by DNA, thus lacking any pathogen specificity. Created with BioRender.com

The intricacy of tumor metastasis can be partly attributed to the activation and regulation of signaling pathways. Here we explores the key signaling pathways involved in tumor metastasis (Fig. 3), revealing their specific mechanisms of action, interactions specificity.

### The role of signaling pathways in tumor metastasis

The NF-κB signaling pathway plays a multifaceted role in tumor metastasis by promoting the invasiveness and metastatic capacity of tumor cells, maintaining pro-survival signals, and interacting with immune cells in the tumor microenvironment, leading to a chronic inflammatory state associated with malignant transformation, angiogenesis, metastasis, and therapy resistance.^62^ Fan et al. have extensively discussed the multiple roles of NF-κB in tumor metastasis.

Both the PI3K/Akt and MAPK signaling pathways can enhance the migration capabilities of tumor cells, thus promoting tumor metastasis. Joseph et al.^63^ found that inhibition of the PI3K/Akt pathway significantly reduced the metastatic potential of tumor cells. Johnson et al.^64^ discovered that the activation of the PI3K pathway promotes metabolic reprogramming of tumor cells, providing energy and biosynthetic precursors that support tumor invasion and metastasis. The activation of the MAPK signaling pathway similarly promotes the invasiveness and metastatic capacity of cancer cells, with Chen et al.^65^ showing that inhibition of the MAPK pathway significantly reduces the metastatic ability of breast cancer. Furthermore, both the PI3K/Akt and MAPK signaling pathways closely interact with the tumor microenvironment, with Liu et al.^66^ finding that PI3K/Akt promotes tumor metastasis by regulating tumor-associated fibroblasts (CAFs) and tumor angiogenesis. The MAPK signaling pathway also interacts with immune cells in the tumor microenvironment, affecting tumor metastasis and immune evasion, with Larrayoz et al.^67^ finding that inhibition of the MAPK signaling pathway reduces immunosuppressive cells in the tumor microenvironment, thereby enhancing antitumor immune responses and reducing tumor metastasis.

The Notch and Hedgehog signaling pathways exhibit distinct characteristics in tumor metastasis, playing a pivotal role in determining cellular fate and tissue morphogenesis during embryonic development. Dysregulated activation of these pathways in tumors can result in aberrant cell differentiation and self-renewal of cancer stem cells, thereby facilitating tumor metastasis.^68–72^ The Notch signaling pathway transmits signals via direct intercellular contact, thereby facilitating the interaction between cancer cells within the tumor microenvironment and promoting tumor invasion and metastasis.^73^ Certain tumor types, such as basal cell carcinoma (Hedgehog pathway) and T cells of acute lymphocytic leukemia (Notch), exhibit close associations except for the activation of these signaling pathways, which offer specificity for targeted treatment strategies.^74–76^ Importantly, despite their unique involvement in tumorigenesis, Notch and Hedgehog pathways also play crucial roles in normal physiological processes; thus, careful consideration is required regarding their potential side effects and safety during the development of cancer therapeutics.

The JAK/STAT, Wnt/β-catenin pathways influence tumor cell migration, invasion, and adhesion through various mechanisms, while also regulating tumor immune suppression, cytokine levels, and interacting with lipid and glucose metabolism, collectively promoting tumor metastasis.^77^ The JAK/STAT pathway is implicated in the regulation of the inflammatory response, while inflammation plays a pivotal role in tumor metastasis.^78^ Furthermore, within the JAK/STAT pathway, there exist negative regulatory signals such as SOCS1, PIAS and PTP. These negative regulators are responsible for maintaining signal transmission equilibrium, and any imbalance among them can exacerbate tumor metastasis.^79^ The Wnt/β-catenin pathway plays a crucial role in the maintenance of cellular polarity, and aberrant activation thereof can result in tumor metastasis.^80,81^ This pathway also serves as a pivotal regulator for preserving stem cell identity and self-renewal, thereby exerting significant influence on tumor metastasis.^82,83^

Relative to other signaling pathways, the TGF-β/SMAD and cGAS–STING pathways have been the subject of more in-depth discussions due to their dual roles in the process of tumor metastasis. These pathways exhibit distinct activities at various stages of tumor progression, which contribute significantly to the complexity of metastasis. On one hand, the TGF-β signaling pathway inhibits tumor metastasis through various mechanisms during the early stages of tumor development, including inhibiting cell proliferation and promoting apoptosis.^84^ On the other hand, in the late stages of tumorigenesis, it primarily promotes tumor invasion and metastasis, with TGF-β activating the SMAD2/3 signaling pathway, promoting EMT in tumor cells, and enhancing their invasive and metastatic capabilities.^85^ Additionally, the TGF-β signaling pathway is involved in regulating the tumor microenvironment, affecting the interactions between tumor cells and surrounding stromal cells, thereby promoting tumor metastasis. Narayan et al.^86^ found that TGF-β induces the activation of fibroblasts in the tumor microenvironment in a SMAD3-dependent manner, thereby promoting tumor invasion and metastasis. The cGAS–STING signaling pathway also has a dual role in tumor metastasis. Liu et al.^87^ mentioned that during the early stages of dormancy in metastasis, STING activity is low, aiding in immune evasion of dormant cancer cells. Zhu et al.^88^ mentioned that during the early stages of dormancy in metastasis, STING activity is low, aiding in immune evasion of dormant cancer cells. However, when cancer cells transition from the dormant phase to the proliferative phase, STING activity increases, making cancer cells more susceptible to immune system attacks, and prolonged cGAS–STING activation promotes tumor metastasis.^89,90^

### Signaling pathway: network interactions

Signaling pathways in tumor metastasis do not operate in isolation but are interwoven and influence each other. The occurrence of even minor alterations can trigger a subsequent chain reaction, ultimately impacting the process of tumor metastasis. Wu et al. elucidated that the Wnt signaling pathway can crosstalk with other signaling pathways (such as Notch, EGFR, etc.), and this network interaction significantly impacts tumor metastasis.^91^ Vashi et al.^92^ found that the persistent activation of STING induces the activation of the non-canonical NF-κB signaling pathway, promoting immune evasion and tumor metastasis. The MAPK signaling pathway is also involved in multiple signal transductions that promote tumor invasion and metastasis.^93,94^ Liu et al.^95^ showed that the activation of the Wnt/β-catenin signaling pathway can promote the activation of the NF-κB, PI3K/Akt and Hedgehog signaling pathway in tumor cells, thereby further enhancing the invasiveness and metastatic potential of tumor cells. The JAK/STAT pathway, through its transcription factors such as STAT proteins, can interact with the Wnt/β-catenin pathway at multiple levels. For instance, activated STAT proteins may bind to β-catenin or other components of the Wnt signaling cascade, influencing their activity and stability. Conversely, components of the Wnt/β-catenin pathway, including β-catenin itself, can affect the expression and activation of JAK/STAT pathway members. This reciprocal modulation can lead to the enhancement or suppression of signaling events downstream of both pathways, thereby impacting tumor metastasis.^96,97^ The Wnt ligands can activate the EGFR signaling pathway by binding to its transmembrane receptor Frizzled, which possesses seven-fold transmembrane domains. Additionally, the activation of β-catenin can be facilitated by EGFR through the receptor tyrosine kinase-PI3K/Akt pathway. Interestingly, a complex formation between EGFR and β-catenin enhances cancer cell invasion and metastasis. The balance of these intricate interactions, once disrupted, can have widespread butterfly effects. We hope to utilize multi-omics combined with artificial intelligence analysis to understand these dynamic relationships, and maintaining this balance is an important theme for tumor metastasis.

Signaling pathways play a central role in tumor metastasis, and their complexity and interconnectivity demand further in-depth research. Understanding the mechanisms of these signaling pathways aids in developing new combination therapy strategies, providing more precise guidance for personalized treatment.

### The tumor microenvironment influences cancer metastasis

**Fig. 4:**
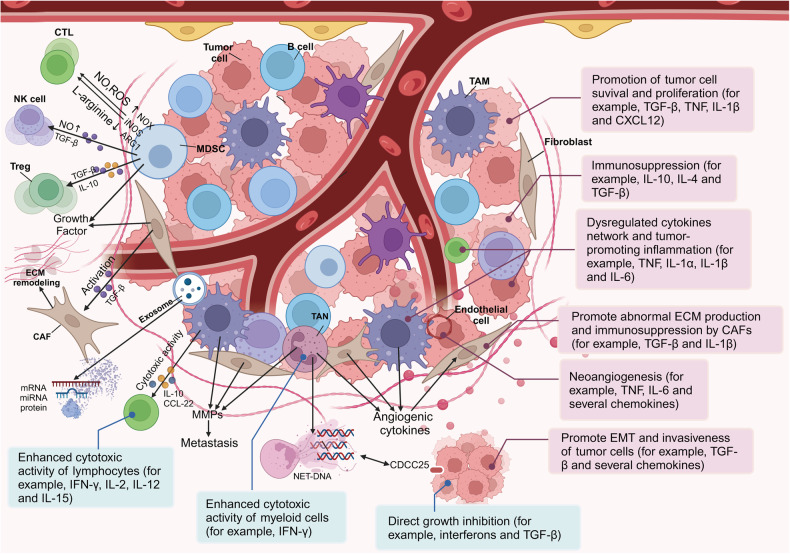
Diagram of the tumor microenvironment showing representative cell types, signaling factors, and various cytokines, as well as their mechanisms of action. At the tumor site, immune cells typically obtain tumor-related immunosuppressive phenotypes, except for cytotoxic CD8^+^lymphocytes (CTLs) that kill cancer cells. Myeloid-Derived Suppressor Cells (MDSCs) suppress immunity through various mechanisms. MDSCs can also induce T regulatory (Treg) cells, inhibit natural killer (NK) cells, and promote Tumor-Associated Macrophages (TAMs) with type 2 phenotype. Fibroblasts become Cancer-Associated Fibroblasts (CAFs), promoting extracellular matrix (ECM) remodeling. The extracellular vesicles released by various cells contain proteins, mRNA, and microRNAs that affect the microenvironment. Fibrotic cytokines recruit immune cells. Neutrophil Extracellular Traps (NETs) released by neutrophils after being stimulated can capture cancer cells. TAMs, TANs, and CAFs release angiogenic cytokines and Matrix Metalloproteinases (MMPs), promoting angiogenesis and ECM degradation, and promoting potential metastasis. In addition, the effects of cytokines are represented by red (tumor-promoting) and blue (anti-tumor) rectangles. Some cytokines, such as transforming growth factor- β (TGF-β), can have a dual effect, serving as both an anti-tumor factor and a tumor promoting factor depending on the situation. Created with BioRender.com

TME is a complex milieu encompassing a heterogeneous set of cancer cells and multiple resident and infiltrative host cells, along with various secreted factors and extracellular matrix proteins. The dynamic relationship between the tumor and its environment is both symbiotic and adversarial. Elements of the TME can profoundly influence a tumor’s capacity to initiate, proliferate, metastasize, and respond to therapeutic interventions. Tumor-secreted factors mobilize bone marrow-derived cells and immunosuppressive cells to specific sites, collaborating with resident stromal cells. This synergistic interaction reshapes the local microenvironment, fostering conditions conducive for tumor colonization and facilitating the onset of metastasis. Gaining a comprehensive understanding of the TME is pivotal, not only for deciphering tumor initiation, progression, and metastasis but also for advancing diagnostic, preventive, and prognostic measures (Fig. 4). More recently, the development of single-cell RNA sequencing (scRNA-seq) has provided richer data on the details of TME in metastatic cancer.^98^ In this chapter, we listed in detail the important literature on the effect of TME on tumor metastasis in the last five years, as shown in Table 1.

**Table 1:** List of literature on tumor microenvironment and cancer metastasis

Cell types	Key targets	Molecular mechanisms	Reference number
Fibroblasts	HSPC111	Cancer-derived exosomal HSPC111 promoted CRC liver metastasis by reprogramming lipid metabolism in CAFs.	^107^
	miR-500a-5p	Exosomal miR-500a-5p derived from CAF promoted breast cancer cell proliferation and metastasis through targeting USP28.	^109^
	CCL5	CAF-derived CCL5 inhibited the ubiquitination and degradation of HIF1α by binding to specific receptors and maintained HIF1α under normoxia, and ultimately validated its ability to promote HCC lung metastasis.	^106^
	DDR1	Matrix-metalloprotease-cleaved Col I (cCol I) and intact Col I (iCol I) exerted opposing effects on PDAC bioenergetics, macropinocytosis, tumor growth and metastasis.	^101^
	-	Distinct fibroblast profiles were observed in primary and liver metastatic tumors. Fibroblasts enriched in primary tumors contributed to worse overall survival by expressing protumor factors.	^373^
	ETV4	FGF19/FGFR4-mediated elevation of ETV4 facilitated HCC metastasis by upregulating PD-L1 and CCL2.	^374^
	TGFβ	Lack of PD-L1 blockade response was associated with a signature of TGFβ signaling in fibroblasts.	^338^
	miR-222	MiR-222 was upregulated in CAFs compared to NFs. Reprogrammed CAF enhance growth and metastasis of breast cancer.	^102^
	Tg2	Weakly migrating cells release tissue transglutaminase 2 (Tg2)-rich microvesicles that activate mouse fibroblasts, leading to migration of weakly migrating cancer cells in vitro.	^375^
	Integrin α2β1	Extracellular vesicles participated in the pre-metastatic niche formation in the lung.	^108^
	miR-21	AC1MMYR2, a small-molecule inhibitor of miR-21, attenuated NF-кB activity by directly targeting VHL. AC1MMYR2 has the ability to re-program CAF via the NF-кB/miR-21/VHL axis to inhibit lung metastasis.	^104^
	FAK	FAK signaling in CAF promoted breast cancer cell migration and metastasis through miRNAs-mediated intercellular communication.	^112^
	ITGBL1	CRC-derived ITGBL1-rich extracellular vesicles stimulated the TNFAIP3-mediated NF-κB signaling pathway to activate fibroblasts and produce high levels of pro-inflammatory cytokines to promote metastatic cancer growth.	^111^
	Peptide nano-blanket	Peptide nano-blanket impeded fibroblasts activation and subsequent formation of pre-metastatic niche.	^113^
	GATA3	GATA3 suppressed human fibroblasts-induced metastasis of clear cell renal cell carcinoma via an anti-IL6/STAT3 mechanism.	^105^
	JPJDR	JPJDR reduced CRC liver metastasis by modulating ITGBL1-rich extracellular vesicles secretion from CRC and blocked fibroblast activation by modulating ITGBL1-TNFAIP3-NF-κB signaling.	^376^
	-	Peri-tumor CAF promoted intrahepatic metastasis of HCC by recruiting CTC and maintaining their stemness characteristic.	^377^
	IL-1α/ IL-1β	IL-1α and IL-1β secreted by breast cancer cells induce lung fibroblasts to produce CXCL9 and CXCL10 via NF-κB signaling, contributing to the growth of lung metastases.	^378^
	miR-18b	CAF-derived exosomal miR-18b promoted breast cancer invasion and metastasis by regulating TCEAL7.	^110^
	miR-200	MiR-200 deficiency promoted lung cancer metastasis by activating Notch signaling in CAF.	^103^
	COX-2	COX-2-expressing lung fibroblasts remodeled the lung immune microenvironment through the production of PGE2, which leads to dendritic cell dysfunction and enhanced suppressive monocyte activity.	^100^
Endothelial cells	ADAM17	Metalloproteinase ADAM17 on endothelial cell membranes mediated shedding of the TNFR1 ecto-structural domain and subsequent processing of the γ-secretase complex which in turn promotes TNFR1-induced lung metastasis of tumor cells.	^118^
	TAK1	Deletion of TAK1 in endothelial cells increased RIPK3 expression, and overexpression of RIPK3 led to necrotic death of endothelial cells, promoting tumor extravasation and metastasis.	^114^
	PTPRO/ ASCL2	Single-cell sequencing and spatial transcriptomics revealed that stem-like cells with high expression of PTPRO and ASCL2 interact with CAF and endothelial cells through the DLL4-NOTCH signaling pathway, thereby contributing to the metastasis of primary CRC to the ovary.	^69^
	MMP 14	In melanoma, MMP 14 of the zinc-dependent endopeptidase family regulated nitric oxide production via endothelial nitric oxide synthase in endothelial cells, controlling tumor vascular function and increasing vascular instability and thus melanoma metastatic potential.	^119^
	miR-25-3p	Exosome miR-25-3p regulated the expression of VEGFR2, ZO-1, occludin and Claudin5 in endothelial cells by targeting KLF2 and KLF4, thereby enhancing CRC metastasis in the liver and lung	^379^
	ARHGEF37	Overexpression of ARHGEF37 enhanced the extravasation and metastasis of HCC cells by promoting tumor cell adhesion to endothelial cells and transendothelial migration.	^115^
	CXCL1	Lymphatic endothelial cell-secreted CXCL1 stimulates lymphangiogenesis and metastasis of gastric cancer.	^121^
	QKI	The endothelial cell target of miR-200b, RNA-binding protein QKI, promoted tumor metastasis in lung cancer by stabilizing CCND1 mRNA to facilitate endothelial cell G1/S cell cycle transition and proliferation.	^380^
	RIPK1	Treatment of mice with the RIPK1 inhibitor necrostatin-1 or endothelial cell-specific knockout of RIPK3 reduced tumor cell-induced programmed endothelial necrosis, tumor cell extravasation and metastasis.	^116^
	IFN-γ	Killing of lymphatic endothelial cells was inhibited when it lacked IFN-γ receptor expression, suggesting that IFN-γ is essential in reducing tumor-associated lymphatic vessel density and drainage and their risk of metastasis is uncontrollably elevated.	^122^
	CD146	Galectin-3 on the endothelial cell surface bound to N-linked glycans on CD146, inducing CD146 dimerization and subsequent activation of AKT signaling, promoting cytokine secretion and tumor metastasis.	^381^
	HIF-1α/HIF-2α	HIF-1α deficiency in endothelial cells reduces NO synthesis, impedes tumor cell migration through the endothelium and limits tumor cell metastasis, whereas HIF-2α deficiency has the opposite effect in each case.	^120^
	NRP-2	NRP-2 interacts with α5 integrin on endothelial cells to mediate vascular extravasation and metastasis in zebrafish and mouse xenograft models of renal clear cell carcinoma and pancreatic cancer.	^117^
	Endostatin	Treatment of primary mouse lymphoendothelial cells (mLECs) with endostatin inhibited migration, tubule formation, and activation of the Erk pathway in mLECs, Endostatin markedly inhibited tumor-associated lymphangiogenesis, resulting in decreased tumor metastasis in the lymphatic pathway.	^382^
	Nox4	Nox4 may increase tumor lymphangiogenesis through ROS/ERK/CCL21 pathway and attract CCR7-positive breast cancer cells to lymphatic vessels and distant organs.	^123^
	FGFR-1	FGFR-1 expressed in lymphatic endothelial cells was a key receptor mediating FGF-2-induced lymphangiogenesis.The interaction between FGF-2 and VEGF-C synergistically stimulated tumor growth, angiogenesis, intratumoral lymphangiogenesis, and metastasis.	^383^
	RTK	Sunitinib was a RTK inhibitor targeting various receptors and promoted tumor metastasis across the endothelial barrier in breast cancer by inducing endothelial cell senescence, leading to endothelial junction loosening.	^124^
	Tpl2	Deletion of endothelial cell tumor progression locus 2 (Tpl2) altered tight junction claudin-5 protein expression through inhibition of JNK signaling and activation of solute degradation, leading to decreased vascular permeability and immune cell infiltration.	^384^
T cell	MHC-II	MHC-II expression of tumor cells les to Treg expansion and CD4^+^ effector T cell depletion in tumor excreting lymph nodes.	^385^
	ADQ	Clinical formula Aiduqing (ADQ) significantly inhibited CXCL1 expression and secretion from TAMs, thereby inhibiting chemotaxis and differentiation of naïve CD4^+^ T cells into Tregs, inhibiting breast cancer immune escape and lung metastasis.	^130^
	anti-CD25 /CTLA4 mAb	The combination of radiotherapy and anti-CD25 /CTLA-4 mAb resulted in increases in CD8^+^ T cells and CD8^+^/CD4^+^ ratios in both primary and secondary tumors.	^131^
	CXCL13	T cell activation, cytotoxicity, and proliferation were inhibited in lymph node metastatic tumors (LNMT) of breast cancer. CD4^+^CXCL13^+^ T cells in LNMT are more likely to differentiate into a depleted state.	^127^
	PD1	Lymph node metastasis and oral squamous cell carcinoma recurrence and metastasis were related to the increase of PD1 and glycolysis of CD4^+^ T cells.	^386^
	CD4	Mitogen-activated CD4 (+) Foxp3 (-) T cells demonstrated potent suppression of NK cell proliferation and cytotoxicity and promoted metastasis of B16 melanoma.	^387^
	P-selectin	The loss of P-selectin in T cell subsets in the tumor microenvironment inhibited the infiltration of regulatory T cells and the reduction of pro-inflammatory cytokines such as IL-4, IL-10, and TGFβ in the tumor. Inhibition of P-selectin has important therapeutic effects on breast cancer growth and metastasis.	^388^
	Leucosin	For triple-negative breast cancer (TNBC), leucosin inhibited TNBC lung metastasis by improving local anti-tumor immunity. The enhancement of CD8^+^ T cell activity and Th1 immune response might be related to the down-regulation of PD-1 expression on lung CD8^+^ T cells and CD4^+^ T cells.	^389^
	DMF	Dimethyl fumarate (DMF) induced cell death in primary patient-derived CD4(+) cells and cutaneous T-cell lymphoma cell lines through the NF-KB pathway, reducing undesirable T cell infiltration and tumor metastasis	^390^
	γδT	Depletion of γδT cells or neutrophils reduced lung and lymph node metastasis without affecting primary tumor progression.	^391^
	MGP	MGP promoted the exhaustion of CD8^+^ T cells by activating the NF-κB pathway, which leads to liver metastasis of CRC.	^392^
	IL-33	IL-33 stimulated NF-κB signaling and promoted the proliferation, activation, and infiltration of CD8^+^ T cells and NK cells, thereby inhibiting lung metastasis in B16 melanoma and LLC mouse models.	^393^
	Entolimod	As a single agent in murine models of metastatic cancer in the colon and breast, entolimod induced CXCL9 and -10, supporting the localization of hematogenous CXCR3 expression by NK cells to the liver primarily by an IFN-γ signaling independent mechanism.	^394^
	BCM	When CT26 mouse colon cancer cells were inoculated into the cecum of congenous BALB/c mice and treated with both β-Casomorphin-7 (BCM) and the CD10 inhibitor thiorphan (TOP), CD8^+^ T cell infiltration was promoted and tumor growth and liver metastasis were inhibited.	^395^
	anti-PD1 and anti-CTLA4	Neoadjuvant ICB(anti-PD1 and anti-CTLA-4) therapy for breast cancer could be enhanced by simultaneous targeting of Tregs, expanding metastasis-related survival, independent of primary tumor response.	^396^
	PD1-IL2v	Using PD1-IL2v in an orthotopic PDAC KPC-driven tumor model, a clear improvement was shown in local and metastatic survival accompanied by a profound increase in a subset of tumor-infiltrating CD8^+^ T cells.	^397^
	CCL5	CCL5 affected the ratio of Treg/CD4^+^CCR5^+^ cells in breast cancer patients through CCR5, thereby affecting the metastasis and prognosis of breast cancer.	^398^
	ASO	The combination of STAT3 antisense oligonucleotide (ASO) and radiation elicited systemic immune activation and conferred a survival advantage in orthotopic and metastatic tumor models.	^133^
	anti-PD1	The addition of Treg and CD11b^+^ monocyte targeting agents in combination with anti-PD1 was a choice for cancer patients with liver metastases.	^132^
	MUC1	MUC1 interacted with EGFR to activate the EGFR/PI3K/Akt signaling pathway, induce the accumulation of Foxp3^+^ Treg cells, enhance the malignant phenotype of cholangiocarcinoma cells and tumor plasma in vivo, and ultimately enhance the growth and metastasis of cholangiocarcinoma.	^399^
	PD1-IL2v	The use of radiation therapy in combination with PD1-IL2v has been shown to improve local tumor control and survival and control metastatic spread in an orthotopic HNSCC tumor model.	^134^
	CCR8	Tumor-derived exosomes drived pre-metastatic niche formation in lung via modulating CCL1^+^ fibroblast and CCR8^+^ Treg cell interactions.	^400^
	TGF-β	Tregs induced metastasis by increasing TGF-β and enhancing the EMT	^401^
	miR-141	MiR-141-CXCL1-CXCR2 signaling-induced Treg recruitment regulated metastases and survival of non-small cell lung cancer.	^402^
	CD4	Tregs were present in the immune infiltrate of cutaneous squamous cell carcinomas and contributed to ineffective antitumor immune responses, thereby allowing cancer development and promoting metastasis.	^403^
	CCR4	CCR4^+^ Tregs are required for lung metastasis, which use beta-galactose-binding proteins to directly kill NK cells.	^404^
	NEDD8	NEDD8 depletion partially restored CD8^+^ T and NK cell-mediated antitumor immunity by suppressing Treg immunosuppression to eliminate lung metastasis after colon cancer cell surgery.	^405^
	CHSY1	CHSY1 promoted CD8^+^ T cell exhaustion through activation of succinate metabolism pathway leading to colorectal cancer liver metastasis based on CRISPR/Cas9 screening.	^125^
	CD8	Single-cell analyses revealed distinct distributions of CD8^+^ effector memory T cells at primary and metastatic sites in ovarian cancer, implicating ascites in the remodeling of tumor ecosystems.	^126^
	CD4	Tregs were a specialized subpopulation of CD4^+^ T cells, which promoted tumor progression as an early event by regulating the host immune response and subsequently promote tumor metastasis to the lymph nodes and bone marrow.	^128^
	CD4/CD8	Significant differences between naive lymph nodes (LNs) and tumor-draining lymph nodes (TDLNs) in breast cancer were found, revealing an upregulation of angiogenic pathway genes in CD4^+^ and CD8^+^ T cells, as well as an upregulation of tumor-associated genes.	^129^
B cell	HSPA4	B cells selectively promoted lymph node metastasis by producing pathogenic IgG against the glycosylated membrane protein HSPA4 and activate the HSPA4-binding protein ITGB5 and the downstream Src/NF-κB pathway in tumor cells for CXCR4/SDF1α axis mediated tumor metastasis.	^406^
	CD19	B-1 lymphocytes increased metastatic behavior of melanoma cells through the extracellular signal-regulated kinase pathway.	^407^
	CD19	B cells can suppress diverse cell subtypes, including T cells, through the secretion of anti-inflammatory mediators, such as IL-10, and can facilitate the conversion of T cells to regulatory T cells, thus attenuating anti-tumor immune responses.	^135^
	CD19	In human skin melanoma samples, metastasis was found to be associated with reduced numbers of memory B cells.	^136^
	MALT	Interaction of MALT B cells with malignant cells was increased in tumor lung tissue and metastatic lymph nodes, while this interaction was reduced in metastatic brain tissue.	^137^
	T-cell-like B cells	T-cell-like B cells were detected in most gastric cancer samples, and their expression was highest in metastatic samples and lowest in adjacent non-tumor samples.	^138^
	CD19	An increase of CD19^+^CD24hiCD27^+^ Bregs in Malignant pleural effusion (MPE) of lung cancer compared with that in blood was observed, while the CD19^+^CD27^‒^IgD^+^ naive B cells showed a significant decrease compared with that in blood.	^140^
NK cells	IFN-γ	NKp46- and Ncr1-mediated IFN-γ production led to the increased expression of the extracellular matrix protein FN1 in the tumors, which altered primary tumor architecture and resulted in decreased metastases formation.	^408^
	STING-LNP	NK cells were activated by stimulating the STING pathway, which in turn induces PD-L1 expression on cancer cells.STING-LNP treatment overcame PD1 resistance in the B16-F10 lung metastasis model.	^142^
	NK1.1	NK cells and T cells controlled lung metastasis of melanoma cells through different mechanisms, with NK cells playing a key role in shaping T cell-mediated in situ control of seeded cancer cells in the lung.	^141^
	Ethanol	Acute ethanol intoxication resulted in marked inhibition of NK activity in vivo and a 10-fold increase in the number of MADB106 tumor metastases. Ethanol had no effect on rats selectively depleted of NK cells or when NK-insensitive tumors (C4047) were used.	^409^
	TGF-β	TGF-β contributed to metabolic dysfunction of circulating NK cells in patients with metastatic breast cancer. Blockade of TGFβ and/or GARP restores NK cell metabolism and function	^144^
	Platelets	Platelets and fibrin(ogen) increased metastatic potential by impeding NK cell-mediated elimination of tumor cells.	^410^
	IFN-γ	Intermediate monocytes induced by IFN-γ inhibited cancer metastasis by promoting NK cell activation through FOXO1 and interleukin-27.	^411^
	Anesthetics	Some anesthetics, but not others, increase susceptibility to tumor metastasis, apparently by inhibiting natural killer cell activity.	^412^
	miRNA-296-3p	MiRNA-296-3p-ICAM-1 axis promoted metastasis of prostate cancer by possible enhancing survival of NK cell-resistant circulating tumor cells.	^413^
	Progranulin	Progranulin promoted melanoma progression by inhibiting NK cell recruitment to the tumor microenvironment.	^414^
	Factor XIII	Factor XIII transglutaminase supported hematogenous tumor cell metastasis through a mechanism dependent on natural killer cell function.	^415^
	ASK1	ASK1 deletion enhanced cytokine chemoattraction of NK cells, possibly by inducing ligands for NKG2D, a key activating receptor for NK cells, leading to further recruitment of NK cells to the lung.	^146^
	Tumor cell-associated tissue factor	Tumor cell-associated tissue factor and circulating hemostatic factors cooperated to increase metastatic potential through NK cell-dependent and-independent mechanisms.	^416^
	Buprenorphine	Buprenorphine ameliorated the effect of surgery on hypothalamus-pituitary-adrenal axis,NK cell activity and metastatic colonization in rats in comparison with morphine or fentanyl treatment.	^143^
	MS4A4A	The macrophage tetraspan MS4A4A enhanced dectin-1-dependent NK cell-mediated resistance to metastasis.	^147^
	HLA-E	Blocking the HLA-E:CD94-NKG2A interaction between circulating tumor cells and NK cells enhanced NK cell-mediated tumor cell killing and prevented metastasis, with the mechanism involving platelet-derived RGS18 promoting HLA-E expression via the AKT-GSK3β-CREB pathway.	^145^
DC	CD83	Advanced-stage CRC tumors had a lower density of CD83^+^ DCs in the stroma and invasive margin.	^417^
	monocyte-like DC	During gastric cancer peritoneal metastasis progression, an increase was seen of monocyte-like DCs that were pro-angiogenic with reduced antigen-presenting capacity and correlated with poor gastric cancer prognosis.	^153^
	Nanovaccine	Delivery of neoantigen-exosome nanovaccine prohibited tumor growth, prolonged survival, delayed tumor occurrences with long-term memory, eliminated the lung metastasis in the therapeutic, prophylactic and metastatic B16F10 melanoma as well as therapeutic MC-38 models, respectively.	^418^
	-	Overcoming cDC deficiency in early-stage PDAC leads to disease restraint, while restoration of cDC function in advanced PDAC restores tumor-restraining immunity and enhances responsiveness to radiation therapy.	^419^
TANs	ETV4	The transcription factor ETV4 enhanced bladder cancer cell-derived CXCL1/8 to recruit TANs, leading to an increase in VEGFA and MMP9 in TANs, which then promoted lymphangiogenesis and LN metastasis.	^158^
	STAT3	TAN and macrophages interaction contributed to intrahepatic cholangiocarcinoma progression by activating STAT3.	^420^
	DDR1	DDR1-induced NETs drived pancreatic cancer metastasis.	^159^
	-	Immunosuppressive reprogramming of neutrophils by lung mesenchymal cells promoted breast cancer metastasis.	^421^
	AGR2	TAN secreted AGR2 to promote CRC metastasis via its receptor CD98hc-xCT.	^160^
	-	TAN extracellular traps regulated nanocarrier-enhanced inhibition of malignant tumor growth and distant metastasis.	^162^
	SIRT1	Aged neutrophils formed mitochondria-dependent vital NETs to promote breast cancer lung metastasis.	^422^
	-	Seven TAN subtypes were identified in advanced NSCLC, revealing that the N3 subtype is pro-tumorigenic, associated with poor survival, and interacts closely with macrophages via chemokine signaling within the tumor microenvironment.	^156^
	CCL4	TAN populations enriched in the myeloid-cell-enriched subtype of liver tumors were linked to a poor prognosis, with CCL4^+^ TANs recruiting macrophages and PD-L1^+^ TANs suppressing T cell cytotoxicity.	^157^
	-	Neutrophils were recruited to the site of pre-metastatic niches such as lung, liver, and omentum and that they can aide in remodeling the local microenvironment through NETs and through the secretion of the pro-inflammatory mediators that further facilitate tumor cell extravasation and proliferation.	^161^
TAMs	JAK2	TAMs induced the EMT program to enhance CRC migration, invasion and CTC-mediated metastasis by regulating the JAK2/STAT3/miR-506-3p/FoxQ1 axis.	^166^
	FGD5-AS1	Pancreatic cancer cells produced FGD5-AS1-rich ectodomains that lead to M2 macrophage polarization and promote the malignant behavior of cancer cells.	^423^
	SUCNR1	The SUCNR1-triggered PI3K- HIF-1α axis mediated the polarization of macrophages to TAM, which promotes cancer cell migration and invasion, and also promotes cancer metastasis.	^424^
	REDD1	Hyperglycolytic REDD1-deficient TAMs rendered glucose unavailable to endothelial cells, thereby preventing vascular hyperactivation and promoting the formation of quiescent vascular junctions. Downregulation of glycolysis in REDD1 knockout TAMs re-establishes aberrant angiogenesis and metastasis.	^169^
	CAP2	Polarized M2 macrophages induced pre-translocation site formation and promoted gastric cancer translocation by secreting TGFB1, resulting in a TGFB1/JUN/CAP2 positive feedback loop that successively activated CAP2 expression.	^167^
	APOC1	Systematic pan-cancer analysis identified APOC1 as an immunological biomarker which regulated macrophage polarization and promotes tumor metastasis.	^425^
	CTSK	The tumor secretagogue CTSK bound to toll receptor 4 (TLR4) and stimulate M2 polarization of TAMsvia an mTOR-dependent pathway.	^426^
	CCL5	CCL5 derived from TAMs promoted prostate cancer stem cells and metastasis via activating β-catenin/STAT3 signaling.	^427^
	Cathepsin B	Increased glucose metabolism in TAMs fueled O-GlcNAcylation of lysosomal Cathepsin B to promote cancer metastasis and chemoresistance.	^185^
	GDNF	TAM-derived GDNF promoted gastric cancer liver metastasis via a GFRA1-modulated autophagy flux.	^428^
	ALOX5	The ALOX5 inhibitor Zileuton regulated TAM M2 polarization by JAK/STAT and inhibits pancreatic cancer invasion and metastasis.	^429^
	Emodin	Emodin reduced breast cancer lung metastasis by suppressing macrophage-induced breast cancer cell EMT and CSC formation.	^430^
	Fibronectin 1	Fibronectin 1 derived from TAM and fibroblasts promoted metastasis through the JUN pathway in HCC.	^431^
	LINC00543	LINC00543 promoted CRC metastasis by driving EMT and inducing M2 polarization in TAMs.	^432^
	FoxM1	Invasive FoxM1 phosphorylated by PLK1 induced the polarization of TAMs to promote immune escape and metastasis, amplified by IFITM1.	^433^
	NOTCH1	TAMs in direct contacted with prostate cancer cells promote malignant proliferation and metastasis through NOTCH1 pathway.	^434^
	S1pr1	In CD11bhi CD206^+^ TAMs infiltrating mouse mammary tumors, genetic deletion of S1pr1alone prevented lung metastasis and tumor lymphangiogenesis.	^435^
	HO-1	A unique subset of TAMs (F4/80hiCD115hiC3aRhiCD88hi), endowed with a high rate of heme catalysis by the stress-responsive enzyme heme oxygenase-1 (HO-1), played a key role in shaping the proto-metastatic tumor microenvironment conducive to immunosuppression, angiogenesis, and EMT.	^436^
	Cx43	Inhibition of melanoma migration, invasion and metastasis by activation of the Cx43-gap junction-intercellular communication (Cx43-GJs)/IFN-γ/STAT1 pathway and inhibition of Cx43-GJs/IL-4/JAK2/STAT3 repolarized macrophages from M2 to M1 phenotypes.	^437^
	IL6	IL6-STAT3-C/EBPβ-IL6 positive feedback loop in TAM promoted the EMT and metastasis of lung adenocarcinoma.	^438^
	MRC1	A single-cell and spatial atlas of CRC liver metastasis revealed the presence of highly metabolically activated MRC1^+^CCL18^+^ M2-like macrophages in metastatic sites.	^165^
	CCL18	CCL18 from TAMs promoted breast cancer metastasis via PITPNM3.	^168^
	STING	Macrophage STING signaling promoted NK cell to suppress CRC liver metastasis via 4-1BBL/4-1BB co-stimulation.	^170^
	ANGPTL1	Exosomal ANGPTL1 attenuated CRC liver metastasis by regulating Kupffer cell secretion pattern and impeding MMP9 induced vascular leakiness.	^171^
MDSCs	SMAD3	KAT6A Acetylation of SMAD3 regulates MDSC recruitment, metastasis, and immunotherapy in TNBC.	^439^
	Galectin-1	Galectin-1 increased STING stability in cancer cells that activated NF-κB signaling and CXCL2 expression to promote MDSC trafficking, which stimulated the generation of a premetastatic niche and facilitates metastatic progression.	^176^
	CXCL1	CXCR2 knockdown and CXCR2-/- MDSCs transplantation impaired CUMS-mediated MDSC elevation, PMN formation and breast cancer metastasis.	^177^
	LILRB4	LILRB4/gp49B promoted MDSC-mediated tumor metastasis by regulating M2 polarization of MDSCs and inhibiting secretion of miR-1 family miRNAs to facilitate tumor migration and invasion.	^440^
	Sponge-like nano-system	Sponge-like nano-system suppressed tumor recurrence and metastasis by restraining MDSC-mediated immunosuppression and formation of pre-metastatic niche.	^441^
	Micellar nanoparticles	Micellar nanoparticles inhibited the postoperative inflammation, recurrence and pulmonary metastasis of 4T1 breast cancer by blocking NF-κB pathway and promoting MDSCs depletion.	^442^
	-	MDSCs not only facilitated the release of metastases from dormancy but also may enhance metastatic organ specificity by supporting tumor cell escape from the host’s adaptive immune system.	^173^
	-	Compared with primary pancreatic cancer, liver metastases were significantly enriched in granulocytic MDSCs, mature neutrophils, and myeloid progenitor cells.	^174^
	-	Myeloid-like tumor hybrid cells in bone marrow promote progression of prostate cancer bone metastasis.	^175^

### Cancer-associated fibroblasts

Fibroblasts, predominantly found in connective tissues, originate from mesenchymal cells. Recognized for their exceptional resilience under adverse conditions, these cells play a pivotal role in various tissue types, with their density varying across different connective tissues. Within oncological contexts, fibroblasts collaborate with tumor cells and other TME components, exerting either promotive or inhibitive effects on tumor growth and metastasis. In such contexts, they are specifically termed as cancer-associated fibroblasts (CAFs).^99^ Gong et al.^100^ identified a population of cyclooxygenase 2 (COX-2)-expressing adventitial fibroblasts that remodeled the lung immune microenvironment by scRNA-seq and immunofluorescence. At steady state, fibroblasts in the lungs produced prostaglandin E2 (PGE2), which drove dysfunctional dendritic cells (DCs) and suppressive monocytes. Tumor-associated inflammation, especially the pro-inflammatory cytokine interleukin-1β, sustained a pre-metastatic niche by promoting this lung-intrinsic stromal program. Additionally, deleting Ptgs2 in fibroblasts or simultaneously inhibiting the PGE2 receptors EP2 and EP4 enhanced the anti-metastatic efficacy of DC-based therapy combined with PD1 blockade.

Fibroblast-related regulatory genes perform different functions in tumor metastasis. Su et al.^101^ showed that on the bioenergetics, macropinocytosis, tumor development, and metastasis of pancreatic ductal adenocarcinoma (PDAC), matrix-metalloprotease-cleaved Col I (cCol I) and intact Col I (iCol I) exhibit oppose effects. In contrast to cCol I, which stimulates the discoidin domain receptor 1 (DDR1)–NF-κB–p62–NRF2 signaling to encourage PDAC growth, iCol I causes DDR1 to degrade and inhibits PDAC growth. It was found that miR-222 was upregulated in CAFs compared to normal fibroblasts (NFs) in breast cancer.^102^ Lamin B receptor (LBR) was identified as a direct miR-222 target and, since the LBR knockdown phenotype miR-222 overexpression and the LBR overexpression expression phenotype miR-222 knockdown, so are functionally related. miR-222 overexpression, or LBR knockdown, was sufficient to induce NFs displaying enhanced migration, invasion, and senescence characteristic of CAFs, and in addition, conditioned mediators from these fibroblasts induced an increase in breast cancer cell migration and invasion. Lung cancer cells lacking MiR-200 stimulate the growth and activation of nearby CAFs, increasing the cancer cells’ propensity to metastasize. At least in part, miR-200 targets the Notch ligands Jagged1 and Jagged2 in cancer cells and activates Notch in nearby CAFs to control the functional relationship between cancer cells and CAFs.^103^ Interestingly, Ren et al.^104^ made a preliminary transformation of the function of miRNA in CAF from cancer. According to their research, miR-21 plays a crucial role in mediating breast cancer spread by way of the tumor environment. A more individualized and potent neoadjuvant treatment for patients to decrease metastasis and enhance the chemotherapeutic response may be created using AC1MMYR2, a small molecule inhibitor of miR-21. In addition, in the normal fibroblast-conditioned media treated with renal cancer cells, GATA3 was increased in surrounding normal tissues but downregulated at both the mRNA and protein levels. GATA3 overexpression reduced the migration of both IL6-stimulated and renal carcinoma cells, and it markedly decreased STAT3 phosphorylation.^105^ Xu et al.^106^ demonstrated that by attaching to particular receptors, CAF-derived CCL5 prevented the ubiquitination and degradation of hypoxia-inducible factor 1 alpha (HIF1α), preserved HIF1α under normoxia, upregulated the downstream gene zinc finger enhancer-binding protein 1 (ZEB1), and induced EMT, thereby confirming its capacity to encourage HCC lung metastasis. Therefore, the interaction between cancer cells and CAFs is an important mechanism that promotes metastasis potential. How to balance gene expression in CAF is the most direct therapeutic strategy to inhibit tumor metastasis.

Some literature reports that extracellular vesicles (EVs) play a key role in CAF’s interaction with cancer cells, thereby promoting tumor metastasis. Zhang et al.^107^ identified CRC cell-derived exosomal HSPC111 facilitated pre-metastatic niche formation and CRC liver metastases (CRLM) by reprogramming lipid metabolism. CRC patients with liver metastasis had higher level of HSPC111 in serum exosomes, primary tumors and CAFs in liver metastasis than those without. Kong et al.^108^ demonstrated that periostin is a possible biomarker that might be used to identify the pre-metastatic niche that CAF-EVs created in the lung. CAF-EVs were involved in the establishment of this niche and shown a high degree of aptitude for matrix remodeling. Chen et al.^109^ confirmed that CAF-derived exosomal miR-500a-5p significantly promoted the proliferation and metastasis of breast cancer cells through targeting USP28. In addition, CAF-derived exosomal miR-18b also promoted breast cancer invasion and metastasis by regulating TCEAL7.^110^ In a CRC model, primary tumors stimulate local fibroblasts in distant organs by releasing EVs rich in integrin beta-like 1 (ITGBL1) into the bloodstream. Activated fibroblasts secrete pro-inflammatory cytokines, such IL-6 and IL-8, which cause the creation of pre-metastatic niches and accelerate the spread of metastatic cancer. Mechanistically, TNFAIP3-mediated NF-κB signaling pathway is stimulated by primary CRC-derived ITGBL1-enriched EVs, activating fibroblasts.^111^ Hsin-Jung Wu et al.^112^ identified that through intercellular communication mediated by exosomal miRNAs, focal adhesion kinase (FAK) activation in CAFs facilitated breast cancer cell migration and metastasis. These findings reveal cancer cell-CAF interactions in the metastatic tumor microenvironment, as well as close signaling exchange between primary tumors and metastases via EVs. Targeting EVs offers an attractive therapeutic approach for tumor metastasis. In addition, it is important to note that Zhou et al.^113^ have revealed the existence of an enzyme-activated assembled peptide, FR17, which may precisely put out the “fire” of tumor-supportive microenvironment adaptation by acting as a “flame-retarding blanket” in the pre-metastatic niche. It was demonstrated that the in-situ formed peptide nano-blanket suppresses the remodeling of the host stromal tissue that supports metastases, inhibits the activation of fibroblasts, and reverses angiogenesis and vascular instability. All the above literatures have demonstrated that proper regulation of CAF is expected to inhibit tumor metastasis.

### Endothelial cells

Endothelial cells, a type of phagocytes, are ubiquitously present in organs and tissues such as the brain, lymph node, lung, liver, and spleen, and actively contribute to collective immunization processes. Due to their pivotal roles in vascular biology, encompassing coagulation, arteriosclerosis, angiogenesis, inflammation, and edema, endothelial cells directly facilitate metastatic progression. Single-cell sequencing and spatial transcriptomics revealed that stem-like cells with high expression of PTPRO and ASCL2 interact with CAFs and endothelial cells through the DLL4-NOTCH signaling pathway, thereby contributing to the metastasis of primary CRC to the ovary.^69^ Deletion of TGF-β-activated kinase 1 (TAK1) in endothelial cells increases RIPK3 expression, and overexpression of RIPK3 leads to necrotic death of endothelial cells in animal models, promoting tumor extravasation and metastasis.^114^ In HCC, overexpression of Rho guanine nucleotide exchange factor 37 (ARHGEF37) significantly enhanced the extravasation and metastasis of HCC cells by promoting tumor cell adhesion to endothelial cells and transendothelial migration. In terms of the specific mechanism, ARHGEF37 directly interacts with and activates Cdc42 to promote the formation of invasive pseudopods within HCC cells, thereby disrupting the interaction between endothelial cells and pericytes. Importantly, ZCL278, a specific inhibitor of Cdc42, significantly inhibited the adhesion of HCC cells overexpressing arhgef37 to endothelial cells, as well as adhesion and extravasation in alveoli, thereby inhibiting HCC lung metastasis in animal models.^115^ Boris Strilic et al.^116^ showed that tumor cells from humans and mice cause endothelial cells to undergo programmed necrosis, or necroptosis, which encourages tumor cell extravasation and metastasis. Mice treated with either endothelial cell-specific RIPK3 deletion or receptor-interacting serine/threonine-protein kinase 1 (RIPK1)-inhibitor necrostatin-1 showed a reduction in tumor-cell-induced endothelial necroptosis, tumor cell extravasation, and metastasis. In the contrast, these activities were enhanced by pharmacological caspase inhibition or deletion of caspase-8 specific to endothelial cells. In zebrafish and murine xenograft models of clear cell renal cell carcinoma (RCC) and pancreatic adenocarcinoma, neuropilin-2 (NRP-2), a multifunctional nonkinase receptor for semaphorins, vascular endothelial growth factor (VEGF), and other growth factors, expressed on cancer cells, interacts with α5 integrin on endothelial cells to mediate vascular extravasation and metastasis.^117^ Metalloproteinase ADAM17 on endothelial cell membranes mediates shedding of the TNFR1 ecto-structural domain and subsequent processing of the γ-secretase complex which in turn promotes TNFR1-induced lung metastasis of tumor cells, thus long-term cancer metastasis development in the lung is prevented by both short-term pharmacological suppression of ADAM17 and genetic deletion of ADAM17 in endothelial cells.^118^

Most of the studies that promote cancer metastasis focus on vascular endothelial cells and lymphatic endothelial cells.In melanoma, matrix metalloproteinase (MMP) 14 of the zinc-dependent endopeptidase family regulates nitric oxide (NO) production via endothelial NO synthase in endothelial cells, controlling tumor vascular function and increasing vascular instability and thus melanoma metastatic potential.^119^ In endothelial cells, HIF-1α shortage inhibits NO production, blocks tumor cell migration across the endothelium, and restricts tumor cell metastasis; in these scenarios, HIF-2α deficiency has the opposite effect. This results from varying NO homeostasis regulation, which in turn controls the expression of vascular endothelial growth factor in a NO-dependent feedback loop.^120^ In a gastric cancer tumor co-culture system, gastric cancer cells induced lymphatic vessel endothelial cells (LECs) to secrete CXCL1 via the NF-κB pathway. LEC migration and tube formation involving FAK-ERK1/2-RhoA activation and F-actin recombination were subsequently triggered by CXCL1, which in turn led to the end result of metastasis of gastric cancer tumors. In human gastric cancer specimens, CXCL1 receptor CXCR2 expression was positively correlated with TNM (tumor, lymph node, metastasis) stage and lymphatic vessel density. This implies that LECs are crucial to the process of gastric cancer metastasis.^121^ Tumor cell death mediated by T cells triggers the release of tumor antigens and the cross-presentation of tumor-associated LECs, which enables T cells to kill LECs that are specific to a certain antigen. Killing of LECs was inhibited when LECs lacked IFN-γ receptor expression, suggesting that IFN-γ is essential in reducing tumor-associated lymphatic vessel density and drainage. Once the density of tumor lymphatic vessels rises, their risk of metastasis is uncontrollably elevated.^122^ Elevated levels of NADPH oxidase 4 (Nox4) in the endothelial cells of breast tumors correlate with the lymph node metastasis in patients. In a 3D setting, Nox4 enhances both tube formation and lymphatic sprouting. Studies in vivo demonstrate that blocking Nox4 in 4T1 tumor-bearing mice significantly diminishes lymphangiogenesis and metastatic spread associated with the tumor. It is suggested that Nox4 facilitates tumor lymphangiogenesis via the ROS/ERK/CCL21 pathway, thereby drawing CCR7-positive breast cancer cells towards lymphatic channels and distant anatomical sites.^123^ Sunitinib is a receptor tyrosine kinase (RTK) inhibitor that targets many receptors, including vascular endothelial growth factor receptors (VEGFRs). It induces senescence in endothelial cells, which in turn facilitates tumor dissemination across the endothelial barrier in breast cancer, leading to endothelial junction loosening. Ironically, sunitinib may have been “correctly” acting in its intended purpose of targeting endothelium cells, as seen by the increased rate of tumor metastasis.^124^ These results imply that, before utilizing sunitinib and other antiangiogenic medications that target endothelial cells directly, we should carefully consider the advantages and disadvantages.

### T cells

T cells, derived from lymphoid stem cells within the thymus, are a prominent and intricate subset of lymphocytes. Their anti-metastatic action is predominantly mediated by cellular cytotoxicity. Regarding the relationship between tumor metastasis and CD8^+^ T cells, the current research mainly focuses on the exhaustion of CD8^+^ T cells. For example, our previous study has shown that MGP stimulates CD8^+^ T cell exhaustion by triggering the NF-κB pathway, which results in CRC liver metastases.^125^ In addition, based on CRISPR/Cas9 screening, CHSY1 induces CD8^+^ T cell fatigue by activating the succinate metabolism pathway, which results in CRC liver metastases.^125^ Xiaocui Zheng et al.^126^ presented a single-cell landscape of the ovarian cancer (OC) ecosystem, highlighting five tumor-relevant areas such as malignant ascites and omentum metastases. The results indicated that HAVCR2^+^ exhausted CD8^+^ cells were mostly abundant in tumor sites. Two CD8^+^ effector memory (TEM) clusters occupied a large proportion of CD8^+^ T cells, with ANXA2^+^ TEM enriched in tumor sites and GZMK^+^ TEM enriched in ascites. Thus, it is advantageous to reverse CD8^+^ T cell exhaustion in order to stop tumor spread.

When it comes to immune responses against infections and cancer cells, CD4^+^ T cells are crucial. Tong Liu et al.^127^ demonstrated that compared to primary tumors (PT), lymph node metastasized tumors (LNMT) exhibit reduced T cell activation, cytotoxicity, and proliferation. There is a higher likelihood of CD4^+^CXCL13^+^ T cells in LNMT differentiating into an exhausted condition. Tregs are a distinct subset of CD4^+^ T cells that facilitate early tumor growth by modulating the host immune response and subsequently promote tumor dissemination to the bone marrow and lymph nodes.^128^ Li Yen-Liang et al.^129^ revealed that there are a number of noteworthy differences between breast cancer patients’ naïve lymph nodes (LNs) and tumor-draining lymph nodes (TDLNs) and found that CD4^+^ and CD8^+^ T cells showed upregulated angiogenesis pathway genes and higher treg-associated genes. Jing Li et al.^130^ reported that clinical formula Aiduqing (ADQ) significantly inhibited CXCL1 expression and secretion from TAMs, thereby inhibiting chemotaxis and differentiation of naive CD4^+^ T cells into tregs, thereby enhancing the cytotoxic effect of CD8^+^ T cells. This research not only offers preclinical evidence that supports the use of ADQ to inhibit lung metastasis in breast cancer but also reveals new perspectives on the TAM/CXCL1/NF-κB/FOXP3 signaling pathway as a viable target for Treg modulation and the immunotherapy of breast cancer. Moreover, the interactions between CD4^+^ T cells and various cells are crucial in tumor metastasis and represent a strategic approach for intervention in such processes.

The literature on the effects of drugs or physical therapy on T cells in patients with tumor metastasis is also of great interest to us. Dengbo Ji et al.^131^ demonstrated that integrating radiotherapy with anti-CD25/CTLA4 mAb therapy significantly augmented the count of CD8^+^ T cells and the CD8^+^/CD4^+^ ratio in both primary and secondary tumors, in comparison to radiotherapy alone. This multifaceted approach not only reduced levels of Tregs, PD1^+^CD8^+^, and PD1^+^CD4^+^ T cells but also curtailed growth in both locally irradiated and distal unirradiated tumors, while enhancing overall survival rates. Furthermore, combining radiotherapy with anti-CTLA4 specifically lessened liver metastasis. The research conducted by James C. Lee et al.^132^ indicates that the immune reaction triggered by tumor antigens in the liver results in a global suppression of antitumor immune responses in a dual-tumor immunocompetent mouse model. This suppression is specifically linked to tumor antigens and involves the synchronous activation of tregs and the alteration of CD11b^+^ monocytes within the tumor. The impaired immune condition could not be remedied through anti-PD1 monotherapy alone, unless regulatory T cells were either depleted using anti-CTLA-4 therapy or destabilized with an EZH2 inhibitor. Consequently, this investigation underscores the need to integrate agents targeting Treg and CD11b^+^ monocytes with anti-PD1 therapy for treating cancer patients with liver metastases. Miles Piper et al.^133^ demonstrated that while radiotherapy enhances the infiltration and activation of DCs, it also augments Treg infiltration, without recruiting NK and CD8^+^ T cells in pancreatic ductal adenocarcinoma (PDAC) patient tissues. Inhibiting STAT3 in Tregs notably improves the control of both local and distant disease progression and boosts NK cell-mediated surveillance of metastases. Additionally, combining STAT3 antisense oligonucleotide (ASO) with radiotherapy stimulates systemic immune responses and provides a survival benefit in models of both orthotopic and metastatic tumors. Interleukin-2 (IL-2) regulates lymphocyte survival and function and presents as a promising immunotherapy target, albeit its efficacy is curtailed by Tregs that express high-affinity IL-2Rα. Jacob Gadwa et al.^134^ observed that the bispecific immunocytokine PD1-IL2v effectively targets PD1-expressing cells, channeling IL-2 signaling predominantly through the intermediate-affinity receptor IL-2Rβγ. This selective engagement facilitates the induction of anti-tumor responses in effector T cells and NK cells, while simultaneously curtailing the suppressive effects of IL-2Rα activation on Tregs. Additionally, combining this immunocytokine with radiation therapy (RT) enhances local tumor control, boosts survival rates, and mitigates metastatic progression in orthotopic models of head and neck squamous cell carcinoma (HNSCC). Consequently, integrating multi-target drugs with physical therapies, including radiotherapy, holds promise for addressing tumor metastasis.

### B cells

B cells go through a variety of phenotypes as they mature and differentiate. Naive B cells, Mz-B cells, Fo-B cells and memory B cells enter the peripheral blood and tissue sites. These B cells express MHCII, CD80, CD86, CD69, and other surface markers, which help T-cell activities and play a part in antigen presentation. They are also capable of secreting cytokines including IFN, IL-2, and IL-4.^135^ The regulation of B cells in the TME is also complex. Minyi Chen et al.^136^ analyzed the prevalence and spatial arrangement of six key subpopulations of B cells and antibody-secreting cells outside tertiary lymphoid structures across 154 cutaneous melanoma samples (53 primary tumors without subsequent metastasis, 44 primary tumors with metastasis, 57 metastatic samples) and observed a correlation between metastasis and a reduction in memory-like B cell numbers. Moreover, older patients displayed elevated counts of plasmablast-like cells and a significantly greater presence of plasma cell-like cells at distant metastatic sites. Additionally, a higher concentration of memory-like B cells was noted at locoregional compared to distant metastatic sites. Xiaoyuan Wang et al.^137^ characterized three subtypes of B cells: follicular B, MALT B, and plasma cells, through clustering and detailed annotation. They observed increased interactions between MALT B cells and malignant cells in both tumor lung tissue (tLung) and metastatic lymph nodes (mLN). However, these interactions were reduced in the metastatic brain (mBrain) group. This difference indicated that metastatic tumors in the mBrain group underwent more significant transcriptomic changes compared to those in the mLN group and the primary tLung tumors. Haiping Jiang et al.^138^ analyzed 7708 B cells, organizing them into five distinct lineages based on marker gene expression. They identified a unique subset of B cells that expressed typical B lymphocyte surface markers (CD79A, MS4A1 (CD20), CD19, CD40) along with the T lymphocyte marker CD3D. Termed T cell-like B cells, these were predominantly found in gastric cancer samples, with the highest prevalence in metastatic (M) and the lowest in adjacent non-tumoral (NT) samples.

B cells exhibit distinct cell surface marks, secrete distinct chemicals, and perform distinct functions as a result of being regulated by a range of immune cells, tumor cells, cytokines, and chemokines in the surrounding immunological milieu during their growth and differentiation. Thus, a multitude of studies have demonstrated that B cells facilitate the growth of tumors. Typically, these B cells are categorized as regulatory B cells, or bregs.^139^ Ming-Ming Shao et al.^140^ observed an increase of CD19^+^CD24hiCD27^+^ Bregs in Malignant pleural effusion (MPE) of lung cancer compared with that in blood, while the CD19^+^CD27^‒^IgD^+^ naive B cells showed a significant decrease compared with that in blood. Bregs from MPE simultaneously boosted Treg development, reduced Th1 cell differentiation, and had no impact on Th17 cells in vitro. These findings suggested that Bregs may have a significant regulatory function in the MPE tumor microenvironment. Therefore, based on the functional diversity of B cells, their functions and mechanisms in tumor metastasis need to be further elucidated.

### NK cells

NK cells are instrumental in controlling tumor metastasis, and further insights are anticipated into how they mediate the clearance of metastatic tumors. Maulik Vyas et al.^141^ observed that NK cells significantly curbed the pulmonary seeding of B16 melanoma cells and T cells primarily restricted the growth of metastatic foci in the lungs. Despite the absence of NK cells in the lung’s metastatic foci at the study’s end, their critical role in facilitating T cell recruitment to these sites was evident. Distinct mechanisms are employed by NK and T cells in managing pulmonary metastasis of melanoma, with NK cells crucially influencing the in situ T cell-mediated control of lung-seeded cancer cells. A detailed understanding of NK and T cells’ collaborative impact on tumor metastasis is vital for developing advanced cancer immunotherapies.

Numerous studies are examining methods to enhance NK cell functions to hinder tumor metastasis. Resistance to ICI poses a significant challenge in cancer immunotherapy. Takashi Nakamura et al.^142^ demonstrated that treating with a lipid nanoparticle containing a stimulator of interferon genes (STING) agonist (STING-LNP) could counteract PD1 resistance in a B16-F10 lung metastasis model. This effect is due to the activation of NK cells by the STING pathway, which also induces PD-L1 expression on cancer cells. Silvia Franchi et al.^143^ studied the impact of buprenorphine in preventing experimental surgery’s effects on HPA axis activation (plasma corticosterone levels), NK activity, and lung metastasis of the NK-sensitive tumor MADB106. Unlike morphine and fentanyl, which stimulate the HPA axis, reduce NK activity, and increase tumor metastasis, buprenorphine does not have these effects. Surgery significantly elevated corticosterone levels, reduced NK activity, and heightened MADB106 metastasis. Buprenorphine alone managed to counteract these neuroendocrine and immune alterations, thereby mitigating the increase in tumor metastasis caused by surgical stress. These preclinical findings advocate for the protective role of buprenorphine against metastatic spread post-cancer surgery by treating surgically induced stress immunosuppression.

Further investigations have explored the abnormal functioning of NK cells in TME. TGF-β has been shown to impair the metabolism of circulating NK cells in metastatic breast cancer patients. Targeting TGF-β and/or GARP could rejuvenate NK cell metabolism and function, marking an essential aim for boosting NK cell-based immunotherapies.^144^ Xiaowei Liu et al.^145^ discovered that CTCs and NK cells interact via the immune checkpoint molecules HLA-E:CD94-NKG2A. Interrupting this interaction by blocking NKG2A or downregulating HLA-E expression enhances NK-mediated tumor cell eradication in vitro and prevents tumor metastasis in vivo. Studies suggest that RGS18, secreted by platelets, upregulates HLA-E expression via AKT-GSK3β-CREB signaling, and its overexpression aids pancreatic tumor metastasis to the liver. Makoto Fujimoto et al.^146^ found that ASK1 deficiency in mice enhances NK cell-mediated clearance of intravascular tumor cells during early metastasis stages. Following tumor inoculation, ASK1 deficiency led to the upregulation of genes related to the immune response, including interferon-gamma (IFN-γ), and revealed the necessity of NK cells for these anti-metastatic effects. ASK1 deficiency also increased cytokine production, attracting NK cells possibly through induction of the ligand for NKG2D, a crucial NK cell receptor, promoting further NK cell recruitment to the lungs. These findings highlight that ASK1 inhibits NK cell-dependent anti-tumor immunity, suggesting that targeting ASK1 could be a promising approach in cancer immunotherapy to counteract tumor metastasis. Additionally, MS4A4A, a tetraspan molecule selectively expressed in macrophages during differentiation and polarization, is vital for dectin-1-dependent activation of NK cell-mediated resistance to metastasis.^147^ All these studies emphasize that modulating NK cell functions can significantly contribute to effective anti-tumor metastasis strategies, pointing towards future directions for developing anti-metastatic drugs.

### Dendritic cells

DCs are professional antigen-presenting cells that are crucial for the initiation of the immune response to specific antigens through internalization of foreign antigens and subsequent presentation to T cells. To avoid immune surveillance, cancer cells may suppress DCs through multiple mechanisms, including the secretion of immunosuppressive TGF-β.^148^ In CRC, myeloid DCs, the most common DC subtype associated with cell-mediated immunity and stimulation of naïve CD4^+^ T cells to the TH1 phenotype, are found in increased frequency at the tumor invasive front and are associated with lymph node invasion.^149^ In contrast, Gulubova et al.^150^ noted that advanced-stage CRC tumors had a lower density of CD83^+^ DCs in the stroma and invasive margin.

The mechanism through which these DCs enhance invasiveness has not been well-elucidated. Orsini et al.^151^ demonstrated that DCs from CRC patients had impaired antigen-presenting capacity and reduced co-stimulatory molecule expression compared to DCs from healthy controls. Furthermore, CRC patient-derived DCs secreted increased levels of immunosuppressive IL-10 and decreased levels of immunostimulatory IL-12 and TNF-α. Concordantly, Nagorsen et al.^152^ found that while the presence of S100^+^ DCs in limited disease stages correlates with better survival rates, their increased presence in the CRC TME also coincides with greater Treg cell infiltration, underscoring a potential pro-tumorigenic role of these DCs. Xuan-Zhang Huang et al.^153^ characterize single-cell transcriptomes of 191,987 ascites cancer/immune cells from 35 patients with/without gastric cancer peritoneal metastasis (GCPM). During GCPM progression, an increase is seen of monocyte-like DCs that are pro-angiogenic with reduced antigen-presenting capacity and correlate with poor gastric cancer prognosis. They also describe the evolution of monocyte-like DCs and regulatory and proliferative T cells following therapy. Two autophagy-related genes (MARCKS and TXNIP) mark high-plasticity GC with poorer prognosis, and autophagy inhibitors induce apoptosis in patient-derived organoids.

The biggest breakthrough in DC research has been the development of vaccines. Javier Rodriguez et al.^154^ reported that DC vaccines loaded with autologous tumor lysates were tested for their potential to avoid or delay disease relapses (NCT01348256). After neoadjuvant chemotherapy and surgery, followed by adjuvant chemotherapy, fifteen patients with clean resection margins were randomized to receive either two courses of DC vaccines administered intradermally over four days or to undergo observation. Follow-up of the patients indicates a clear tendency to fewer and later relapses in the vaccine arm versus observation arm.

### Tumor-associated neutrophils

Neutrophils, originating from myeloid progenitors, undergo maturation in the bone marrow before being released into the bloodstream, where they navigate under the influence of chemokines and Integrin. In the same way as tumor-associated macrophages in the tumor microenvironment are categorized (M1 being antitumor macrophages, M2 being pro-tumor macrophages), neutrophils are also divided into two polarization states: N1 being antitumor neutrophils and N2 being pro-tumor neutrophils. Pro-tumor factors that cause immunosuppression in the tumor microenvironment, such as CCL2, CCL5, neutrophil elastase (NE), and cathepsin G (CG), are expressed more often in N2 neutrophils, along with a greater expression of arginase. The expression of immuno-activating chemokines and cytokines, such as FAS, ICAM-1, and TNF (tumor necrosis factor) α, is higher in N1 neutrophils.^155^ However, with the spread of single-cell RNA-sequencing, like TAM, TAN is no longer defined as only N1 and N2 types. Using nine recently acquired specimens, Shi et al.^156^ used scRNA-seq to depict the subtype-specific transcriptome landscape of TANs in advanced NSCLC. Seven distinct subtypes of tumor-associated neutrophils (TANs) have been identified. The N3 subtype, known for its inflammatory characteristics due to the expression of genes related to multiple chemotactic cytokines, has been linked to poor overall survival, suggesting its role in promoting tumor growth. In contrast, the N1 and N5 subtypes appear to be mature, well-differentiated neutrophils, characterized by CXCR2 expression and specific pseudo-time patterns; these subtypes are notably prevalent in lung adenocarcinoma cases. Interactions between TAN subtypes and macrophages, primarily through chemokine signaling pathways within TME, have been observed. Xue et al.^157^ discovered that TAN populations prevalent in the myeloid-cell-enriched subtype of liver tumors are linked to a poor prognosis. Through in vitro induction and ex vivo patient analysis, it has been demonstrated that CCL4^+^ TANs can recruit macrophages and PD-L1^+^ TANs are capable of inhibiting T cell cytotoxicity.

More studies have focused on the relationship between TAN recruitment and tumor metastasis. It was found that TANs infiltration is increased in lymph node-metastatic Bladder Cancer (BCa) and is associated with poor prognosis. Neutrophil depletion results in significant reduction in popliteal lymph node metastasis and lymphangiogenesis. Mechanistically, transcription factor ETV4 enhances BCa cells-derived CXCL1/8 to recruit TANs, leading to the increase of VEGFA and MMP9 from TANs, and then facilitating lymphangiogenesis and lymph node metastasis of BCa.^158^ Deng et al.^159^ demonstrate that collagen-induced DDR1 activation in cancer cells is a major stimulus for CXCL5 production, resulting in the recruitment of TAN, the formation of NETs, and subsequent cancer cell invasion and metastasis. Tian et al.^160^ reported that AGR2 released by TANs facilitated the migration of CRC cells. Liver metastases from colorectal cancer were reduced in mice by neutrophil-specific AGR2 ablation. Peripheral blood neutrophils were trained by CRC-derived transforming growth factor beta 1 (TGF-β1) to become AGR2^+^ TANs that release AGR2. High expression of TGF-β1 and CD98hc-xCT, as well as abundant infiltration of AGR2^+^ TANs, were associated with a poor prognosis in CRC patients.

The exploration of tumor-associated neutrophil extracellular traps (NETs) within the tumor microenvironment has garnered significant interest recently. Originally identified as part of the body’s antimicrobial defense, NETs are now understood to significantly influence tumor progression by promoting angiogenesis, aiding metastasis, and inducing tumor-related thrombosis. Compelling evidence indicates that neutrophils can migrate to the site of pre-metastatic niches like lung, liver, and omentum. Once there, they contribute to the remodeling of the local microenvironment via NETs and the secretion of pro-inflammatory mediators that enhance tumor cell extravasation and proliferation.^161^ The emergence of NETs as facilitators in the creation of cancerous pre-metastatic niches and in sustaining secondary inflammatory environments is a current focus of research. Fortunately, advancements have been made in the suppression of NETs through innovative studies. A novel nanocarrier known as mP-NPs-DNase/PTX has been developed, which includes a nanoparticle core of a paclitaxel (PTX) prodrug and a poly-l-lysine shell that is conjugated with an MMP9-cleavable Tat-peptide-coupled deoxyribonuclease I (DNase I). This nanocarrier accumulates at tumor sites and responds to MMP-9 by releasing DNase I, which degrades the NET structure. Subsequently, the exposed cell-penetrating peptide facilitates tumor cell uptake, and the PTX prodrug nanoparticles respond to intracellular glutathione by releasing PTX, thus exerting cytotoxic effects on tumor cells. Both in vitro and in vivo evaluations have confirmed the potential of mP-NPs-DNase/PTX as a NET-regulated therapy for significantly hindering malignant tumor growth and preventing distant metastasis.^162^

### Tumor-associated macrophages

Macrophages located within or around the solid tumor tissues, termed tumor-associated macrophages (TAMs), play a pivotal role in the tumor microenvironment. TAMs influence various processes including tumor growth, angiogenesis, immune regulation, metastasis, and chemoresistance. These cells can exhibit diverse, and sometimes contrasting, phenotypes based on the surrounding microenvironment. Typically, macrophages are categorized into M1 (classical-activated macrophages) and M2 (alternative-activated macrophages) phenotype.^163^ In general, M1 macrophages promote inflammatory responses to combat pathogens and tumor cells, while M2 macrophages suppress immune responses, aiding in tissue repair and tumor progression.

Advancements in single-cell sequencing have revealed a greater complexity in macrophage subpopulations across various cancers. For instance, LYVE1^+^ macrophages, resembling tissue-resident interstitial macrophages, were predominantly found in noncancerous tissues across multiple cancer types. Similarly, NLRP3^+^ macrophages, indicative of pro-inflammatory tissue-resident macrophages, were also prevalent in noncancerous areas. On the other hand, several macrophage subsets within tumors, such as SPP1^+^, C1QC^+^, ISG15^+^, and FN1^+^ TAMs, have been identified, with ISG15^+^ TAMs significantly upregulating interferon-inducible genes. SPP1^+^ TAMs and C1QC^+^ TAMs have displayed diverse functional phenotypes in CRC.^164^ Wu et al.^165^ presented a single-cell and spatial atlas of colorectal liver metastasis and found the highly metabolically activated MRC1^+^CCL18^+^M2-like macrophages in metastatic sites. Efficient neoadjuvant chemotherapy can slow down such metabolic activation, raising the possibility to target metabolism pathways in metastasis.

There are interactions between different types of cells and many reports indicated that TAM promotes tumor metastasis through interaction and dialog with other cells. Wei et al.^166^ indicated that by controlling the JAK2/STAT3/miR-506-3p/FoxQ1 axis, CD163^+^ TAMs induce the EMT program to improve CRC migration, invasion, and CTC-mediated metastasis. This, in turn, results in the production of CCL2, which promotes macrophage recruitment and reveals a new cross-talk between immune cells and tumor cells in the CRC microenvironment. One potential treatment target for preventing tumor spread in gastric cancer is CAP2, a crucial protein that mediates the interaction between tumor-associated macrophages and gastric cancer cells.^167^ CCL18 secreted by TAMs enhances breast cancer metastasis via PITPNM3.^168^ REDD1 deficiency in TAMs leads to the formation of smoothly aligned, pericyte-covered, functional vessels, which prevents vessel leakiness, hypoxia, and metastases. Mechanistically, vascular hyperactivation is prevented and quiescent vascular junctions are formed when highly glycolytic REDD1-deficient TAMs outcompete endothelial cells for glucose consumption. Restoring aberrant angiogenesis and metastasis in REDD1 deficient TAMs requires decreasing glycolysis.^169^ Moreover, macrophage STING signaling enhances NK cell-mediated suppression of CRC liver metastasis through the 4-1BBL/4-1BB pathway^170^ and exosomal ANGPTL1 from Kupffer cells reduces CRC liver metastasis by modulating secretion patterns and counteracting MMP9-induced vascular leakiness.^171^

### Myeloid-derived suppressor cells

MDSCs are heterogeneous and poorly mature cells of innate immunity that their population is increased substantially in cancer patients. A subset of circulating and tumor-infiltrating MPCs is identified as MDSCs due to their innate immune suppressive activity.^172^ They play a crucial role in promoting the survival and expansion of DTCs and micrometastatic sites by helping them evade host immunosurveillance and resist immunotherapy treatments. Moreover, MDSCs aid in reactivating dormant metastases and may influence the organ specificity of metastatic growth by enabling tumor cells to escape adaptive immune responses.^173^ Yang et al.^174^ combined and analyzed recent scRNA-seq datasets of transgenic KPC mouse models with autochthonous PDAC and matched liver metastasis, revealing the unique tumor ecosystem and cell composition of liver metastasis in contrast to primary PDAC. metastatic tumors reveal significantly enriched granulocytic myeloid-derived suppressor cells, mature neutrophils, and granulocyte-myeloid progenitors. Ye et al.^175^ discovered a specific cluster of bone-metastasized prostate cancer cells exhibiting myeloid markers and significant alterations in immune regulation and tumor progression pathways. They observed that these cancer cells likely originated from fusion with bone marrow cells, transforming into myeloid-like cancer cells. Multi-omic analysis indicated significant alterations in pathways related to cell adhesion and proliferation, including focal adhesion, tight junctions, DNA replication, and the cell cycle. Furthermore, Galectin-1 was found to stabilize STING in cancer cells, triggering NF-κB signaling and CXCL2 expression, thereby enhancing MDSC trafficking and supporting the formation of a premetastatic niche that favors metastatic expansion.^176^ Additionally, CXCL1, secreted by TAMs, was shown to boost MDSC proliferation, migration, and suppression of CD8^+^ T-cell activity via CXCR2. Inhibition of CXCR2, either by knockdown or by transplanting CXCR2-deficient MDSCs, significantly reduced stress-induced MDSC accumulation, NET formation, and breast cancer metastasis.^177^

## Effects of metabolic on cancer metastasis

Metabolism is fundamental to cellular and organismal survival, encompassing processes that support growth, reproduction, structural maintenance, and environmental responses. A defining feature of cancer is its metabolic reprogramming, which equips tumor cells to meet the elevated energy demands essential for rapid proliferation, invasion, and metastasis. As distant metastasis evolves, specific metabolic-epigenetic programs are selected that optimize tumorigenic capability. These metabolic alterations interact synergistically with oncogenic signals to modulate cancer cell behavior. For example, mammalian target of rapamycin (mTOR) signaling can alter cancer cell metabolism, and metabolic shifts can subsequently sustain mTOR signaling and promote tumorigenicity. The association between metabolism and tumor metastasis is detailed in Table 2.

**Table 2:** List of literature on metabolic types and tumor metastasis

Metabolism types	Key target	Molecular mechanism	Reference number
Glycolysis	RFX6	RFX6 facilitated aerobic glycolysis-mediated growth and metastasis of HCC through targeting PGAM1.	^178^
	-	Using single-cell RNA sequencing and spatial transcriptomics, a shift from glycolysis to oxidative phosphorylation in a cluster of disseminated cancer cells in breast cancer patients was discovered, highlighting the metabolic evolution critical for early metastasis.	^179^
	circRPN2	CircRPN2 Inhibited aerobic glycolysis and metastasis in HCC.	^180^
	NEAT1	NEAT1 regulated glycolysis through isoform switching, playing a crucial isoform-specific role in breast cancer metastasis independently of paraspeckles.	^33^
	ZEB1	ZEB1, a transcription factor, intensified the Warburg effect in HCC, and ZEB1 does this by activating PFKM, a crucial glycolytic enzyme, thus promoting both tumor growth and metastasis in HCC.	^181^
	CD47	High CD47 expression in CRC correlated with worse prognosis and facilitated cancer growth and metastasis by increasing aerobic glycolysis and activating ERK signaling through the stabilization of ENO1.	^182^
	NK cells	Peripheral blood NK cells from patients with metastatic breast cancer exhibited decreased interferon-γ production, reduced cytotoxicity, and metabolic impairments including lower glycolysis and oxidative phosphorylation, and also displayed significant mitochondrial fragmentation.	^144^
	macrophages	Tumor-derived exosomes (TDEs) caused macrophages to shift their metabolism towards glycolysis, resulting in increased lactate production. This shift enhances PD-L1 expression via an NF-kB-dependent pathway, promoting an immunosuppressive environment that aids tumor metastasis, specifically in lung cancer.	^183^
	macrophages	Exposure to carbon black ultrafine particles shifted lung macrophage metabolism towards glycolysis, increases lactate production, and activates the HIF1α axis, thus leading to mitochondrial damage and reduced respiration and increasing lung cancer incidence and metastasis.	^184^
	TAMs	Increased glucose uptake by M2-like TAMs enhanced the hexosamine biosynthetic pathway, leading to the O-GlcNAcylation of lysosomal Cathepsin B, which promotes cancer metastasis and chemoresistance.	^185^
	FA-EDTA/ICG-Lip NPs	A nanoplatform called FA-EDTA/ICG-Lip NPs targeted breast cancer by inhibiting glycolysis and leveraging acidic environments to enhance immune attacks, thus impeding tumor growth and metastasis.	^186^
	BLG@TPGS NPs	Nanoparticles (BLG@TPGS NPs) targeted and disrupted energy production in TNBC cells by inhibiting mitochondrial functions, including glycolysis, effectively halting tumor growth and spread through a combined chemo-co-starvation therapy approach with reduced toxicity.	^187^
	Nano-activator	A nano-activator that targets and modifies PKM2, triggered by a tumor-related enzyme, formed nanofibers that promote the formation of active PKM2 groups, effectively disrupting the energy supply necessary for tumor growth.	^188^
	DMNPN	The nanoregulator DMNPN inhibited tumor glycolysis and glutaminase, reducing glucose and glutamine uptake by cancer cells and enhancing nutrient availability for T cells, thereby stalling tumor growth and boosting T cell immune efficacy.	^189^
Lipid metabolism	FASN	In male breast cancer, activation of fatty acid metabolism, specifically through the FASN enzyme, was associated with enhanced metastasis of cancer cells and reduced immune cell infiltration.	^190^
	FASN	In cervical cancer, FASN reprogrammed cholesterol metabolism and activates the c-Src/AKT/FAK signaling pathway associated with lipid rafts, resulting in increased cell migration and invasion.	^191^
	FABP5	FABP5 facilitated the reprogramming of fatty acid metabolism, and this metabolic reprogramming leads to an increase in intracellular fatty acid that activate NF-κB signaling, inducing lymph node metastasis in cervical cancer.	^192^
	PTPRO	PTPRO repressed CRC tumorigenesis and progression by activating the AKT/mTOR signaling pathway and reprogramming fatty acid metabolism.	^193^
	HKDC1	HKDC1 reprogrammed lipid metabolism to enhance gastric cancer metastasis and cisplatin resistance via forming a ribonucleoprotein complex.	^194^
	ACSL4	ACSL4 reprograms fatty acid metabolism in hepatocellular carcinoma via c-Myc/SREBP1 pathway and promotes tumor metastasis.	^195^
	macrophages	Exosomes released from PD-L1(+) tumor associated macrophages promote peritoneal metastasis of epithelial ovarian cancer by up-regulating T cell lipid metabolism thus leading to reactive oxygen species accumulation.	^196^
	macrophages	Macrophages with upregulated CD36 expression were enriched their lipid droplets by engulfing tumor-derived long-chain fatty acids via extracellular vesicles. This lipid uptake, facilitated through CD36, not only fuels these macrophages but also triggers liver metastasis.	^197^
	macrophages	Lipid-associated macrophages (LAMs) are enriched in metastasis-bearing lungs and show increased expression of genes related to lipid metabolism, extracellular matrix remodeling, and immunosuppression, indicating a possible role for these cells in promoting cancer progression.	^198^
	mesenchymal cells	Neutrophils interact with lung mesenchymal cells (MCs), accumulate neutral lipids by inhibiting the activity of adipose triglyceride lipase, and transport lipids to metastatic tumor cells through the macrophage-lysosomal pathway, enhancing the survival and proliferation capabilities of tumor cells, thereby promoting metastatic colonization of the lungs.	^199^
	CAFs	CRC cells, through their exosomes rich in HSPC111, impacted liver metastasis by reprogramming the lipid metabolism of liver CAFs.	^107^
	CAFs	CAFs triggered lipid metabolism reprogramming by upregulating FASN, increasing the production of lipid metabolites, and promoting the migration of CRC cells through these products.	^200^
	CAFs	Phosphatidylcholine containing unsaturated fatty acid chains secreted by CAFs increased the membrane fluidity of CRC cells and promotes sugar uptake and metabolism, thereby enhancing peritoneal metastasis.	^201^
Arginine metabolism	VIPR1	Activating VIPR1 with VIP led to an increase in ASS1, an enzyme vital for arginine biosynthesis, and this pathway played a critical role in the suppression of HCC growth and metastasis.	^202^
	FOXO3a	FOXO3a levels are inversely correlated with ASS1 and ASL across different tumor stages, impacting both esophageal squamous cell growth and metastasis.	^203^
	RIOK3	RIOK3 boosted the expression of the arginine transporter SLC7A2, promoting cell invasion and metastasis in PDAC.	^204^
	HN-HFPA	A multifunctional nanoplatform (HN-HFPA) modulated arginine metabolism in TAMs within TNBC, enhancing immune responses and inhibiting tumor growth through targeted release of L-arginine and L-norvaline.	^205^
Glutamine metabolism	GOT2	GOT2 silencing promoted reprogramming of glutamine metabolism and sensitized HCC to glutaminase inhibitors.	^206^
	ANGPTL4	ANGPTL4 altered glutamine usage and fatty acid oxidation and influences energy metabolism and tumor spread in NSCLC.	^207^
	glutamine	Inhibition of glutamine metabolism decreased the generation and recruitment of MDSCs and promoted the transformation of TAMs towards an antitumor phenotype.	^208^
Serine metabolism	ZEB1	ZEB1 reprogrammed the de novo serine synthesis pathway by transcriptionally activating PHGDH, thus promoting carcinogenesis and metastasis in HCC.	^209^
	PSPH	PSPH promoted melanoma growth and metastasis by metabolic deregulation-mediated transcriptional activation of NR4A1.	^210^
	PHGDH	Modification of PHGDH by a protein complex increased serine and S-adenosylmethionine levels, enhancing gene expression for cell adhesion and promoting CRC metastasis.	^211^
Asparagine metabolism	DON	Glutamine mimicry suppressed tumor progression through asparagine metabolism in PDAC.	^212^
	NUCKS1	NUCKS1increased the levels of asparagine by promoting the expression of ASNS, and this rise in asparagine fuels the growth and spread of osteosarcoma cells.	^213^
	ASNS	ASNS was found to be highly expressed in oral squamous cell carcinoma, correlating positively with lymph node metastasis and perineural invasion.	^214^

### Glucose metabolism

**Fig. 5:**
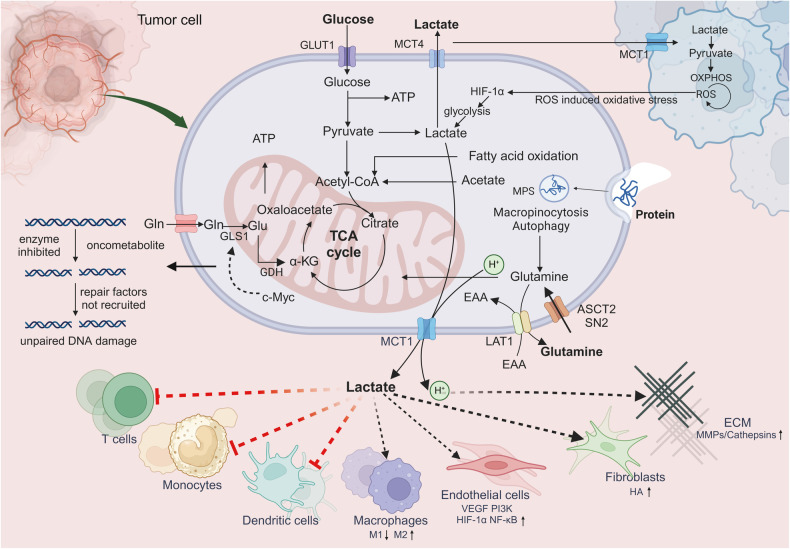
Tumor metabolism can be divided into three levels: 1. Involving the intake of nutrients such as glucose, amino acids, and nitrogen. Immune cells mainly consume glucose, while tumor cells mainly consume amino acids and fatty acids. Glutamine serves as a nitrogen donor for purines, pyrimidines, and non essential amino acids, as well as a carbon donor. Cancer cells can obtain amino acids from extracellular proteins through microcytosis. 2. Changes in metabolic processes: Tumor cells utilize TCA cycle intermediates for NADPH biosynthesis and production. At the same time, the increase in nitrogen demand leads to an upregulation of the transcription factor c-Myc in proliferating cells, leading to increased uptake of glutamine by cells. 3. The role of metabolites: Tumor metabolites can drive abnormal gene expression and interact with the surrounding microenvironment. The high utilization of extracellular glucose and glutamine by cancer cells leads to the accumulation of extracellular lactate, which has been shown to affect several cell types in the tumor microenvironment. The increase in lactate levels also promotes the emergence of favorable microenvironments for tumor cells. Created with BioRender.com

Glycolysis, a fundamental metabolic pathway that processes glucose to pyruvate for ATP production, has been increasingly recognized for its crucial role in cancer metastasis. The Warburg effect, a phenomenon where cancer cells preferentially rely on glycolysis for energy production even in the presence of oxygen, underscores the metabolic reprogramming in the tumor microenvironment. The aforementioned alteration not only caters to the heightened energy demand of proliferating cancer cells, but also contributes to the creation of an acidic microenvironment that is conducive to metastasis (Fig. 5).

Recent studies have elucidated the mechanisms through which glycolysis influences tumor metastasis. For instance, Qiu et al.^178^ revealed that elevated regulatory factor X6 (RFX6) expression serves as an independent prognostic factor associated with poor outcomes in HCC. A key mechanism underlying this process is RFX6’s transcriptional upregulation of phosphoglycerate mutase 1 (PGAM1), a key enzyme in glycolysis. By augmenting glycolysis, RFX6 accelerates the progression of HCC, particularly its metastatic potential. Utilizing scRNA-seq and spatial transcriptomics, Liu et al.^179^ profiled 65,968 cells from breast cancer patients with metastatic axillary lymph nodes. They identified a disseminated cancer cell cluster exhibiting high levels of oxidative phosphorylation (OXPHOS), suggesting a transition from glycolysis to OXPHOS during dissemination. This shift in metabolic profile was observed at the leading edge of the tumor, indicating its significance in early metastasis. Li et al.^180^ found that circRPN2 is downregulated in highly metastatic HCC cell lines and tissues, indicating its potential involvement in tumor metastasis. Mechanistically, the study revealed that circRPN2 binds to enolase 1 (ENO1) and accelerates its degradation, promoting glycolytic reprogramming through the AKT/mTOR pathway. This process inhibits HCC metastasis. Park et al.^33^ demonstrated that NEAT1, a long non-coding RNA, facilitates the assembly of glycolytic complexes, including PGK1, PGAM1, and ENO1, which are critical enzymes involved in the glycolytic process. This assembly enables efficient substrate channeling and promotes a glycolytic state in cancer cells. This isoform-specific regulation of NEAT1 appears to be critical for the metastatic potential of breast cancer cells. Zhou et al.^181^ discovered that ZEB1, a transcription factor known for its role in cancer metastasis, enhances the Warburg effect in HCC. This effect, characterized by increased glycolysis even in the presence of oxygen, is a crucial metabolic reprogramming event in tumor initiation and progression. Hu et al.^182^ reported that CD47 is significantly upregulated in CRC and its high expression correlates with poor prognosis. The study revealed that CD47 promotes CRC cell growth and metastasis through enhancing aerobic glycolysis and activating ERK signaling. The mechanism behind this involves CD47’s interaction with ENO1, an enzyme in the glycolysis pathway. CD47 stabilizes ENO1 by protecting it from ubiquitin-mediated degradation.

Immune cells, the sentinels of the body’s defense system, are known to infiltrate tumor environments, where they can either suppress or promote metastasis. A critical aspect of their function in the TME is their metabolic activity, particularly glycolysis, which has far-reaching implications for cancer dissemination. The metabolic reprogramming of immune cells is necessary to fulfill the requirements of rapid proliferation and effector function, resembling the Warburg effect observed in cancer cells. This metabolic shift towards increased glycolysis is essential for the efficient functioning of immune cells within the TME. Slattery et al.^144^ investigated the metabolic dysfunction of NK cells in the context of human metastatic breast cancer. The researchers discovered that patients with metastatic breast cancer exhibited impaired production of IFN-γ, reduced cytotoxicity, and evident metabolic deficiencies in their peripheral blood NK cells, including decreased glycolysis and oxidative phosphorylation. Morrissey et al.^183^ discovered that tumor-derived exosomes reprogram macrophages towards a glycolytically dominant metabolism, which increases lactate production and elevates PD-L1 expression through an NF-kB-dependent pathway. This process makes the macrophages more immunosuppressive, potentially aiding the tumor cells in evading the immune response in the pre-metastatic niche, thereby promoting lung cancer metastasis. Chang et al.^184^ found that exposure to carbon black ultrafine particles (nCB) reprograms lung macrophages, leading to a metabolic shift towards glycolysis and increased lactate production. This shift results in a sustained HIF1α axis that supports a glycolytically dominant metabolism in macrophages. The study demonstrated that nCB-exposed lung macrophages exhibit selective mitochondrial damage and decreased oxidative respiration. Shi et al.^185^ and colleagues reported that M2-like TAMs use increased glucose uptake to fuel the hexosamine biosynthetic pathway. This leads to enhanced protein O-GlcNAcylation, particularly of lysosomal Cathepsin B, which is mediated by lysosome-localized O-GlcNAc transferase (OGT). The O-GlcNAcylation of Cathepsin B at serine is crucial, as it increases the mature form of this protease in macrophages and its secretion into the tumor microenvironment.

The utilization of nanotechnology in strategic interventions against cancer metastasis presents a promising frontier in the field of oncology, capitalizing on the distinctive properties of nanomaterials to precisely target the intricate mechanisms underlying tumor dissemination. The application of nanomaterials in inhibiting cancer metastasis represents a significant advance, particularly through the modulation of tumor cell metabolism, such as glycolysis. Zhou et al.^186^ developed a nanoplatform, FA-EDTA/ICG-Lip NPs, that innovatively targets breast cancer metastasis by impeding glycolysis, a key metabolic pathway crucial for cancer progression. The team encapsulated ethylenediaminetetraacetic acid (EDTA) and indocyanine green (ICG) within liposomes, which were further modified with folic acid (FA) to ensure targeted delivery to cancer cells. Moreover, the nanoplatform exploits the acidic environment of lysosomes to release EDTA and ICG, achieving a two-pronged strategy: reducing extracellular matrix (ECM) production by down-regulating transforming TGF-β and activating an immune response through the induction of tumor cell immunogenic cell death (ICD). Zhang et al.^187^ found that mitochondria-anchoring self-assembled nanoparticles (BLG@TPGS NPs) significantly inhibit TNBC metastasis by targeting mitochondria to induce energy depletion. This “nano bomb” synergizes chemotherapy with co-starvation therapy, efficiently eradicating cancer cells. The nanoparticles, incorporating the energy inhibitors Berberine and Lonidamine, along with the chemotherapeutic agent Gambogic acid, demonstrate a targeted ability to accumulate in mitochondria—the cell’s energy factory. Hou et al.^188^ developed a glycopeptide-based PKM2 nano-activator capable of significantly inhibiting tumor proliferation, chemoresistance, and metastasis by inducing the tetramerization of pyruvate kinase muscle isoform 2 (PKM2), a critical enzyme in aerobic glycolysis. This nano-activator, upon activation by O-GlcNAcase (OGA) — an enzyme upregulated in many human tumors—transforms into nanofibers that specifically target and bind to PKM2, promoting its tetramerization. The nano-activator’s targeted action not only disrupts the tumor cells’ metabolic processes but also enhances the effectiveness of chemotherapy drugs against prostate and breast cancers by increasing drug sensitivity at the tumor sites. Zhang et al.^189^ and colleagues developed a novel dual-mechanism based nutrient partitioning nanoregulator (DMNPN) that simultaneously tackles the TME and boosts nutrient availability for T cells, thus enhancing antitumor immunity. By inhibiting glycolysis and downregulating glutaminase, DMNPN restricts glucose and glutamine uptake by tumor cells, shifting nutrient availability towards T cells. This approach significantly hampers the growth of tumors resistant to anti-programmed death receptor 1 (anti-PD1) therapy and curtails tumor metastasis and recurrence.

### Lipid metabolism

**Fig. 6:**
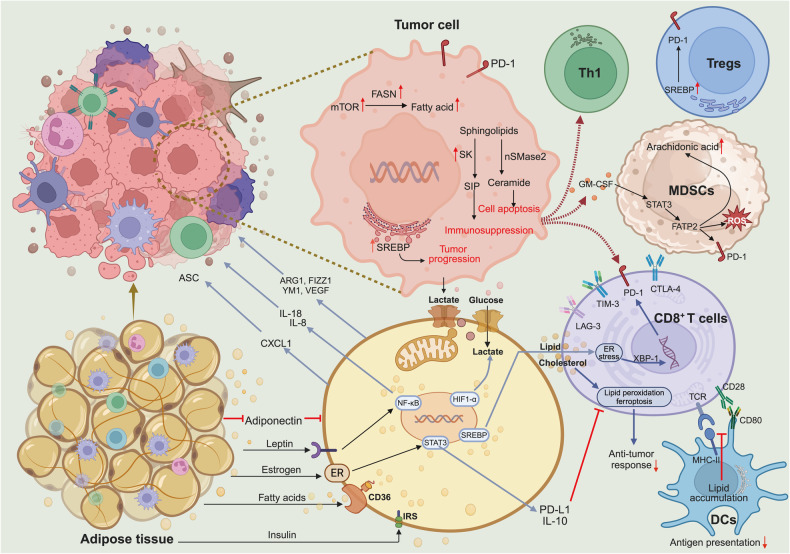
Lipid metabolism and its role in cancer metastasis. This figure illustrates the critical role of lipid metabolism in the progression and dissemination of cancer cells. Highlighted are the key functions of lipids beyond energy storage, including their involvement in membrane formation, energy production, and modulation of signaling pathways that influence cell migration, invasion, and survival. The figure also encapsulates significant research findings, such as the correlation between fatty acid metabolism and immunosuppression in male breast cancer, the prognostic value of fatty acid synthase (FASN) expression in cervical cancer, and the impact of metabolic reprogramming on lymph node metastasis. Additionally, it underscores the influence of lipid metabolism on immune cell function and the tumor-promoting activities of metastasis-associated macrophages and cancer-associated fibroblasts. Created with BioRender.com

In the complex landscape of cancer metastasis, lipid metabolism has emerged as a pivotal player influencing the malignant progression and dissemination of tumor cells. Alterations in lipid metabolism are not merely byproducts of cancer cell transformation but actively contribute to the acquisition of metastatic traits (Fig. 6). This phenomenon underscores the multifaceted role of lipids beyond their traditional functions as energy storage molecules, membrane constituents, and signaling mediators. Lipids, particularly fatty acids, play a crucial role in the formation of new membranes, energy production, and the modulation of signaling pathways that govern cell migration, invasion, and survival in distant tissues. Sun et al.^190^ conducted a single-cell transcriptome analysis of male breast cancer, uncovering significant insights into the role of fatty acid metabolism in cancer metastasis and immunosuppression. Their research revealed that MBC exhibits a notably lower infiltration of T cells compared to female breast cancer (FBC), with metastasis-related programs being more active in MBC cancer cells. Central to their findings is the observation that the activation of fatty acid metabolism, particularly involving the enzyme FASN, correlates with enhanced cancer cell metastasis and reduced immune cell infiltration in MBC. Du et al.^191^ demonstrated that elevated expression of fatty acid synthase (FASN) not only predicts a poor prognosis for cervical cancer (CC) patients but also facilitates CC cell migration, invasion, and lymphangiogenesis. Mechanistically, FASN reprograms cholesterol metabolism, which subsequently activates the lipid raft-related c-Src/AKT/FAK signaling pathway, leading to increased cell migration and invasion. Zhang et al.^192^ demonstrated that fatty acid-binding protein 5 (FABP5), significantly upregulated in cervical cancer with lymph node metastasis (LNM), serves as an independent predictor for LNM, correlating with poorer prognosis. Through a series of both in vitro and in vivo experiments, the researchers revealed that FABP5 facilitates the reprogramming of fatty acid (FA) metabolism, which includes promoting lipolysis and FA synthesis. This metabolic reprogramming leads to an increase in intracellular FAs that activate NF-κB signaling, inducing LNM. Dai et al.^193^ revealed that protein tyrosine phosphatase receptor type O (PTPRO) expression was significantly downregulated in CRC liver metastasis compared to primary tumors, correlating with a poorer prognosis for patients. PTPRO silencing led to the activation of the AKT/mTOR signaling pathway, enhancing de novo lipogenesis by upregulating sterol regulatory element-binding protein 1 (SREBP1) and its target lipogenic enzyme, acetyl-CoA carboxylase alpha (ACC1). The RNA-binding protein hexokinase domain component 1 (HKDC1) is overexpressed in gastric cancer and plays a significant role in the disease’s progression and chemoresistance. High HKDC1 levels were associated with poor prognosis in gastric cancer patients, correlating with enhanced invasion, migration, and resistance to cisplatin in vitro and in vivo. Through comprehensive transcriptomic sequencing and metabolomic analysis, the study unveiled that HKDC1 mediates abnormal lipid metabolism in gastric cancer cells, identifying a novel regulatory axis involving HKDC1, G3BP1, and the protein kinase PRKDC.^194^ Chen et al.^195^ unveiled how ACSL4, an enzyme linked to fatty acid metabolism, plays a critical role in promoting HCC progression. They discovered that ACSL4 enhances the synthesis of lipids within HCC cells by activating the c-Myc/SREBP1 signaling pathway, which leads to the upregulation of key enzymes involved in lipid production.

Immunometabolism, an emerging field at the intersection of immunology and metabolism, has unveiled the profound impact of metabolic pathways on the immune system’s ability to combat cancer. Among these pathways, lipid metabolism within immune cells stands out for its crucial role in modulating tumor progression and metastasis. The ability of immune cells to prevent or promote metastasis is closely linked to their metabolic programming, particularly lipid metabolism, which provides the necessary energy and building blocks for their function. PD-L1^+^ TAMs release exosomes that significantly increase the expression of the transcription factor PPARα and the enzyme CPT1A in CD8^+^ T cells, thereby enhancing fatty acid oxidation and inducing reactive oxygen species accumulation. This metabolic reprogramming leads to increased apoptosis and exhaustion in CD8^+^ T cells, marked by elevated levels of exhaustion markers such as LAG3, TIM-3, and PD1. The impaired function of CD8^+^ T cells contributes to a peritoneal immune imbalance, ultimately facilitating the peritoneal metastasis of epithelial ovarian cancer.^196^ Yang et al.^197^ reported that metastasis-associated macrophages (MAMs), which exhibit upregulated CD36 expression, are enriched with lipid droplets due to their enhanced capacity to engulf tumor cell-derived long-chain fatty acids carried by extracellular vesicles. This lipid acquisition, facilitated through CD36, not only fuels the macrophages but also triggers their tumor-promoting activities. To explore the macrophage heterogeneity in metastatic mammary tumors, Huggins et al.^198^ identified seven distinct macrophage clusters through scRNA-seq of macrophages from the lungs of mice. Notably, one cluster exhibited a lipid-associated profile, marked by increased expression of genes related to lipid metabolism, such as Lgals3 and Trem2. Neutrophils, when interacting with lung mesenchymal cells in a pre-metastatic lung environment, accumulate neutral lipids through a process that involves the suppression of adipose triglyceride lipase (ATGL) activity. This suppression occurs in both prostaglandin E2-dependent and -independent manners. This transfer of lipids from neutrophils to tumor cells enhances the survival and proliferative capacities of the cancer cells, thereby promoting metastatic colonization in the lung.^199^

As integral components of the stromal compartment, CAFs not only provide structural support but also actively participate in biochemical signaling cascades that potentiate tumor growth, invasion, and dissemination. Among the diverse metabolic pathways operational within CAFs, lipid metabolism has emerged as a pivotal regulator of their pro-tumorigenic activities. Zhang et al.^107^ found that exosomes from CRC cells contain high levels of HSPC111, a gene previously associated with cancer progression. These exosomes are taken up by CAFs in the liver, leading to significant changes in the lipid metabolism of these fibroblasts. Specifically, HSPC111 in the exosomes phosphorylates ATP-citrate lyase (ACLY), increasing the level of acetyl-CoA in CAFs. Gong et al.^200^ found that CAFs undergo lipidomic reprogramming, accumulating fatty acids and phospholipids, which significantly enhances the migration of CRC cells compared to normal fibroblasts. Inhibition of FASN in CAFs or blocking the uptake of fatty acids by CRC cells, either through siRNA-mediated knockdown or by using sulfo-N-succinimidyloleate sodium and CD36 monoclonal antibody, successfully reduced CRC cell migration in vitro and in vivo. Lipids secreted by CAFs, particularly phosphatidylcholine with unsaturated acyl chains, were increased in CRC cells, leading to enhanced membrane fluidity. This alteration in membrane properties facilitated increased glucose uptake and metabolism in CRC cells, promoting cell invasiveness and metastatic potential.^201^

### Amino acid metabolism

**Fig. 7:**
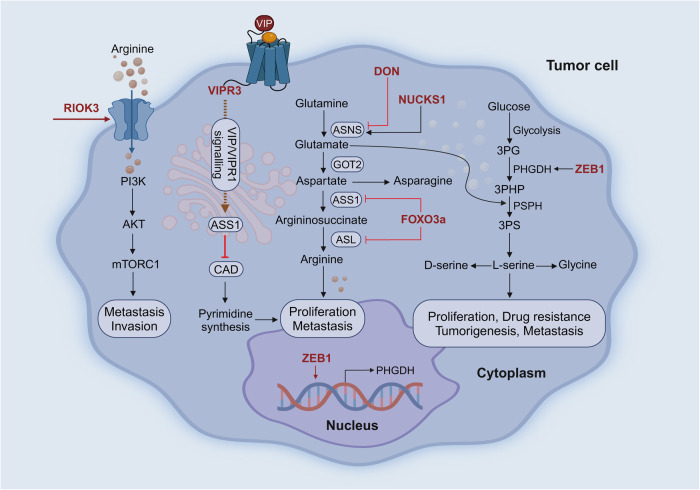
Amino acid metabolism in cancer metastasis. Metabolic rewiring: Illustrates the critical role of amino acid metabolism reprogramming in supporting tumor growth, immune evasion, and metastatic spread. Arginine metabolism: Highlights the influence of VIPR1 activation and FOXO3a-regulated metabolic plasticity on HCC and ESCC progression, with a focus on ASS1 and ASL enzymes. Glutamine and Serine metabolism: Shows the impact of GOT2 downregulation and ZEB1-mediated SSP reprogramming on HCC, and the role of ANGPTL4 in NSCLC metastasis. Asparagine metabolism: Depicts the effects of targeting glutamine metabolism in PDAC and the significance of ASNS in various cancers, emphasizing its role in tumor cell proliferation and metastasis. Created with BioRender.com

In the intricate network of cancer progression, amino acid metabolism stands out as a pivotal biochemical pathway, orchestrating a multitude of cellular processes critical for tumor growth and metastasis. The reprogramming of amino acid metabolism in cancer cells not only supports rapid proliferation by satisfying increased demands for energy and biosynthetic precursors but also plays a vital role in evading immune surveillance and promoting invasive behavior, thereby facilitating metastasis (Fig. 7). This adaptive metabolic rewiring allows cancer cells to thrive in the nutrient-deprived and harsh microenvironments of primary and distant sites, highlighting its importance in the metastatic cascade.

### Arginine metabolism

Vasoactive Intestinal Peptide Receptor 1 (VIPR1) expression is significantly reduced in human HCC tissues and is associated with advanced clinical stages, tumor growth, recurrence, and poor patient outcomes. Activation of VIPR1 by its ligand, VIP, was found to inhibit HCC growth and metastasis in both in vitro and in vivo models. The mechanistic exploration revealed that VIP treatment through VIPR1 activation leads to the upregulation of argininosuccinate synthase (ASS1), an enzyme critical for arginine biosynthesis, and to the inhibition of the de novo pyrimidine synthetic pathway by downregulating the activation of CAD.^202^ Sun et al.^203^ discovered a critical role of FOXO3a-regulated arginine metabolic plasticity in esophageal squamous cell carcinoma (ESCC) progression, which adaptively promotes both tumor proliferation and metastasis. Levels of argininosuccinate synthetase 1 (ASS1) and argininosuccinate lyase (ASL), key enzymes in arginine metabolism, were increased in primary ESCC tumors but decreased in lymph-metastatic tumor tissues. Intriguingly, FOXO3a levels inversely correlated with ASS1 and ASL across tumor stages, indicating differential regulation by FOXO3a. Silencing ASS1/ASL hindered primary tumor growth while encouraging metastasis. The Rio Kinase 3 (RIOK3) significantly influences arginine metabolism in PDAC, thus affecting tumor proliferation, invasion, and metastasis. Through extensive metabolomic and transcriptomic analyses, RIOK3 promotes the expression of the arginine transporter solute carrier family 7 member 2 (SLC7A2), enhancing arginine uptake and activating the mechanistic target of rapamycin complex 1 (mTORC1).^204^ Chen et al.^205^ developed a multifunctional nanoplatform (HN-HFPA) that remodels the immunosuppressive tumor microenvironment (ITM) in TNBC through arginine metabolism modulation in TAMs. This innovative approach involves the use of a hollow nanoplatform loaded with L-arginine (L-Arg) and coated with L-norvaline (L-Nor) modified hyaluronic acid (HA) to target CD44-overexpressing tumor cells.

### Glutamine metabolism

Downregulation of Glutamic-oxaloacetic transaminase 2 (GOT2) in HCC is associated with advanced disease stages and poor patient outcomes, signifying its potential as a prognostic marker. Silencing of GOT2 facilitates tumor migration and invasion by augmenting glutaminolysis, nucleotide biosynthesis, and glutathione synthesis, thereby reprogramming glutamine metabolism.^206^ Song et al.^207^ discovered the role of Angiopoietin-like protein 4 (ANGPTL4) in regulating glutamine metabolism and fatty acid oxidation, significantly influencing energy metabolism and tumor metastasis in NSCLC. Knocking down ANGPTL4 led to inhibited tumor metastasis and energy metabolism in mouse models, with minimal effects on glycolysis. Inhibition of glutamine metabolism notably decreases the generation and recruitment of MDSCs and promotes the transformation of TAMs towards an antitumor phenotype. The mechanism involves blocking glutamine metabolism in the tumor and myeloid cells, which leads to reduced immunosuppressive cells in the TME and increased inflammatory macrophages, enhancing antitumor immunity.^208^

### Serine metabolism

ZEB1 reprograms the de novo serine synthesis pathway (SSP) by transcriptionally activating phosphoglycerate dehydrogenase (PHGDH), thus promoting carcinogenesis and metastasis in HCC. ZEB1 directly binds to a non-classic binding site within the promoter region of PHGDH, leading to its upregulation. This activation of PHGDH increases SSP flux, enabling HCC cells to become more invasive, proliferative, and resistant to oxidative stress and the chemotherapy agent sorafenib.^209^ Rawat et al.^210^ demonstrated that phosphoserine phosphatase (PSPH), a crucial enzyme in the serine metabolism pathway, is significantly upregulated in patient-derived melanoma samples. Knocking down PSPH expression using short hairpin RNAs (shRNAs) markedly inhibited melanoma tumor growth and metastasis in both cell culture and mouse models. Zhang et al.^211^ revealed a significant link between serine metabolism and tumor metastasis in CRC, focusing on PHGDH. They found that PHGDH, when monoubiquitinated by the cullin 4A–based E3 ligase complex, increases its activity, leading to elevated levels of serine, glycine, and S-adenosylmethionine (SAM). This metabolic shift facilitates the upregulation of cell adhesion genes, thereby augmenting tumor cell migration and promoting CRC metastasis.

### Asparagine metabolism

In PDAC, targeting glutamine metabolism with 6-diazo-5-oxo-L-norleucine (DON) significantly hampers tumor growth and metastasis. This effect is mediated through the suppression of asparagine synthesis by inhibiting asparagine synthetase (ASNS). DON’s inhibition of glutamine metabolism reduces asparagine production, a critical amino acid for tumor cell growth and proliferation.^212^ Nuclear ubiquitous casein and cyclin-dependent kinase substrate 1 (NUCKS1) plays a pivotal role in promoting osteosarcoma progression and metastasis through the upregulation of asparagine synthesis. NUCKS1, which is significantly increased in osteosarcoma, enhances asparagine synthesis by transcriptionally upregulating ASNS expression. This elevation in ASNS leads to increased asparagine levels in osteosarcoma cells, fueling cell growth and metastasis.^213^ Fu et al.^214^ conducted targeted metabolomics to quantify amino acids in oral squamous cell carcinoma (OSCC) tissues, identifying significantly elevated levels of asparagine in perineural invasion (PNI)-positive cases. The key enzyme for ASNS, was found to be highly expressed in tumor cells, correlating positively with lymph node metastasis and PNI. In vivo experiments demonstrated that high ASNS expression promoted nerve invasion and tumor metastasis.

## Peripheral immunity and cancer metastasis

Peripheral immunity, encompassing the body’s systemic defense mechanisms outside of the primary tumor microenvironment, plays a pivotal role in either thwarting or facilitating tumor metastasis. The intricate interplay between peripheral immunity and tumor metastasis represents a pivotal domain of cancer research, elucidating the ambivalent role of immune mechanisms in the progression and dissemination of malignancies. This intricate interplay encompasses a diverse array of immune cells, signaling pathways, and molecular mediators that delicately navigate the fine balance between immunosurveillance and evasion by tumors. On one hand, the immune system exhibits the capacity to identify and eliminate circulating tumor cells, thereby impeding their dissemination to distant organs. Conversely, specific constituents of the immune system can be hijacked by neoplastic cells to facilitate their survival, proliferation, and colonization in novel sites.^215^ This paradoxical role of peripheral immunity in tumor metastasis underscores the sophisticated mechanisms by which tumors adapt and evolve to circumvent immune destruction, highlighting the necessity of understanding these processes for the development of effective cancer therapies. The following sections delve into the specific roles of lymph nodes, CTCs, and exosomes in this dynamic interplay, shedding light on their contributions to the metastatic cascade.

### Lymph nodes

Lymph nodes (LNs), as pivotal constituents of the peripheral immune system, play a paradoxical role in the process of cancer metastasis. Initially, they act as critical barriers, employing their immune arsenal to intercept and potentially eradicate metastasizing cancer cells. However, the relationship between lymph nodes and metastatic cancer cells is not merely antagonistic. Animal studies have strongly supported the role of lymph node invasion in tumor metastasis. A recent study showed that, in a syngeneic melanoma mouse model, lymph node metastases (LNMs) were capable of resisting T-cell-mediated cytotoxicity, inducing antigen-specific regulatory T cells, and generating tumor-specific immune tolerance, thereby facilitating distant tumor colonization.^216^ The orthotopic transplantation of VEGF-C-overexpressing breast cancer cells onto nude mice increased intratumoral lymphangiogenesis and significantly promoted tumor metastasis to the regional LNs and lungs.^217^ Similarly, VEGF-A and -D has been observed to trigger lymphangiogenesis in tumors and lymphatic metastasis to promote tumor metastasis.^218^ In clinical setting, lymph node metastasis increased the incidence of distant metastasis in thyroid cancer.^219^ In addition, Naxerova et al.^220^ found that 35% of CRC’s distant metastases originated from LNMs, a finding echoed by David and colleagues, who reported a similar origin for 25% of breast cancer metastases.^221^ However, several clinical intervention trials showed that prophylactic LN dissection might not improve overall survival in breast cancer,^222^ melanoma,^223^ and lung cancer.^224^ A long-term follow-up of a randomized trial demonstrated that localized lymph node metastasis, distant metastasis and patient’s survival were not strongly correlated.^225^ In addition, metastatic CRC patients with positive LNs exhibited a lower incidence of lung metastasis.^226^ In contrast, it should be noted that in some types of cancers, patients with positive LNs typically have worse clinical outcomes than those with negative LNs. For instance, metastasis of squamous cell carcinoma of the penis to inguinal LNs is associated with poor clinical outcomes.^227^ Similar findings were reported in pancreatic cancer^228^ and nasopharyngeal carcinoma.^229^ Collectively, these studies exemplify the multifaceted roles played by lymph nodes in cancer metastasis. Therefore, comprehending the intricate dynamics within lymph nodes and their impact on cancer metastasis is imperative for devising targeted therapies that can effectively combat this devastating facet of cancer progression.

### Circulating tumor cells

As solid tumors advance, alterations in the peritumoral microenvironment emerge, leading certain tumor cells to manifest aberrant behavior. These cells, upon detachment from the primary tumor, navigate to diverse body tissues and organs via the bloodstream or lymphatic system. These disseminated tumor cells within the bloodstream are termed circulating tumor cells.^230^ During breast cancer evolution, *HER2*-negative (*HER2* − ) breast cancers manifest *HER2*-CTC expression, intensifying breast cancer metastasis.^231^ Notably, heparinase activity suppression or inhibition using the heparinase inhibitor JG6 effectively halts CTC cell cluster formation, impeding breast cancer metastasis progression.^232^ Due to its potential utility as a tumor marker to gauge metastatic extent in conditions like NSCLC, CRC, and brain cancer, CTCs have become a focal point in metastasis research.^233–235^

### Exosomes

Within the TME, cancer cells and their various cellular constituents release exosomes that operate in either autocrine or paracrine modes, facilitating tumor-induced immunosuppression, angiogenesis, and the formation of pre-metastatic niches. During primary tumor establishment, there is an imperative for tumor cells to interact actively with adjacent cells and the surrounding microenvironment. This exosome-mediated cell-cell communication primarily transpires via three mechanisms: firstly, exosomal membrane proteins can engage with target cell membrane proteins, thereby initiating intracellular signaling pathways. Secondly, in the extracellular matrix, proteases can cleave exosomal membrane proteins, allowing resultant fragments to act as ligands that interact with cell membrane receptors, subsequently triggering intracellular signaling pathways. Thirdly, the exosome membrane may directly fuse with the target cell membrane, resulting in a non-selective release of its protein, mRNA, and miRNA contents. Notably, pre-metastatic niches, established by primary tumors, play a pivotal role in cancer metastasis. For instance, lung epithelial cells detect tumor exosomal RNA via Toll-like receptor 3 (TLR3), which instigates neutrophil recruitment and subsequent formation of lung metastasis niches.^236^ Tumor-derived exosomes, vesicles enriched with DNA, mRNA, miRNA, and proteins, have been posited to reconstruct the pre-metastatic environment even before the arrival of cancer cells,^237^ facilitating pivotal roles in cell-cell communication by transferring RNAs and proteins to recipient cells.^238^ Specific fibrotic cytokines, notably TGF-β, activate hepatic stellate cells (hStCs), leading to fibronectin production which then forms a fibrotic microenvironment conducive to the recruitment of bone marrow-derived dendritic cells (BMDCs), encompassing macrophages and neutrophils.^239^ In the realm of lung epithelial cells, tumor-derived exosomal RNAs, particularly rich in small nuclear RNA, activate TLR3, triggering chemokine secretion and driving neutrophil recruitment.^236^ Extracellular vesicles (EVs) are believed to mediate various invasion processes, potentially assisting in the initial metastatic stages.^240^

During the exosome transfer, unshielded RN7SL1 induces an inflammatory response. In breast cancer cells, this unshielded RN7SL1 activates the pattern recognition receptor (PRR) RIG-I, thereby augmenting tumor progression, metastasis, and resistance to therapy.^241^ Furthermore, tumor-derived exosomes can modulate macrophages towards an immunosuppressive phenotype, furthering tumor metastasis.^183^ In melanoma, exosomes, which constitute the majority of EVs from metastatic melanomas, exhibit PD-L1 on their surfaces. PD-L1 presence on these vesicles is augmented by IFN-γ stimulation, impairing CD8^+^ T cell function and facilitating tumor progression. In patients with metastatic melanoma, circulating exosomal PD-L1 levels correlate positively with IFN-γ concentrations.^242^ Given these mechanisms, exosomes have found considerable clinical applications. As next-generation biomarkers in cancer diagnosis and treatment, circulating exosomes with tumor-specific molecular markers, such as oncoproteins, mRNAs, LncRNAs, and DNA fragments, demonstrate significant clinical relevance.^243^ Distinct expression of integrin β4 and integrin αv in patient-derived exosomes might signal metastasis to the liver or lung, respectively. Hence, exosome-specific integrins appear to guide the formation of pre-metastatic niches in particular organs, influencing organotropic metastatic growth.^244^ This knowledge enhances the diagnostic and therapeutic approaches to tumors, particularly in the context of metastatic cancer formation, which is a complex and multistage process. The treatment strategy for metastatic cancer should be comprehensive, targeting both the unique characteristics and vulnerabilities of metastatic cancer cells through a multi-targeted and multifaceted approach. As our understanding of this disease continues to expand, we anticipate improved clinical outcomes for patients with metastatic cancer.

## Mechanical forces and cancer metastasis

The role of mechanical forces in tumor metastasis represents a critical, yet often underappreciated, facet of cancer progression. Beyond the genetic and molecular aberrations driving oncogenesis, the physical interactions between tumor cells and their microenvironment significantly contribute to metastatic dissemination.^245^ These forces, arising from the tumor’s mechanical milieu, include breaching ECM barriers, hemodynamic shear stress, and microvascular resistance. Each of these forces influences not only the physical translocation of cancer cells but also their phenotypic traits, enhancing their ability to survive, invade, and ultimately colonize distant tissues. The process begins with the detachment of cancer cells from the primary tumor, where they must navigate through a dense and cross-linked ECM. This navigation is facilitated by forces generated by the cancer cells, which remodel the ECM to create viable pathways for invasion. Upon entering the bloodstream, these cells are subjected to the shear stress of blood flow, a factor that selects for cells capable of withstanding these conditions. Finally, as these cells traverse the vasculature to reach distant sites, they encounter microvascular resistance that demand significant plasticity for successful extravasation and colonization.^246^ The cumulative effect of these mechanical forces on tumor cells underscores a crucial aspect of metastasis, emphasizing the need for a biomechanical perspective in cancer research.

### Breaching extracellular matrix barriers

A decisive hallmark of malignancy is that they breach barriers of several ECM structures, such as the sheet-like basement membrane which separates the epithelium from the neighboring connective tissue.^247^ To penetrate the dense ECM network of the BM, cancer cells secrete several proteases of different types. Among the various proteases, matrix metalloproteinases (MMPs) are of outstanding importance, as they considerably affect the integrity of the ECM, the phenotype, and behavior of matrix-embedded cells and tissue turnover by degrading ECM proteins and selectively releasing cell surface-bound cytokines, growth factors, or their receptors.^248^ Of particular importance is the Zn^2+^ ion-dependent matrix metalloproteinase-14, which is usually found at a low level in normal cells but can reach higher levels in cancer cells. It is the only membrane-bound collagenase and can also activate other MMPs, such as the soluble gelatinases MMP-2 and MMP-9 as well as the collagenolytic MMP-13.^249^ Furthermore, proteolytic processing induced by MMPs activates Gα12/13 of the heterotrimeric G-protein and thereby Rho signaling. This affects the actomyosin machinery with its motor protein myosin II and thus increases cell contractility and cell movement. This further promotes invasion through the ECM barrier. Moreover, Rho activation promotes EMT of cadherin-interlinked cancer cells.^250^ In addition, cancer cells may also invade the BM through MMP-independent manners. For instance, CAFs exert mechanical forces on the BM by pulling the ECM fibers and stretching pre-existing gaps. The disrupted organization of the ECM results in a decreased BM stiffness and integrity, thereby reducing its barrier functions and making it more permissive for invasion.^251^ The alterations in composition and cross-linkage of ECM, dictated by the cancer cells or mostly carried out by CAFs, result not only in biochemical properties and storage of growth factors, but also change biophysical parameters, such as stiffness of the tumor stroma.^252^ Stiffness regulates malignant transformation by enhancing integrin signaling, which, in turn, promotes focal adhesions and activates ERK pathway that is for cancer proliferation, survival, migration and invasion.^253^

### Hemodynamic shear stress

Hemodynamic shear stress, the frictional force per unit area exerted by blood flow on the vascular endothelium, plays a crucial role in the pathophysiology of cancer metastasis. As tumor cells intravasate into the circulation, they encounter varying magnitudes of shear stress, which can profoundly influence their survival, adhesion, and extravasation into distant tissues.^254^ Recent studies have begun to unravel the intricate relationship between shear stress and tumor metastasis. For instance, Ma et al. demonstrated in their seminal work that hemodynamic shear stress facilitated the migration of tumor cells and enhanced their extravasation. They attributed this phenomenon to the upregulation of intracellular reactive oxygen species (ROS) levels, which activate the extracellular signal-regulated kinases (ERK1/2).^255^ Conversely, high shear stress may prevent the adhesion of CTCs, thereby reducing their extravasation at microvascular bifurcations.^256^ However, this force is not merely a passive barrier but a selective pressure that shapes the metastatic potential of CTCs. The small subset of CTCs that survive this mechanical stress exhibit adaptations that confer a survival advantage, including alterations in cell stiffness, cytoskeletal organization, and surface molecule expression.^257^ These adaptations enhance the cells’ ability to withstand further mechanical challenges, facilitating their survival and eventual colonization of distant sites. Additionally, previous research has indicated that shear stress promotes the formation of platelet-tumor cell aggregates, which in turn shields CTCs from shear stress. This process also decreases the exposure of CTC surfaces, aiding in their evasion of NK cell attacks.^258^

### Microvascular resistance

During the migration of tumor cells through the vasculature, they are confronted with diverse pressures, particularly within the confined spaces of capillaries. This mechanical challenge necessitates a remarkable level of cellular plasticity, compelling them to undergo deformation and compression in order to navigate through narrow passages. Such deformations not only impose physical stress but also act as triggers for cellular responses that facilitate survival and enhance metastatic potential.^259^ While transiting capillary constrictions, the cytoplasm of cells is prone to deformation, activating various mechanosensors that convert mechanical forces into chemical signals. These signaling proteins play a crucial role in determining the cell fate within CTCs, thereby enhancing metastasis in response to the cell deformation experienced within capillary beds. For instance, it has been reported that mechanical squeezing of cells in microfluidic and animal capillary models, such as in mice and zebrafish, increases the activity of RhoA, thereby leading to the recruitment and activation of ROCK. When CTCs are lodged within capillaries, the subsequently activated RhoA-ROCK-myosin II axis enhances contractile forces, causing CTCs to transition from an elongated to a spherical phenotype. This cellular rounding facilitates the capture of CTCs and their subsequent extravasation.^260,261^ In addition, nuclear deformation has been identified as a primary spatial constraint for cell migration in confined spaces.^262^ However, under capillary-induced constriction, the cell nucleus experiences myosin contractions that push or pull, aiding in cell displacement.^263^ Furthermore, mislocalization of repair factors observed in constricted cells may lead to an increase in DNA damage, potentially resulting in chromosomal instability, a hallmark of metastatic cancer.^264^ Despite the limited research on the role of microvascular resistance in tumor metastasis, the observation that CTCs rapidly enter circulation but persist within capillary beds for extended periods before extravasation implies that investigating CTC adaptations within capillaries may present a promising strategy to impede metastatic progression.

## Treatment of cancer metastasis

### Traditional treatment

Contemporary cancer management frequently employs comprehensive treatment modalities such as surgery, chemotherapy, and radiotherapy. While surgery and radiotherapy serve primarily as regional interventions, chemotherapy functions as a systemic remedy, exerting a cytotoxic effect on disseminated cancer cells and those migrating into the blood and lymph systems.

Surgical removal of primary tumors or metastases remains foundational in treatment, with potential benefits in prolonging survival and/or enhancing quality of life. In patients with confined metastasis, surgical excision has evidenced improved disease control and survival, especially in cases like breast cancer with isolated bone metastases.^265,266^ A notable risk for metastatic recurrence exists post-surgical resection of primary tumors, with an observed peak in recurrence 12–18 months following surgery in breast cancer, for instance.^267^ The hypothesis posits that surgical intervention might inadvertently activate dormant metastases, thus leading to synchronous relapse patterns. Induced systemic inflammatory responses post-surgery may stimulate malignant proliferation, with studies indicating that perioperative anti-inflammatory treatment might significantly attenuate tumor growth, suggesting a potential therapeutic avenue for anti-metastasis.^268^ Pre-operative (neoadjuvant) or post-operative (adjuvant) therapeutic interventions can enhance the efficacy of surgical measures.

Chemotherapy stands as a cornerstone for systemic intervention. It primarily addresses tumors predisposed to widespread dissemination or those that have already metastasized. While extending survival and alleviating symptoms, chemotherapy seldom cures or eliminates metastases. In the context of metastatic breast cancer, particularly for the triple-negative subtype, chemotherapy remains the sole systemic therapeutic option in the absence of viable targeted treatments, aiming to thwart tumor regrowth by targeting rapidly dividing cells.^269^ Nonetheless, murine studies suggest a pro-metastatic facet to chemotherapy, potentially as primary tumors release EVs that foster metastasis in distant organs.^270^ Administered before local treatments, neoadjuvant chemotherapy aims to minimize tumor size and eradicate early metastatic cells, thereby enhancing subsequent treatments. Research utilizing single-cell analysis of CRC liver metastases pinpointed hyper-metabolically active M2-like macrophages at metastatic sites. Effective neoadjuvant chemotherapy may modulate this metabolic surge, orienting treatment strategies towards metabolic intervention in metastatic cancer.^165^

Radiotherapy, employing targeted radiation to eliminate tumors, remains a pivotal local treatment modality. The integration of positron emission tomography (PET) in clinical diagnostics refines the biological delineation of tumors, aiding in deciphering potential treatment resistance.^271^ Innovations in diagnostic imaging have ushered in refined therapeutic tactics, sharpening the demarcation between tumor and healthy tissues. With stereotactic radiosurgery and stereotactic ablative radiotherapy (or stereotactic body radiation therapy), pinpoint delivery of radiation therapy is achievable. In NSCLC cases, employing stereotactic ablative radiotherapy (SABR) before maintenance chemotherapy proved advantageous, enhancing progression-free survival (PFS) markedly when compared with standalone maintenance chemotherapy.^272^ Contemporary evidence underscores the potential of radiation to amplify the immune response elicited by checkpoint inhibitors.^273,274^ Research substantiates radiation’s capability to augment the genesis of antigen-specific immune reactions.^275^ Hence, amalgamating radiation with immunotherapy presents a promising avenue. The synergy between immunotherapy and radiotherapy could redefine the treatment benchmark for numerous cancers. Yet, it’s crucial to recognize that irradiation might mobilize circulating tumor cells and inadvertently stimulate tumor cell dissemination, fostering subsequent metastatic development.^276^

### Targeted therapy

While cytotoxic chemotherapy continues to be the primary treatment modality for metastasis and remains the sole option for several cancer subtypes, the advent of drugs targeting specific tumor proteins, termed “targeted therapies,” offers new avenues for numerous metastatic tumors. Predominantly, these targeted agents are either small molecule drugs or mAbs. Due to their diminutive size, small molecule inhibitors have the capability to interact with a broader array of both intracellular and extracellular targets, rendering a distinction in selectivity from antigens. The formulation of these inhibitors generally adheres to two distinct trajectories: multi-kinase inhibitors and selective inhibitors. For refractory or metastatic cases, both multi-target and selective kinase inhibitors are utilized, especially for advanced malignancies resistant to other treatments.^277^ Notably, in the context of metastatic breast cancer, prevalent small molecule targeted therapeutic strategies encompass CDK4 and CDK6 inhibitors, PARP inhibitors, and PI3K inhibitors.^278^ For patients overexpressing HER2, the platform employing a recombinant protein (RP-HI) and its drug conjugates (RPDC-HI) emerges as a promising approach, delivering synergistic therapeutic outcomes, especially against tumors resistant to conventional HER2-targeted therapies.^279^

Monoclonal antibodies (mAb) are tailored proteins designed to recognize and bind to specific markers on cancer cell surfaces. The FDA has sanctioned mAb-based targeted therapy for varied cancer types, with certain combinations demonstrating enhanced efficacy. As illustrative cases, pertuzumab and trastuzumab are mAbs targeting HER2, a protein found in abundance in particular breast cancer and CRC. These antibodies can be employed synergistically with agents like docetaxel or lapatinib, bolstering survival and treatment response in patients with HER2-positive metastatic malignancies.^280,281^ Tumor cells have been identified to express complement inhibitors, a potential mechanism that permits evasion from mAb interventions and potentially modulates the evolution of acquired immune responses against tumors. Recent research has explored methods to augment mAb-induced complement activation on tumor cells. Such an approach, as evidenced in a concomitant model of metastatic lymphoma (EL4) and melanoma (B16), significantly enhanced the therapeutic efficacy of mAb.^282^ The combined utilization of therapies can potentiate the mode of drug action, mitigate resistance, and extend the benefits seen with monotherapies. Yet, it is pertinent to note that despite rigorous endeavors in integrating small molecule targeted inhibitors, a limited number of combined therapeutic strategies have transitioned into clinical application.

Radioactive particle irradiation therapy also stands out as a potent targeted intervention for metastatic malignancies. Targeted alpha therapy is a novel treatment modality. Given the selectivity of alpha particles, systemic radiation is specifically delivered to cancer cells. Their short-range emission ensures that cytotoxicity is localized to the cancerous lesions and the adjacent TME, thereby minimizing deleterious effects on healthy tissues.^283^ A radiolabeled molecule, Lutetium-177 [Lu]-PSMA-617, demonstrates high affinity to prostate-specific membrane antigen (PSMA). This beta particle therapy is adept at targeting metastatic castration-resistant prostate cancer, manifesting efficacy with diminished adverse effects and improving survival in affected men.^284,285^ Subsequent to therapy, a notable reduction in PSMA-positive circulating tumor cells offers substantial implications for both patient stratification and continuous disease monitoring.^286^

Current phase III trials are examining the clinical efficacy of both ‘oncogene’ and ‘non-oncogene’ targeted agents in conjunction with immune checkpoint inhibitors. ‘Oncogene’ pertains to multiple oncogenic pathways essential for sustained tumor progression. Conversely, ‘non-oncogene’ signifies stress response pathways indispensable for transformed cell survival; while these pathways cannot instigate tumorigenesis, they remain crucial. These pathways not only afford malignant cells a growth or survival advantage, but also foster an immunologically “cold” tumor microenvironment to buttress tumor progression.^287^ Thus, targeting both ‘oncogene’ and ‘non-oncogene addiction’ is anticipated to rejuvenate the TME and amplify the ICI response.

The pivotal role of the TME in modulating metastasis has been increasingly recognized, rendering it a prime therapeutic target. Tumors rely on blood to deliver essential oxygen and nutrients, making tumor neovascularization an early therapeutic focus within the TME. The efficacy of anti-VEGF tyrosine kinase inhibitors (TKIs) and the anti-VEGF antibody bevacizumab in metastasis across diverse malignancies has been substantiated.^288^ Additionally, tumor-associated mesenchymal stem/stromal cells (TA-MSCs) in the TME emerge as potent targets. By secreting growth factors, chemokines, and cytokines, TA-MSCs influence tumor growth, metastasis, and treatment responses. Strategies targeting the TA-MSC-secreted factors, which enhance tumor growth, metastasis, and drug resistance, are being developed.^289^ The TME harbors numerous cells that can be envisaged as prospective therapeutic targets. Given that targeted therapy modulates the TME’s immunological components, it’s imperative to not only consider the optimal sequencing or amalgamation of ICIs and targeted therapy but also assess how TME’s stromal cells influence the efficacy of other interventions.^290,291^

Photodynamic targeting therapy operates based on cellular functions or morphological alterations, triggered by light in the presence of photosensitizers, leading in extreme circumstances to cell devastation and necrosis. Near-infrared (NIR) photoimmunotherapy (PIT) is a cancer phototherapy targeting specific TME cells, such as regulatory T cells. This boosts selective systemic tumor cell immunity, inducing potent responses in distant metastatic tumors. By integrating cancer-targeted NIR-PIT with immune-activating NIR-PIT or other immunotherapies, localized tumor NIR-PIT can provoke responses in distant metastatic tumors, simultaneously suppressing recurrence, rendering it a potent therapeutic strategy for metastatic cancer.^292^

Moreover, organ-specific metastasis targeting can enhance patient prognosis. Due to varied reasons, some cancers predominantly metastasize to a specific organ, undergoing dormancy before spreading further.^293^ For instance, CRC metastasizing to the liver, largely attributed to anatomical and circulatory factors, provides a paradigm. Surgical resection of minimal CRC liver metastases is standard, offering curative outcomes in approximately 20% of patients.^294^ Bone often represents an initial metastatic site for breast and prostate cancers. Radium-223, an alpha emitter, selectively targets bone metastases, enhancing overall survival rates for bone metastatic prostate cancer patients.^295^ For small cell lung cancer, the brain is a consistent metastatic site, and thus, prophylactic cranial irradiation augments overall survival.^296^ The meningeal space and abdominal cavity, sites detached from circulation or possessing immunosuppressive microenvironments, are also metastatic regions. A deeper comprehension of metastatic mechanisms and their targeting can potentially refine therapeutic outcomes for cancers infiltrating these locations.^297,298^

### Cancer immunotherapies

Cancer treatment traditionally relies on the triad of surgery, radiotherapy, and chemotherapy. Recent advancements have ushered in cancer immunotherapies, which offer a model for treatment that minimizes toxic and adverse side effects. The incorporation of immunotherapy as a pivotal modality in cancer treatment is a recent development. Immunotherapy functions by augmenting, activating, and proliferating a patient’s immune cells, thereby modulating tumor growth, refining the TME, adjusting immune system functionality, altering cytokine levels to restrain tumor progression, and prompting cancer cell apoptosis.

### Immune checkpoint inhibitors

Immune checkpoints serve as regulatory pathways essential for maintaining the amplitude and duration of immune responses. Tumors exploit these pathways to evade immune attack. The advent of cancer immunotherapies targeting adaptive immune checkpoints has enhanced the prognosis for patients with diverse metastatic and refractory cancers. Novel therapeutic interventions include those targeting T-cell checkpoint receptors: cytotoxic T-lymphocyte-associated protein 4 (CTLA-4), programmed cell death 1 (PD1), and programmed cell death 1 ligand (PD-L1). These therapies have improved clinical outcomes for numerous solid tumors. Immune checkpoint inhibitors can disrupt tumor-resistant immune mechanisms, amplifying the body’s immune response against tumor cells. The FDA has approved various ICIs for the treatment of ten distinct cancer types, including CTLA-4 mAbs like Ipilimumab, PD1 monoclonal antibodies such as Nivolumab and Pembrolizumab, and PD-L1 monoclonal antibodies like Atezolizumab and Avelumab. CTLA4 and PD1, operating at distinct sites and phases within the immune system, function in a complementary and supplementary manner, ensuring T-lymphocyte responses adequately defend against tumor cells while sustaining levels conducive to self-tolerance. The synergistic administration of the anti-PD-L1 agent durvalumab with the anti-CTLA-4 drug tremelimumab has shown efficacy for advanced or metastatic sarcoma, warranting further investigation in subsequent trials.^299^ In metastatic breast cancer management, when the primary tumor is retained and surgically excised post-ICI administration, observations indicate amplified tumor-specific T-cell expansion, augmented micro metastases elimination, and extended survival compared to adjuvant ICI post primary tumor excision.^293^ Some metastatic cancers, due to an elevated tumor mutational burden, generate an abundance of mutant peptides. These peptides, presented on MHC-I molecules on tumor cell surfaces, are discerned as foreign neoantigens by the immune system, facilitating ICI functionality.^293^ Presently, only a subset of cancer patients, including those with metastatic melanoma, lung, bladder, or kidney cancers, and cancers with mismatch repair system anomalies, benefit from ICIs. Concurrent research indicates that innate immune checkpoints, which impede the recognition and elimination of malignant cells via phagocytosis and inhibit innate immune sensing, play a pivotal role in tumor-induced immune evasion, positioning them as potential targets for cancer immunotherapy.^300^

In addition to the direct suppression or depletion of immunosuppressive lymphocytes and myeloid cells, conventional oncological interventions have been identified as potential complements to ICIs, yielding enhanced outcomes. Atezolizumab-mediated cancer cell death may be potentiated by bevacizumab, which obstructs vascular endothelial growth factor-mediated immunosuppression. A study revealed that the combination of bevacizumab, atezolizumab, and chemotherapy significantly improved overall survival and PFS in patients with metastatic non-squamous lung cancer.^301^ The advent of ICI-based immunotherapy marks a novel era in tumor treatment, and the identification of predictive biomarkers is pivotal for the advancement of precision immunotherapy. Enhanced comprehension of the biology and molecular subtypes of non-small cell lung cancer, coupled with biomarker-driven immunotherapeutic approaches, has prolonged the overall survival of patients with metastatic non-small cell lung cancer.^302^ Evidence suggests that integrating Mn with immune checkpoint inhibition can augment anti-tumor efficacy and diminish the requisite dosage of anti-PD1 antibody. A completed phase 1 clinical trial demonstrated that a combination regimen of Mn and anti-PD1 antibodies exhibited efficacy in the majority of patients with advanced metastatic solid tumors, eliciting type I IFN induction, a manageable safety profile, and a rejuvenated response to immunotherapy.^303^

Nevertheless, ICIs can enhance immune responses, potentially disrupting the body’s inherent immune tolerance.^304^ Of the patients treated with CTLA4 and PD1 ICIs, 30% and 15%, respectively, encountered severe events necessitating immediate intervention.^305^ A common consequence of immune checkpoint inhibitor therapy is the proliferation of hyper-activated memory T lymphocytes and the diminution of T lymphocyte precursor cells. Hyperactive T lymphocytes can infiltrate the gastrointestinal tract, lungs, and other organs, inflicting immunopathological damage to the body’s normal cells.^306^ Gastrointestinal complications and neurological toxicities are prevalent with anti-CTLA4 therapies, whereas therapies targeting the PD1 axis are more inclined to induce hypothyroidism, hepatotoxicity, and pneumonia.^307^ Further investigations are essential to comprehensively address the side effects engendered by ICIs in clinical settings.

### NK cells therapy

NK cells are cytotoxic lymphocytes, a distinct and meticulously modulated subset of innate lymphocytes (ILC). Upon activation, they swiftly respond to viral infections or tumorigenesis, initiating the apoptosis of the infected or tumorous cells within the bloodstream. However, NK cells devoid of the E3 ubiquitin ligase Cbl-b (casitas B-lineage lymphoma-b) are intrinsically resistant to tumors, and recent studies have highlighted their potential in mitigating tumor metastasis.^308^ NK cells can inhibit tumor cell migration and invasion by secreting cytokines, notably IFN-γ, thus maintaining tumor dormancy.^309^ Despite substantial phenotypic, biochemical, and metabolic shifts associated with metastasis, treatments effective for primary tumors, especially those targeting driver mutations, often lack substantial anti-metastatic properties. The underlying mechanism for NK cells’ apparent predilection towards controlling metastatic spread remains undetermined, but it is evident that they play a role in metastatic progression.^310^ Broadly, NK cells provide swift and potent immunity against metastatic malignancies. Various strategies encompass the development of extensive NK cell expansion protocols for adoptive transfer, establishing a microenvironment conducive to NK cell activity, redirecting NK cell activity towards tumor cells, and alleviating inhibitory signals that constrain NK cell functionality. These initiatives have facilitated the comprehensive implementation of NK cell immunotherapy in managing metastatic cancer.^311^

### Adoptive cell transfer (ACT)-based immunotherapy

ACT-based immunotherapy offers a novel method for treating metastatic cancer. This form of immunotherapy is tailored to individual patients. By inducing differentiation, modification, and expansion of tumor-associated antigen-specific T cells in vitro, large quantities of these cells can be produced. These cells, when transferred to patients with tumors, can suppress and eradicate tumor cells. The utilization of naturally occurring or genetically engineered T lymphocytes in ACT holds potential therapeutic benefits for those with metastatic cancers.^312^ Current clinical approaches prominently feature tumor-infiltrating lymphocytes (TILs), chimeric antigen receptor T cells (CAR-T), and T cell receptor-modified T cells (TCR-T). While TILs and TCR-T therapies can target a spectrum of both extracellular and intracellular antigens and maintain prolonged in vivo activity, their clinical application is constrained by their moderate antitumor efficacy and notable side effects. CAR-T cells, equipped with the dual capability of antigen specificity and cellular activation, can selectively eliminate tumor cells. However, further evidence is required to substantiate their therapeutic efficacy. The genetic modification of autologous lymphocytes in ACT to amplify their antitumor potency presents promising avenues for future cancer treatments.^313^

Both CAR-T and TCR-T, representing advanced modalities in ACT, employ autologous lymphocytes to target various cancer forms.^313^ Notably, ACT has achieved complete, sustained remissions in as many as 40% of treated patients. A significant challenge for CAR-T cells, especially in solid tumors, is the immunosuppressive tumor microenvironment, marked by elevated concentrations of inhibitory factors, including TGF-β.^86^ The genetic stability of stromal cells within the TME, contrasted with the recognized genetic heterogeneity in tumor cells,^314,315^ underscores the TME as a potential therapeutic target.

TIL therapy represents an emerging form of cell-based immunotherapy. TILs serve as novel anti-tumor effector cells, establishing themselves as potential targets for immunotherapeutic interventions. The National Cancer Institute initiated ATC therapies utilizing TILs for the management of metastatic melanoma in the late 1980s.^316^ Nonetheless, the observed median response duration was a mere 4 months, with only a minority of patients achieving complete response.^317^ A subsequent trial incorporated lymphatic drainage prior to ATC administration in 93 metastatic melanoma patients, resulting in complete tumor regression in 22 individuals. Impressively, 19 of these patients maintained complete remission three years post-treatment.^318^ Due to the intricate nature of the tumor microenvironment, TILs demonstrate limited tumor eradication capabilities. Thus, TILs are expanded in vitro and then reintroduced into the patient. From a theoretical standpoint, TIL therapy might be applicable even when tumors lack identifiable driver mutations, when CAR-T cells cannot infiltrate solid tumors, or when ICIs are ineffective in “cold” tumors. Highlighting its potential, a study demonstrated that metastatic cervical cancer patients achieved sustained complete remission following a single administration of HPV-TILs.^319^ Post-expansion, only a subset of TILs remains reactive to established tumor neoantigens upon reinfusion, presenting the challenge of enhancing the proportion of reactive TCR varieties. The therapeutic outcomes of TIL therapy are influenced by numerous parameters, including tumor characteristics, tumor burden, TIL source and quality, cell count, administration protocol, lymphatic drainage, and pre-treatment strategies. Consequently, treatment plans must be meticulously tailored to the individual requirements of each patient. Predominant adverse effects associated with ATC therapy encompass cytokine release syndrome (CRS) and neurotoxicity.^320^ Employing glucocorticoids or Tocilizumab might mitigate these negative consequences.^321^ Nevertheless, the intricate preparatory procedures and substantial costs pose significant challenges to the broad clinical application of this method.

### Tumor vaccine

Tumor vaccines aim to elicit tumor-specific immune responses by utilizing tumor-associated antigens, tumor peptides, or tumor cell lysis products, offering both prophylactic and therapeutic potentials against cancers. Therapeutic tumor vaccines seek to eliminate tumors by inciting the immune system to produce specific antibodies, effector cells, and immune memory cells. There exists an approach to personalize tumor vaccines that stimulates a synergistic response from varied T cell and NK cell populations. Particularly in cases of highly metastatic cancers, post-surgical immunization with this vaccine can deter the emergence of subsequent metastases. Such vaccines can also be tailored to confer immunity against tumors that exhibit prevalent escape mutations.^322^ Research involving metastatic melanoma, non-small cell lung cancer, and bladder cancer patients suggested enhanced efficacy of nivolumab when co-administered with a vaccine targeting tumor neoantigens, yielding extended progression-free survival periods.^323^

For an optimized anti-tumor effect, immunotherapy can be amalgamated with other treatments. For instance, low-dose radiotherapy has demonstrated the ability to recalibrate the tumor microenvironment to boost immune responses, thereby achieving tumor control.^324^ This strategy proves especially beneficial in treating metastatic tumors that traditionally exhibit low immune infiltration. Another potent combination involves uniting immunotherapy with chemotherapy. The pairing of trastuzumab with lapatinib has been deemed effective and exhibits a tolerable profile in patients diagnosed with treatment-resistant, HER2-positive metastatic CRC.^280^ Furthermore, the introduction of pembrolizumab to a standard chemotherapy regimen, comprising pemetrexed and a platinum-based agent, has shown superior overall and progression-free survival results in previously untreated metastatic non-squamous NSCLC patients without EGFR or ALK mutations.^325^ Given the scarcity of tumor-infiltrating lymphocytes in a majority of breast cancers, monoclonal antibody-based monotherapies have underperformed in treating metastatic breast cancer patients.^293^ However, augmenting trastuzumab-based chemotherapy with pertuzumab has clinically demonstrated a significant enhancement in overall survival for patients with ERBB2-positive metastatic breast cancer.^326^ Moreover, in the surgical management of prostate cancer, modulating the excessive perioperative stress-inflammatory response through pharmacological means, in conjunction with immune stimulation, can mitigate surgery-induced metastatic tendencies, counteract immunosuppression, and elevate the immune response.^327^

In comparison to adjuvant therapy, neoadjuvant therapy has exhibited a higher efficacy in eradicating distant metastases following primary tumor excision. This superior efficacy is attributed to the heightened and sustained peripheral tumor-specific immune response elicited by neoadjuvant therapy. Specifically, neoadjuvant therapy’s capacity to elevate the proliferation and production of IFN-γ and TNF by gp70 tumor-specific T lymphocytes in peripheral blood and various organs shortly post-treatment might be pivotal to its benefits.^328^ Collectively, the potential of immunotherapy in cancer management is profound.

### Cytokine therapeutics

Cytokines and their associated receptors have been rigorously investigated as potential therapeutic targets for tumors, with applications in cancer treatment spanning over four decades. Serving as regulators of cellular activity, cytokines are produced by various cell types and play a pivotal role in signaling within the TME. There is robust preclinical evidence advocating therapeutic strategies that augment the growth inhibitory and immunostimulatory effects of interferons and interleukins, notably IL-2, IL-7, IL-12, and IL-15, and strategies that neutralize the pro-inflammatory and tumor-promoting effects of cytokines such as TNF, IL-1, and IL-6.^329^ The advancement in immunotherapeutic approaches and deeper insights into the TME have paved the way for novel methods to harness cytokine networks in cancer therapy.

The 1983 cloning of the IL-2 gene marked a significant breakthrough, propelling the development of IL-2 drugs. A collaborative effort between Novartis and Linigen culminated in the production of Proleukin® (aldesleukin), a recombinant IL-2 medication, which subsequently received FDA approval for treating metastatic kidney cancer and metastatic melanoma.^330^ The cytokines of the IL-2 family, specifically IL-2, IL-7, IL-15, and IL-21, govern the proliferation, maturation, and cytotoxic functions of innate immune cells. These cytokines utilize heteromeric receptors sharing a common γc receptor subunit. Nevertheless, systemic administration of IL-2, being a non-targeted therapeutic approach, has been associated with severe adverse effects, including hypotension and capillary leakage syndrome, thereby constraining its widespread clinical utilization.^331^ Consequently, strategies that merge IL-2 with antibodies, directing it to the lesion site, have been explored to mitigate these side effects. Specifically targeting tumor sites, cytokines such as IL-2, IL-12, IL-21, TNF-α, and IFN-α, -β, and -γ have demonstrated antitumor responses without the systemic side effects characteristic of free cytokine administration.^332^ IL-21, which amplifies the cytotoxic actions of CD8^+^ T cells and NK cells, has been assessed as a monotherapy in phase I/II clinical trials for metastatic CRC.^333^ Within the TME, cytokines like TGF-β, CXCL4/12, IL-6, and TNF-α can potentiate EMT, leading to heightened metastatic potential and chemoresistance. In particular, TGF-β has been implicated in facilitating the metastasis of various cancers, including breast, lung, gastric, and prostate, to tissues such as bone, liver, and lungs.^334^ Neutralizing mAb or receptor antagonists targeting these inflammatory cytokines have exhibited anticancer properties. They are now being combined with other immunotherapies and oncological treatments in clinical trials. Notably, various TβRI kinase inhibitors are under clinical evaluation for cancer treatment. For instance, the combination of the TβRI kinase inhibitor SM16 and the agonistic OX40 antibody significantly curtailed metastasis.^335^ SD208, a TβRI inhibitor, impedes the TGF-β/SMAD pathway, matrix invasion, and expression of target genes, showing efficacy against established osteolytic lesions and prevention of bone metastases in specific cancer models.^336,337^ Research suggests that a combination of a TGF-β inhibitor and a PD-L1 inhibitor (Atezolizumab) can modify the matrix microenvironment, facilitating T cell penetration into the tumor.^338^ Furthermore, in the context of chimeric antigen receptor (CAR) T-cell therapy, CAR-T cells have shown the potential to counteract the immunosuppressive effects of TGF-β, underscoring the potential of combination therapies in future oncological strategies.^339^

Cytokines are integral to the functions of both the innate and adaptive immune systems. Though there have been attempts to employ systemic monotherapy with cytokines for cancer treatment, this strategy presents inherent challenges that need addressing for cytokines to have a primary role in cancer immunotherapy. One innovative solution is the synthesis of antibody-cytokine fusion proteins. By combining cytokines with antibodies that specifically target antigens on cancer cells, these fusion proteins can localize cytokine delivery to the tumor, thereby limiting systemic exposure and potential systemic side effects. An alternative approach employs genetic engineering to alter the cytokine molecule, as demonstrated with IL-2. Such methodologies mark considerable progress in cytokine-based cancer immunotherapy and suggest a potential path forward in deploying cytokines as effective therapeutic agents for cancer.

### Nanotechnology

Nanotechnology presents a potent therapeutic strategy against metastatic cancer. An essential component of metastatic cancer treatment is the ability to target metastatic sites, a feat not achieved by many current therapies. Nanomaterials, owing to their design, can target specific tissues and even specific subcellular regions. Notably, nanoparticles can transport intricate molecular cargo to crucial metastatic sites, such as the lymph nodes, liver, and lungs, and focus on specific cellular subsets within these organs. While the majority of nanotechnological interventions in oncology are directed towards primary tumors, there’s an emerging focus on leveraging nanotechnology to inhibit cancer’s metastatic process at every stage. This includes stages like angiogenesis, intravasation, tumor cell circulation, extravasation, and growth in secondary sites.^340^ Nanotechnology achieves this through various mechanisms.

Each phase of the metastatic cascade is crucial for metastasis formation. Targeting the immune cells implicated in this cascade is pivotal for the management of metastatic cancer. Given the potential of nanomaterials (and their drug cargo) to modulate immune responses, nanomedical strategies targeting immune cells are drawing significant attention.^341^ Nanoparticles can initiate tumor apoptosis via multiple mechanisms, including direct induction of tumor cell death, dendritic cell-triggered innate immune responses, and T cell-mediated adaptive immune responses.^342^ For instance, cyclic dinucleotide (CDN) agonists of the stimulator of interferon genes (STING) are emerging as potent immunotherapeutics. Specifically, STING-activating nanoparticles (STING-NPs), engineered polymersomes, enhance the bioactivity of the endogenous CDN ligand for STING, known as 2'3’ cyclic guanosine monophosphate-adenosine monophosphate (cGAMP). This enhancement results in tumor growth inhibition and increased long-term survival rates.^343^ Furthermore, a specially designed supramolecular cationic gold nanorod can serve as a delivery mechanism for CRISPR/Cas9 targeting PD-L1, amplifying the efficacy of immune checkpoint blockade (ICB).^344^ Nanobubbles (NBs) are also being recognized as potential enhancers in cancer immunotherapy.^345^

Cancer nanotheranostics integrates imaging and therapeutic modalities using nanotechnology. Many contrast agents utilized in computed tomography are small molecules with brief in vivo half-lives.^346^ Encapsulating these agents in nanoparticles can prolong their residence time, potentially reducing the necessary dosage.^346^ For patients with metastatic cancer, this might decrease toxicity while enhancing the specificity and signal intensity of the imaging agent, facilitating the visualization of metastases during surgical procedures.^340^ Nanomaterials assist in surgical resection, identify circulating tumor cells, and pinpoint specific tumor subregions.^340^ Additionally, nanomaterials have potential applications in phototherapy. In a study involving a 4T1-tumor-bearing mouse model, nanocomposites demonstrated impressive efficacy in photodynamic therapy and photothermal conversion under wavelengths of 650 and 808 nm, leading to significant tumor growth inhibition.^347^ Furthermore, the efficacy of chemotherapy can be augmented through the development of innovative nanomedicines based on mesoporous silica nanoparticles (MSNs).^348^

The treatment of cancer faces several challenges, including the lethal metastasis of cancer cells, multi-drug resistance to conventional chemotherapeutic drugs, and significant toxic side effects on healthy tissues due to a lack of tumor selectivity. MSNs hold the potential to address these challenges. Recent advancements in micro/nanorobotic (MNR)-assisted diagnostics and treatments have paved the way for MNR-based therapeutic strategies in oncology. DNA-based nanorobotic systems have been designed to deliver agents with distinct biological activities, which are not amenable to traditional therapeutic delivery, for targeted tumor therapy.^349^ Emulating the coagulation cascade, a nanoparticle-mediated strategy was devised for selective tumor vascular infarction. This approach entails the specific delivery of thrombin-loaded DNA nanorobots (Nanorobot-Th) to tumor vasculature, inducing vascular infarction, intravascular thrombosis, and consequently, tumor necrosis.^350,351^

### Oncolytic virotherapy

Oncolytic virotherapy represents a novel approach to antitumor treatment, utilizing viruses that preferentially replicate within and destroy tumor cells, thereby bolstering antitumor immunity. Oncolytic viruses (OVs) offer therapeutic potential due to their ability to selectively target and lyse cancer cells while sparing normal tissues. The tissue specificity of each virus enhances its therapeutic efficacy.^352^ Capable of systemic or locoregional administration, OVs, as replicating biotherapeutics, exert immunological effects on both primary and metastatic tumor sites, suggesting their potential in treating metastatic malignancies. Notably, the FDA has endorsed talimogene laherparepvec (Imlygic) as the first OV for the treatment of metastatic melanoma.^353,354^ OVs can either operate as independent immunotherapeutic entities or as platforms integrated with other agents, such as immune checkpoint inhibitors, tumor antigens, cytokines, or T-cell engagers, to modulate the immune response.^355,356^

The integration of OVs with ACT offers promising therapeutic avenues. OVs can enhance ACT efficacy by modulating the systemic immune landscape and tailoring the tumor microenvironment to support T cell activity.^356^ By optimizing immune-mediated tumor elimination, OVs can transform both localized and disseminated tumor sites.^357^ Recent evidence suggests that the concomitant use of OVs and ACT involving tumor-specific T cells yields superior outcomes compared to either monotherapy due to enhanced T-cell effector functions.^358^ In a study, an oncolytic adenovirus was engineered to deliver the immune-stimulatory cytokine IL-12 and a PD-L1-blocking antibody, and was subsequently combined with HER2-specific CAR-T cells.^359^ The combined therapy demonstrated enhanced control over primary and metastatic tumor growth, leading to substantially extended survival when compared to individual treatments. Several collaborative mechanisms can amplify the impact of OVs, such as employing cellular vectors for optimized viral delivery or utilizing immunosuppressive agents to facilitate intratumoral viral propagation.^352^ Furthermore, OVs can transform newly developed tumors into in situ neoantigen reservoirs via cross-presentation, resulting in the regression of remote, non-infected tumors. This capability underscores the potential of OVs as part of a combined approach to maximize cancer immunotherapy outcomes.^360^

### Antibody-drug conjugates (ADCs)

ADCs are designed to enhance the efficacy of the therapeutic payload while minimizing its systemic toxicity, in contrast to traditional cytotoxic agents. Following internalization, ADCs are processed in lysosomes, culminating in the release of their payload. This payload then exerts its cytotoxic action, typically via microtubule disruption or DNA damage, leading to cell death.^361^ The targeted delivery facilitated by ADCs elevates the proportion of the drug that reaches the tumor cells. This potentially reduces the necessary effective dose and heightens the maximum dose that can be tolerated. Nonetheless, continuous advancements are required in antibody selection, linker technologies, payloads, and conjugation methods, especially to address concerns like off-target toxicities stemming from suboptimal linker stability.

**Fig. 8:**
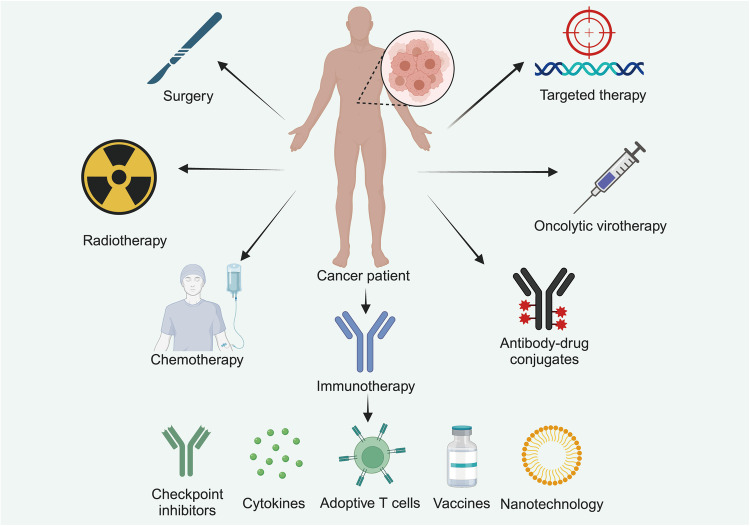
There are many options for cancer treatment. The type and stage of cancer you suffer from will determine the type of treatment you receive. Some cancer patients only receive one type of treatment. However, most patients use combination and personalized treatment, such as surgery combined with chemotherapy and/or radiation therapy. The emergence of targeted therapy and immunotherapy, including immune checkpoint inhibitors, cell therapy, adoptive cell transfer (ACT) based immunotherapy, tumor vaccines, and nanotechnology, has completely changed the treatment of cancer. At the same time, some emerging therapies, such as oncolytic virus therapy and antibody drug coupling therapy, are working towards cancer treatment. Created with BioRender.com

In the context of metastatic cancers that are HER2-negative, such as endocrine-resistant, HER2-negative, and hormone receptor-positive (HR^+^) metastatic breast cancer, single-agent chemotherapy has traditionally been employed, typically yielding unsatisfactory results. Sacituzumab govitecan (SG), an ADC, bears a 7-Ethyl-10-hydroxycamptothecin (SN-38) payload and targets the trophoblast cell-surface antigen 2, a notable epithelial antigen in breast cancer. Current data indicate that SG provides a statistically significant PFS advantage over traditional chemotherapy, positioning it as a potential therapeutic alternative.^362^ In metastatic cancers that are HER2-positive, such as HER2-positive breast cancer, trastuzumab deruxtecan, another ADC, has demonstrated efficacy in patients with metastatic HER2-positive breast cancer who have experienced disease progression post-multiple treatments. Notably, trastuzumab deruxtecan exhibited a significant intracranial response in patients with metastatic HER2-positive breast cancer presenting with newly identified untreated brain metastases or brain metastases that had progressed after initial local treatments, suggesting its potential role as a treatment avenue.^363^ Furthermore, trastuzumab deruxtecan has also exhibited sustained and notable efficacy in patients with HER2-positive metastatic CRC unresponsive to standard regimens. A comprehensive overview of these findings can be referenced in Tables 3, 4 (Fig. 8).

**Table 3:** Typical treatment for metastatic cancer

Treatment	Cancer	Result	Reference number
Chemotherapy	Metastatic breast cancer	Chemotherapy is designed to prevent tumor regrowth by killing actively dividing cells, and are the only systemic treatment option currently available for the triple-negative subtype of tumors.	^269^
Radiotherapy	Non-Small Cell Lung Carcinoma	In NSCLC, stereotactic ablative radiotherapy (SABR) prior to maintenance chemotherapy appeared beneficial, nearly tripling progression-free survival in patients compared with maintenance chemotherapy alone.	^272^
Radiotherapy and immunotherapy	Metastatic cancer	Radiation enhances many of the steps needed for the generation of antigen-specific immune responses, thus there is promise in combining radiotherapy with immunotherapy.	^275^
Targeted therapy	Metastatic breast cancer	There are standard therapeutic small molecule targeted drug approaches such as CDK4 and CDK6 inhibitors, PARP inhibitors, PI3K inhibitors.	^278^
Targeted therapy	HER2-positive metastatic breast cancer	The dual blockade of pertuzumab and trastuzumab, with docetaxel, brings good effects, even demonstrating an 8-year landmark overall survival rate of 37%.	^281^
Targeted therapy	HER2-positive metastatic colorectal cancer	The combination of trastuzumab and lapatinib is active and well-tolerated, and dual blockade of HER2 inhibits tumor growth in patient-derived in metastatic colorectal cancer.	^280^
Targeted therapy	Metastatic prostate cancer	A radio-labeled small molecule, Lutetium-177 [Lu]-PSMA-617, binds with high affinity to prostate-specific membrane antigen (PSMA), and the beta particle therapy can target metastatic destructive resistant prostate cancer.	^443^
Targeted therapy	Metastatic cancer	New anti-tumor growth and metastasis strategies are developed by targeting the factors produced by tumor-associated mesenchymal stem/stromal cells in TME.	^289^
Targeted therapy	Metastatic prostate cancer	Bone is often an early site of metastasis in prostate cancers, and alpha emitter radium-223 selectively attacks bone metastases with alpha particles, improving overall survival in patients with bone metastatic prostate cancer.	^295^
Immunotherapy	Metastatic cancer	Innate immune checkpoints, which interfere with the detection and clearance of malignant cells through phagocytosis and inhibit innate immune sensing, also have a key role in tumor-mediated immune escape and thus could be potential targets for cancer immunotherapy.	^300^
Immunotherapy	Metastatic non-squamous cell lung cancer	Bevacizumab plus atezolizumab plus chemotherapy both significantly improve progression-free survival and overall survival in patients.	^301^
Immunotherapy	Metastatic cancer	The combination regimen of Mn and anti-PD-1 antibodies demonstrated good efficacy in most patients with advanced metastatic solid tumors, demonstrating type I IFN induction, a controlled safety profile and a recovery response to immunotherapy.	^303^
Immunotherapy	Metastatic sarcoma	Evidence has been presented that durvalumab, an anti-PD-L1 drug, and tremelimumab, an anti-CTLA-4 drug, the combination of durvalumab and tremelimumab is an active treatment regimen for metastatic sarcoma.	^299^
Immunotherapy	Metastatic cancer	Immunotherapy based on the adoptive transfer of naturally occurring or gene-engineered T cells can be effective in treating patients with metastatic cancer.	^312^
Immunotherapy	Metastatic cervical cancer	Patients with metastatic cervical cancer experienced durable, complete regression after a single infusion of HPV-TILs.	^319^
Immunotherapy Tumor vaccine	Metastatic cancer	A vaccine targeting resistant tumors by dual T cell plus NK cell attack can be very helpful in the treatment of metastatic cancer: immunization after surgical removal of a highly metastatic primary tumor can inhibit the growth of subsequent metastases.	^322^
Immunotherapy and radiotherapy	Metastatic immune-cold tumors	Low-dose radiotherapy reprogrammed the tumor microenvironment with sparse immune infiltration while inducing an immune response to achieve tumor control	^324^
Immunotherapy and targeted therapy	Metastatic non-squamous NSCLC	Compared to chemotherapy alone, the addition of pembrolizumab to standardized chemotherapy with pemetrexed and a platinum-based agent had higher overall and progression-free survival.	^325^
Nanotechnology and operation	Metastatic cancer	Encapsulation of the drug in nanoparticles reduce toxicity while increasing the specificity and signal intensity of the imaging agent, allowing visualization of metastases during surgery.	^340^
Nanotechnology	Metastatic cancer	A supramolecular cationic gold nanorod can serve as a carrier to deliver CRISPR/Cas9 targeting PD-L1, thus improving immune checkpoint blockade (ICB).	^344^
Nanotechnology	Metastatic cancer	DNA nanorobotic systems can deliver drugs that have specific biological activity and cannot be used as conventional therapeutic agents for targeted tumor therapy.	^349^
Oncolytic virotherapy	Metastatic melanoma	OVs can act as immunotherapeutic agents alone or as fusion platforms for immunomodulation. FDA has approved the first OV for metastatic melanoma, talimogene laherparepvec (Imlygic).	^354^
Oncolytic virotherapy and immunotherapy	Melanoma	ACT of tumor-specific T cells has a highly advantageous effect when combined with OVs. Due to the improved T-cell effector function, combination therapy is far more effective than any of the monotherapies.	^358^
Oncolytic virotherapy and immunotherapy	Metastatic cancer	OVs can use created tumors as an in situ source for neoantigen vaccination, leading to regression of distant uninfected tumors through cross-presentation.	^360^
Antibody-drug conjugates	Breast cancer	Sacituzumab govitecan (SG) is an antibody-drug conjugate with an SN-38 payload targeting trophoblast cell-surface antigen 2, an epithelial antigen expressed in breast cancer, which demonstrated statistically significant progression-free survival benefit over chemotherapy.	^362^
Antibody-drug conjugates	HER2-positive breast cancer	An antibody-drug conjugate, trastuzumab deruxtecan, has shown high activity for the treatment of patients with metastatic HER2-positive breast cancer whose disease has progressed after multiple treatments.	^363^

**Table 4:** Clinical trials for metastatic cancer

Study	Regimen	Mechanism of action type	Disease status	Sample size	Median follow-up duration, months	mPFS, months	mOS, months	ORR,%	Reference number
NCT03438396 Coleman et al.	Tisotumabvedotin	Antibody-drug conjugate	Recurrent or metastatic cervical cancer	102	10.0	NA	NA	24	^444^
NCT02220894 Mok et al.	Pembrolizumab	PD-1 inhibitor	Untreated metastatic non-small-cell lung cancer with a programmed death ligand 1 (PD-L1) tumor proportion score (TPS) of 50% or greater	672	12.8	NA	20.0	NA	^445^
NCT02924376 Abou-Alfa et al.	Pemigatinib	Inhibitor of FGFR	Previously treated, locally advanced or metastatic cholangiocarcinoma	107	17.8	NA	NA	35.5	^446^
NCT00567190 Swain et al.	Pertuzumab, trastuzumab, and docetaxel	Monoclonal antibody and Chemotherapy	Metastatic breast cancer	452	99.9	NA	57.1	NA	^281^
NCT02425891 Schmid et al.	Atezolizumab plus nab-paclitaxel	Monoclonal antibody and Chemotherapy	Metastatic triple-negative breast cancer	451	18.5	NA	21.0	NA	^447^
NCT03789604 Zhou et al.	Sugemalimab plus chemotherapy	PD-1 inhibitor plus Chemotherapy	Metastatic non-small-cell lung cancer	320	17.8	9.0	NA	NA	^448^
2016-005241-23 Pfeiffer et al.	TAS-102 plus bevacizumab	Monoclonal antibody and Chemotherapy	Metastatic colorectal cancer	46	10.0	4.6	NA	NA	^449^
NCT02888743 Schoenfeld et al.	Durvalumab and tremelimumab and radiotherapy	PD-L1 and CTLA-4 inhibitors plus radiotherapy	Metastatic non-small-cell lung cancer	78	12.4	NA	NA	NA	^450^
NCT01942135 Cristofanilli et al.	Fulvestrant plus palbociclib	CDK4 and CDK6 inhibitor;estrogen receptor antagonists	Metastatic breast cancer	347	8.9	9.5	NA	NA	^451^
2012-002128-33 Sartore-Bianchi et al.	Trastuzumab and lapatinib	Anti-Her2 Monoclonal Antibodies and Chemotherapy	HER2-positive metastatic colorectal cancer	27	23.5	NA	NA	30	^280^
NCT02453282 Johnson et al.	Durvalumab and tremelimumab	PD-L1 and CTLA-4 inhibitors	Metastatic non-small-cell lung cancer	371	NA	3.9	11.9	NA	^452^
NCT03082534 Sacco et al.	Pembrolizumab and cetuximab	PD-1 inhibitor and EGFR inhibitor	Metastatic head and neck squamous cell carcinoma	33	7.3	NA	NA	45	^453^
NCT03048877 Lin et al.	Apatinib	VEGFR-2 inhibitor	Metastatic radioactive iodine-refractory differentiated thyroid cancer	46	18.1	22.2	not reached	54.3	^454^
NCT02365597 Siefker-Radtke et al.	Erdafitinib	Tyrosine kinase inhibitor	Metastatic urothelial carcinoma	101	11	NA	NA	40	^455^
NCT03787992 Shi et al.	Furmonertinib	EGFR tyrosine-kinase inhibitors	EGFR mutation-positive locally advanced or metastatic non-small-cell lung cancer	178	21	20.8	NA	NA	^456^
NCT00700102 Bennouna et al.	Bevacizumab plus fluoropyrimidine	Monoclonal antibody and Chemotherapy	Metastatic colorectal cancer	409	11.1	NA	11.2	NA	^457^
NCT03051659 Tolaney et al.	Eribulin plus pembrolizumab	Monoclonal antibody and Chemotherapy	(HR)-positive metastatic breast cancer	88	10.5	4.1	NA	27	^458^
NCT02574455 Bardia et al.	Sacituzumab govitecan	Antibody-drug conjugate	Relapsed or refractory metastatic triple-negative breast cancer	235	NA	5.6	12.1	35	^459^
NCT02492568\NCT02444741 Theelen et al.	Pembrolizumab with radiotherapy	PD-L1 inhibitors plus radiotherapy	Metastatic non-small-cell lung cancer	72	33	9	19.2	NA	^460^
NCT03260491 Jänne et al.	Patritumab Deruxtecan(HER3-DXd)	Antibody-drug conjugate	Metastatic EGFR-mutated non-small cell lung cancer	57	NA	8.2	NA	39	^461^
NCT03384940 Siena et al.	Trastuzumab deruxtecan	Antibody-drug conjugate	HER2-expressing metastatic colorectal cancer	53	6.77	NA	NA	45.3	^462^
NCT03504397 Shitara et al.	Zolbetuximab plus mFOLFOX6	Monoclonal antibody targeting CLDN18.2	Metastatic gastric or gastro-esophageal junction adenocarcinoma	283	12.94	10·6	NA	NA	^463^
NCT01103323 Grothey et al.	Regorafenib	Multikinase inhibitor	Documented metastatic colorectal cancer and progression during or within 3 months after the last standard therapy	760	NA	NA	6.4	NA	^464^
NCT00388726 Cortes et al.	Eribulin monotherapy	Non-taxane microtubule dynamics inhibitor	Recurrent or metastatic breast cancer	762	NA	NA	13.1	NA	^465^
NCT01492101 Perez et al.	Etirinotecan pegol	Long-acting topoisomerase-I inhibitor	Recurrent or metastatic breast cancer	852	NA	NA	12.4	NA	^466^
NCT02504372 O’Brien et al.	Pembrolizumab	PD-1 inhibitor	Stage IB-IIIA non-small-cell lung cancer	1177	35.6	NA	53.6	NA	^467^
NCT01305941 Van et al.	Everolimus, trastuzumab, and vinorelbine	Brain-permeable mTOR inhibitor	Progressive HER2-positive breast cancer brain metastases	26	3.9	NA	12.2	NA	^468^
NCT00284258 Muro et al.	Irinotecan plus oral S-1	A combination of tegafur, 5-chloro-2,4-dihydroxypyridine, and potassium oxonate	Metastatic colorectal cancer	426	12.9	5.8	NA	NA	^469^
NCT02711553 Valle et al.	Ramucirumab or merestinib	Targeted inhibitors	Histologically or cytologically confirmed, non-resectable, recurrent, or metastatic biliary tract adenocarcinoma	309	10.9	6.5/7.0	NA	NA	^470^
NCT02443324 Herbst et al.	Ramucirumab plus pembrolizumab	VEGF receptor-2 (VEGFR-2) and PD-1 or PD-L1 inhibitor	Advanced gastric or gastro-esophageal junction adenocarcinoma, non-small-cell lung cancer, or urothelial carcinoma	92	32.8	NA	NA	NA	^471^
NCT03202758 Thibaudin et al.	Durvalumab and tremelimumab with chemotherapy	Chemotherapy with targeted therapies	RAS-mutant unresectable metastatic CRC	57	NA	8.2	not reached	64.5	^472^
NCT01328171 Modest et al.	FOLFOXIRI Plus Panitumumab	Combination therapy	RAS Wild-type metastatic colorectal cancer	96	NA	NA	NA	87.3	^473^
NCT00094653 Hodi et al.	Ipilimumab	Block cytotoxic T-lymphocyte	Unresectable stage III or IV melanoma	676	NA	NA	10.1	NA	^474^
NCT02983045 Siefker-Radtke et al.	Bempegaldesleukin plus Nivolumab	Immune-stimulating prodrug plus the antibody	Metastatic urothelial carcinoma	41	NA	4.1	23.7	35	^475^
NCT01848834 Burtness et al.	Pembrolizumab	PD-1 inhibitor	Recurrent or metastatic squamous cell carcinoma of the head and neck	60	NA	12.2	13	NA	^476^
NCT01528618 Hong et al.	Gemcitabine plus cisplatin	Systemic chemotherapy	Recurrent or metastatic nasopharyngeal carcinoma	362	69.5	NA	22.1	NA	^477^
NCT01108445 Armstrong et al.	Sunitinib	VEGF receptor inhibitor	Metastatic non-clear cell renal cell carcinoma	108	8.3	NA	NA	NA	^478^

## Limitations of current research on cancer metastasis

### Drug resistance

A recurring challenge in treating metastatic tumors is the eventual development of resistance to treatments that were initially efficacious. The intricacies of cancer metastasis encompass a myriad of adaptive mechanisms, equipping metastatic cells with resilience in novel environments. Such mechanisms further bolster these cells against stresses induced by cytotoxic or targeted therapies, thereby elevating their resistance threshold. Once a tumor exhibits drug resistance, therapeutic agents diminish in efficacy or become entirely ineffective. This can precipitate swift cancer recurrence or disease progression, culminating in patient mortality. For instance, in the realm of signaling pathway activation, resistance to EGFR mAb like cetuximab and panitumumab in metastatic CRC can manifest via mutations in *KRAS*, *BRAF*, *PIK3CA*, or *PTEN*.^364^ Osimertinib, a third-generation EGFR-TKI, demonstrates notable efficacy against EGFR-mutated NSCLC. Nevertheless, resistance to osimertinib inevitably emerges, often attributed to the EGFRC797S mutation. In CRC, components of the tumor microenvironment such as Tregs, IL-10, TAMs, MDSCs, and associated cytokines can mediate resistance to PD1/PD-L1 blockade therapies.^365^ Notably, while combination therapies have been proposed as a means to circumvent resistance, the sheer number of potential combinations surpasses those subjected to clinical evaluation. Hence, immediate patient benefits might arise more from optimizing combinations of existing drugs than from identifying new drug targets.

The shortcomings of conventional cancer chemotherapy, characterized by low bioavailability, modest therapeutic efficacy increments, and indistinct side effects, have spurred considerable research into drug delivery systems (DDS). DDS, in theory, can amplify the efficacy of anticancer agents and curtail their systemic toxicity by enhancing targeted drug delivery. Signaling nodes that concurrently influence cancer metastasis and therapy resistance emergence are compelling therapeutic targets, as their inhibition can simultaneously impede both processes. Intensive research efforts have delved into the consequences of targeting these pathways, scrutinizing their impact on tumor cell survival, emergence of therapy resistance, and metastatic growth, utilizing both 3D tissue cultures and preclinical animal models. Furthermore, patient-derived organoids have been corroborated to faithfully replicate drug sensitivities observed in patients, affirming the feasibility of this approach.^366^

### Limited animal models

Animal models have provided significant insights into the mechanisms of cancer metastasis. However, certain limitations inherent to these models warrant consideration. Primarily, cancer biology in laboratory animals exhibits marked disparities compared to human cancers. Human tumors, when grown in animals, often proliferate at an accelerated rate relative to their counterparts in human patients.^367^ This differential growth trajectory complicates the extrapolation of preclinical data to clinical scenarios. Furthermore, the tumor stage in animal models frequently diverges from the clinical context in which interventions are assessed. For instance, tumor localization in models, such as subcutaneous xenografts, may not accurately represent a patient’s tumor site. Another limitation lies in the predominant lack of a tumor microenvironment in many organoid animal models. The ascendancy of immunotherapy underscores the urgency for developing immunogenic mouse models through genetic engineering. However, these models are not without shortcomings. Human metastatic cancers typically present a more extensive mutational burden compared to genetically engineered mouse tumors. The extent to which even the most advanced mouse models emulate the neoantigen landscape and immune contexture in patients with progressive metastatic disease remains uncertain. The complexity and substantial costs associated with these models have led to their underutilization, leaving open the question of their fidelity in representing human malignancies. Additionally, the investigation of cancer cell dormancy in animal models presents challenges, as dormant cells may require extended durations to reactivate. The brevity of many animal studies hampers a thorough exploration of dormancy and subsequent reactivation. While animal models continue to be indispensable in metastasis research, it remains crucial to acknowledge their constraints and to complement them with alternative methodologies for a holistic understanding of cancer progression.

### Lack of promising biomarkers and early detection methods

The criticality of pinpointing biomarkers to predict metastatic propensity or to monitor its trajectory is evident. Nonetheless, progress in the identification and validation of credible metastasis biomarkers remains protracted, thereby impeding both early diagnosis and therapeutic surveillance. Approximately half of diagnosed cancers manifest in advanced stages with metastasis to distant organs already evident. The paucity of early detection methodologies for metastatic cancer exacerbates the challenges of efficacious treatment.

Biomarkers for incipient cancer manifest as both biochemical and discernible structural changes in tissue. Optimal approaches lean towards minimally invasive sampling, encompassing imaging modalities and fluid biopsies like blood, saliva, or urine.^368^ Liquid biopsies, including circulating tumor DNA (ctDNA), enable the recognition of diverse cancer indicators. ctDNA analyses, particularly for patients with advanced malignancies where ctDNA concentrations are substantial, have showcased potential in bespoke mutation profiling and patient monitoring.^369^ However, a salient challenge is the markedly diminished concentrations of ctDNA and other biochemical cancer biomarkers in initial-stage malignancies. To surmount this, there’s an imperative to refine existing detection thresholds. The sequencing of the human genome has illuminated the landscape of the cancer genome, delineating patterns of genetic aberrations in malignancies. Such patterns can underpin cancer detection, stratification, and therapeutic approaches. Potential diagnostic biomarkers extend to CTCs, exosomes, cellular fusions, metabolites, and proteins. It’s conceivable that multimodal assessments might enhance sensitivity and specificity in early cancer detection compared to mono-biomarker evaluations. A pivotal challenge lies in developing methodologies with the acumen for ultra-early cancer detection and the precision to curtail false positives. An overarching objective in early detection is to discern nascent solid tumors when they are still amenable to treatment and unlikely to have metastasized, typically when these tumors are approximately one millimeter in diameter (encompassing 10^^5^ to 10^^6^ cells). Most extant or developmental imaging systems lack the resolution for such minuscule tumor visualization. Nonetheless, avant-garde in vivo imaging platforms, such as the 10.5 T MRI,^370^ persistently endeavor to redefine these boundaries.

### Heterogeneity

Cancer metastasis manifests with pronounced heterogeneity. Notably, disparate cancers, and even individual metastatic sites within the same patient, can display variegated genetic and molecular configurations. Such heterogeneity complicates the crafting of targeted therapies with universal efficacy across the spectrum of metastatic malignancies. There exists considerable evidence supporting the notion that intratumoral heterogeneity (ITH) fortifies tumors against conventional chemotherapy, radiation, and specific anticancer agents. Furthermore, ITH considerably modulates the efficacy of numerous immunotherapies, notably ICIs.^371^ Beyond influencing ICI potency, ITH also muddles clinically oriented prognostications concerning disease outcomes. ICI’s influence on ITH, particularly alterations in its specificity, foretells the magnitude and persistence of antitumor reactions. Summarily, accumulating evidence indicates that heightened ITH adversely modulates clinical responses to diverse treatments, encompassing immunotherapy. This underscores the potential of ITH as a candidate for devising combination therapeutic strategies. Nevertheless, while spatial ITH within the tumor microenvironment may theoretically be targetable, temporal ITH, unless entirely eradicated, might emerge and spur relentless disease progression in the milieu of therapeutic resistance.^372^

## Conclusion and perspective

Addressing drug resistance remains paramount, with combinations of anticancer agents proposed as potential strategies to counteract or delay the manifestation of multiple resistance mechanisms. Moreover, there is a growing interest in preventing the expulsion of anticancer drugs from cancer cells. The ATP-binding cassette (ABC) transporter protein, which expels anticancer drugs from cells, is currently under intensive investigation. The scientific community is working assiduously to devise next-generation ABC transporter inhibitors to ameliorate drug resistance. From an epigenetic standpoint, drug resistance is postulated to arise from modifications in the epigenetic architecture of tumor cells. Rectifying such reversible alterations holds promise in countering this resistance. A confluence of signaling pathways, such as integrin binding, stress response, and metabolic reprogramming, underpins both cancer metastasis and drug resistance genesis. These central nodes in the signaling milieu may herald innovative avenues for bolstering anticancer interventions. The co-occurrence of these signaling hubs in both metastatic progression and therapeutic resistance renders them prime candidates for therapeutic targeting. Rigorous research endeavors are directed towards understanding the ramifications of targeting these pathways on tumor resilience, therapeutic resistance, and metastatic proliferation, both in three-dimensional tissue cultures and preclinical animal paradigms. Given the immunosuppressive aura generated by tumors, the tumor microenvironment emerges as a potential target. Emerging research has spotlighted nodes within metastatic and resistance-associated signaling architectures, suggesting a pivot from conventional strategies. This could lead to enhanced anticancer regimens, specifically focusing on nodal aberrations and metastatic entities. Advances in scRNA-seq and genomic evaluations further elucidate the intricate adaptive networks within the TME, shedding light on the nexus between resistance and tumor proliferation, underscoring the importance of precision therapeutic modalities.

Concurrently, the push for personalized cancer management aims to fine-tune drug screenings, ensuring tailored interventions and minimizing drug resistance. The sheer volume of drug combinations requisite for patient-specific optimization presents a daunting challenge. Herein, artificial intelligence emerges as a potential asset to bolster research endeavors in this domain. Nanobots represent an avant-garde drug delivery paradigm, enhancing drug efficiency and enabling precise cancer treatment. Active tumor cells serve as bespoke targets for this drug deployment, ensuring optimal tumor targeting while safeguarding surrounding healthy tissue and organ integrity. This ensures heightened therapeutic efficiency coupled with minimized adverse effects. EVs, especially exosomes, are gaining traction for their prospective utility as drug delivery conduits. Characterized by their reduced toxicity, elevated biocompatibility, low immunogenicity, and a protective phospholipid bilayer, EVs promise sustained drug action. Their intrinsic affinity for specific parent cell receptors lends them a degree of targeted delivery. Furthermore, their amenability to artificial modifications augments their therapeutic functionality.

The patient-derived tumor xenograft (PDX) model involves transplanting primary cells from patient-derived tumor tissue into immunodeficient mice. This model preserves the majority of the histopathological, molecular biological, and genetic features of the original tumor. Due to its ability to closely predict clinical outcomes, the PDX model is gaining prominence in pivotal stages of new drug development, including drug screening. Utilizing humanized animal models as PDX hosts addresses the significant limitations associated with immunodeficient mice. Anticipations are that PDX models will yield valuable insights in the domains of antitumor drug screening, clinical dosing recommendations, and individualized tumor patient treatment in the coming years.

Furthermore, there is a growing emphasis on integrating multi-omics and single-cell sequencing technologies into clinical trial designs. Such integration can offer in-depth clarity on cellular and molecular effects of localized treatment responses and metastasis co-occurrence. Mastery of expansive genetic data sets is imperative to formulate personalized, multi-modal therapeutic approaches that can effectively counteract tumor metastasis and resistance mechanisms in patients.

Tumor metastasis is a multifaceted disease, encompassing more than just a singular organ or gene anomaly. It embodies a cascade of alterations, necessitating a holistic analytical approach. In this review, we have delineated the genetic shifts and prospective therapeutic interventions for tumor metastasis from various vantage points. As we progress toward clinical implementation, it’s imperative not to narrow our focus solely to the tumor but to incorporate an encompassing patient evaluation. Factors such as individual organ health, the interrelation between primary and metastatic lesions, and potential side effects must be considered. An amalgamation of gene-specific treatments and a systemic holistic approach could potentially represent the most efficacious strategy for managing cancer metastasis.
